# Adaptive Localization for Underwater Nodes in Uncertain Environments: A Geometric Topology Perception-Enhanced Multi-Stage Reinforcement Learning Strategy

**DOI:** 10.3390/s26175631

**Published:** 2026-09-04

**Authors:** Lijun Hao, Chunbo Ma, Jianbo Cui, Jun Ao

**Affiliations:** School of Information and Communication Engineering, Guilin University of Electronic Technology, Guilin 541004, China; 23021201006@guet.edu.cn (L.H.); junjunao1@263.net (J.A.)

**Keywords:** underwater wireless sensor networks, uncertainty quantification, adaptive localization, reinforcement learning, geometric topology perception, multi-stage reward

## Abstract

Complex underwater environments induce difficult-to-quantify ranging errors, constraining the localization accuracy and robustness of heterogeneous networks. To address this, a node localization method based on a Geometric Topology Perception-Enhanced Multi-Stage Reinforcement Learning Strategy is proposed. First, an uncertainty quantification model under multi-source interference is established to characterize time-varying noise and accurately quantify the ranging errors of heterogeneous links. Subsequently, using the resulting ranging variance, an adaptive weight allocation mechanism based on Minimum Variance Unbiased Estimation is constructed to dynamically adjust link weights, achieving the robust fusion of multi-modal observation data. Finally, a Weighted Least Squares objective function is formulated, and the GP-AC strategy is developed. By utilizing Gaussian Process Regression and local Geometric Dilution of Precision, a multi-stage reward mechanism is constructed to circumvent topological traps and accurately estimate the single-epoch three-dimensional coordinates of static or quasi-static underwater sensor nodes. Simulation results demonstrate that system robustness is improved by 91.9%, average accuracy is enhanced by 54.9%, and the measured average localization time is 7.45 s.

## 1. Introduction

As the core infrastructure for exploring deep-sea resources and constructing the Internet of Underwater Things (IoUT), Underwater Wireless Sensor Networks (UWSNs) play an irreplaceable and critical role in fields such as continuous marine environmental monitoring, disaster early warning, resource exploration, and national defense security [1,2,3]. In these complex application systems, the underlying monitoring data collected by sensor nodes, such as water quality, temperature, and ocean currents, are endowed with practical scientific analysis value only when deeply bound with their accurate geographic three-dimensional (3D) coordinates [4,5]. Meanwhile, high-precision location information is also considered a fundamental prerequisite for the realization of efficient routing, topology control, and Medium Access Control (MAC) within the network [6]. However, since the high-frequency radio waves relied upon by the Global Positioning System (GPS) are extremely susceptible to severe attenuation in the underwater medium, traditional terrestrial localization technologies are rendered fundamentally ineffective in underwater environments [7]. Therefore, the exploration of high-precision 3D localization technologies for internal nodes suitable for complex deep-water environments has emerged as a fundamental challenge that urgently needs to be addressed in the current fields of marine communications and underwater acoustic networks.

Nevertheless, the following problems still exist when highly time-varying and complex deep-sea environments are confronted: (1) Underlying physical ranging errors are difficult to quantify and isolate accurately. It is mostly assumed by existing methods that underwater environmental noise follows a fixed and stationary distribution; thus, sudden and long-tail NLOS errors induced by sea surface wind and waves, abrupt changes in water turbidity, and so forth are difficult to perceive in real time and quantify accurately. Due to the lack of a high-precision uncertainty quantification model based on physical channels, adverse observation data cannot be effectively and physically isolated by the system. (2) A dynamic, robust, and adaptive mechanism is absent in the fusion of multi-source heterogeneous links. Empirically fixed weight allocations or simple mean fusion are frequently relied upon by existing heterogeneous data fusion strategies. In scenarios with severe environmental fluctuations, channel states cannot be perceived in real time and the confidence weights of heterogeneous links cannot be dynamically adjusted by the system. Once sudden high-variance noise is encountered by a specific link (e.g., an optical link encountering turbid water), the global ranging results are extremely prone to being contaminated. (3) Traditional global optimization algorithms are prone to falling into traps of ill-conditioned topologies and local minima. When 3D coordinates are inversely solved based on fused distances, traditional heuristic algorithms rely heavily on population-based parallel optimization, which incurs substantial computational overhead and is prone to local stagnation on highly non-convex and densely pitted underwater error surfaces. Meanwhile, under complex and variable node topologies (e.g., coplanar and aggregated configurations), the perception and circumvention of spatial geometric distortions cannot be achieved by conventional RL.

To address the aforementioned challenges posed by environmental uncertainty, a node localization method based on Geometric Topology Perception-Enhanced Reinforcement Learning (GP-AC) is proposed to meet the joint optimization requirements of localization accuracy and robustness for single-epoch three-dimensional localization of static or quasi-static nodes in complex underwater environments. Unlike existing studies in which environmental error modeling, heterogeneous measurement fusion, and coordinate optimization are performed independently, the proposed framework propagates the environment-derived ranging variance as a unified confidence prior through MVUE fusion, WLS coordinate estimation, RL state construction, and weighted-GDOP reward shaping. Meanwhile, a residual- and iteration-driven switching mechanism is introduced to adaptively shift the optimization focus from global error reduction to local topology-aware refinement. The main contributions of this study are summarized as follows:

(1) To address the difficulty in accurately quantifying underwater channel ranging errors, a high-precision ranging error quantification model under multi-source interference uncertainty is established. By characterizing the time-varying noise distribution dominated by wind, waves, and water turbidity, a quantitative representation of the ranging error variance is achieved. The principal advantage of this model lies in its ability to isolate high-variance, low-quality measurements and suppress the propagation of extreme long-tailed noise to the computational layer, thereby providing highly reliable confidence indicators for multi-source fusion.
(2) To mitigate the susceptibility of heterogeneous networks to environmental interference, an adaptive weighting mechanism based on Minimum Variance Unbiased Estimation (MVUE) is constructed by taking the ranging variance obtained from the uncertainty quantification model as its input. The fusion weights are dynamically adjusted by this mechanism, thereby enabling efficient and robust fusion of multimodal measurements. Its advantage lies in exploiting the complementary characteristics of acoustic and optical links while overcoming the error amplification caused by fixed-weight fusion. Under compound disturbance conditions jointly induced by high wind speed and high water turbidity, the proposed mechanism can adaptively suppress simultaneous increases in acoustic and optical ranging errors and stably obtain reliable fused distance estimates.
(3) To address the tendency of non-convex underwater localization models to become trapped in local extrema and ill-conditioned optimization regions, a GP-AC-based optimization algorithm is proposed. Gaussian Process Temporal Difference (GPTD) learning is incorporated to quantify epistemic uncertainty and enable uncertainty-guided exploration. By combining a geometric topology-aware reward mechanism with Natural Policy Gradient (NPG) updates, the proposed algorithm enhances manifold optimization and improves its ability to avoid local stagnation induced by unfavorable spatial geometries. Its main advantage lies in achieving high localization accuracy. Through topology-adaptive coordinate updates, the proposed method improves the capability to avoid local extrema and the stability of iterative convergence while effectively suppressing numerical oscillations, thereby providing an efficient optimization tool for accurate three-dimensional coordinate estimation of underwater nodes.

## 2. Related Work

To address the issue where a substantial number of Non-Line-Of-Sight (NLOS) errors are induced in underlying ranging by extremely complex underwater acoustic–optical physical channels and are difficult to quantify accurately, numerous scholars have conducted extensive beneficial explorations from the perspectives of physical-layer signal processing and error compensation mechanisms. Kaur et al. [8] proposed a flexible localization protocol based on a hybrid reward evaluation mechanism, by which the reliability of node data interaction in dynamic network environments was effectively improved; however, further optimization is still required regarding its adaptive resistance to multipath errors under extremely harsh channels. Kim et al. [9] designed a 3D localization scheme for heterogeneous underwater networks based on hybrid Time of Arrival (TOA) and Angle of Arrival (AOA) measurements. Through this scheme, the survival rate and overall localization accuracy of the network under random attacks were significantly enhanced; nevertheless, the real-time dynamic processing mechanism for NLOS errors in its physical model remains insufficient. To resolve the issue of measurement absence caused by asynchronous clocks of mobile nodes, Liu et al. [10] introduced the Localization with Intersecting Time Measurements (LITM) algorithm combined with beacon departure time. Although the dilemma of insufficient TOA measurements was effectively addressed by this approach, the compensation for acoustic ray bending under complex constant-gradient sound speed profiles is considered relatively simplistic. By utilizing compact receiving arrays and channel feature matching technologies, Wu et al. [11] corrected Time Difference of Arrival (TDOA) values, and high-precision point-to-point localization was successfully achieved in time-varying channels; however, due to the high hardware requirements associated with this method, extending it to large-scale, low-cost underwater sensor clusters poses deployment-cost challenges. In addition, targeting the stratification effect of underwater acoustic wave propagation, Yu et al. [12] and Zhao et al. [13] investigated asynchronous localization algorithms combined with sound speed error estimation and constant-gradient sound speed profiles, respectively. Although the coordinate deviations caused by the stratification effect were mitigated, their iterative linearization processes are highly dependent on initial values. By extending the Doppler frame length via first-order Taylor expansion, Su et al. [14] further improved localization accuracy in far-field environments with low Signal-to-Noise Ratios (SNRs), but an uncertainty-based cross-validation for long-tail errors induced by dynamic ocean currents is lacking. Meanwhile, Pu et al. [15] and Qin et al. [16] respectively explored the correction of drift errors caused by node mobility utilizing neural networks and joint time synchronization models; Qin et al. [17] and Zhan et al. [18] thoroughly investigated the mitigation of multi-layer propagation deviations utilizing constant-gradient sound speed profile models; and Liu et al. [19] and Gong et al. [20] focused their efforts on error mitigation under hybrid NLOS conditions and the modeling of underlying ranging limit theories. Overall, although existing methods have achieved promising results in mitigating multipath effects, acoustic ray bending, and node mobility errors, environmental noise is often assumed to follow a fixed or stationary Gaussian distribution under highly time-varying underwater channel conditions. The reason for the poor performance of such static error models is that these models have difficulty perceiving and accurately quantifying sudden ranging errors caused by severe hydrological fluctuations (e.g., surface wind and waves, abrupt changes in water turbidity, and shipping noise) in real time. If a high-precision physical quantification model of uncertainty cannot be established to characterize the dynamic distribution of time-varying noise prior to the localization calculation, accurate confidence evaluation and physical isolation of adverse observation data cannot be performed by the system, and ultimately, deviations and collapses in coordinate estimation will be directly caused by the massive residual ranging errors.

Meanwhile, considering the issue that multi-source heterogeneous networks are highly susceptible to interference and severe fluctuations in scenarios with environmental uncertainty, how to perceive channel states in real-time and dynamically adjust the confidence weights of links to achieve efficient and robust fusion of multi-modal heterogeneous observation data—thereby obtaining highly reliable fused observation distances—is considered another core challenge in realizing high-precision localization. It is difficult for a single underwater acoustic or optical communication link to maintain high precision across all sea states, as its performance is constrained by physical bandwidth and environmental attenuation characteristics. To this end, Zhang et al. [21] constructed a dual-hop system integrating wireless optical and acoustic communications, by which the localization accuracy in local clear areas was greatly improved; however, the optical link attenuation problem in long-distance turbid water bodies still needs to be compensated by a weight-adaptive mechanism. Zhang Tengxiao et al. [22] proposed a dynamic optical localization algorithm based on optimal anchor node combination selection, and the influence of optical attenuation was effectively overcome; nevertheless, the joint environmental variance evaluation model during the concurrency of acoustic and optical links remains to be further refined. Yue et al. [23] constructed an intelligent Autonomous Underwater Vehicle-Internet of Underwater Things (AUV-IoUT) topological localization architecture utilizing a Double Deep Q-Network (DDQN), whereby localization latency and overall energy consumption were reduced; however, widespread deployment on underlying low-cost sensor nodes is limited by the offline training costs and online inference latency of complex DRL models. Kumar et al. [24] enhanced target localization capabilities based on proximity-driven Recurrent Neural Networks (RNNs), demonstrating excellent performance in reducing the average estimation error; nonetheless, the real-time operation of complex black-box models is still considered strenuous in underwater micro-nodes with extremely constrained computational power. Wang et al. [25] performed multi-sensor fusion utilizing an Unscented Kalman Filter (UKF) on manifolds, achieving smooth trajectory estimation; however, the soft isolation mechanism under sudden extreme interference could be further refined. Li et al. [26] designed a multi-AUV collaborative localization method based on the maximum entropy principle, effectively addressing model mismatches and measurement biases; yet, a certain risk of congestion in low-bandwidth underwater acoustic channels is presented by its belief propagation-based message-passing mechanism. Xian et al. [27] designed a coarse-to-fine 3D localization algorithm in combination with time-varying path loss, but a relatively static weighting strategy is adopted when heterogeneous data are fused. Additionally, Zou et al. [28], Jiang et al. [29], Shenbagharaman et al. [30], Li et al. [31], Praveena et al. [32], and Kaveripakam et al. [33] also made active contributions in low-cost cluster collaboration, bearing measurement, feature extraction, and algorithm clustering. Through the introduction of means such as multi-modal communication or machine learning, the aforementioned literature has effectively improved the ranging accuracy of the network within local areas. However, the reason they are prone to failure in complex and dynamic real marine environments is that a “real-time environmental perception” capability regarding the underlying physical channel states is generally lacking. When random channel fading or even malicious interference is encountered, existing studies frequently rely upon empirical and fixed weight allocations or simple mean fusion strategies. The credibility of information cannot be dynamically determined by such static fusion mechanisms according to the real-time physical SNR of each link. Once a severe environment is encountered by a certain heterogeneous link (e.g., an optical link suddenly encountering a highly turbid water mass), the fusion layer will be easily penetrated by high-variance noise, thereby contaminating the global results. Therefore, the lack of a dynamic confidence allocation and robust fusion mechanism based on real-time perception is considered the fundamental reason why highly reliable ranging distances are difficult to obtain by current heterogeneous networks.

After the underlying fused ranging information is obtained, global optimization solving needs to be conducted to address the inherent characteristics of multiple local extrema and high non-convexity in the localization problem, thereby acquiring the optimal 3D spatial coordinates of the unknown node to be localized. Traditionally, bio-inspired algorithms (such as particle swarm optimization and whale optimization algorithms) have been widely applied to solve complex nonlinear localization equations due to their derivative-free global search characteristics. However, these heuristic methods rely highly upon massive population parallel iterations, and premature convergence and local stagnation are extremely prone to occur when highly non-convex and densely pitted ranging error surfaces underwater are confronted. Therefore, aiming at the challenges of ranging errors and passive localization energy consumption caused by complex underwater environments, Liao et al. [34] introduced the Reinforcement Learning (RL) algorithm, through which energy-efficient passive localization of hidden mobile nodes underwater was realized via sequential trial-and-error interactions between the agent and the environment; nevertheless, the adaptive scheduling capability for dynamic anchor nodes is lacking in this passive mechanism when underwater acoustic sensor networks requiring active interaction are faced. To address this deficiency in scheduling capability, Liao et al. [35] further proposed an RL-based mobile node localization framework. By optimizing the selection strategy of anchor nodes, the target localization accuracy in complex underwater acoustic networks was effectively improved; meanwhile, targeting the pain points of model uncertainty brought about by asynchronous clocks and physical medium stratification effects underwater, Yan et al. [36,37] designed an RL-based asynchronous localization scheme with a sampling compensation mechanism, by which the defect of traditional least squares methods being prone to falling into local optima was successfully circumvented. Furthermore, with the increasing complexity of underwater tasks, a collapse in timeliness is often exhibited by single-agent localization algorithms when multi-UUV (Unmanned Underwater Vehicle) collaborative scenarios are encountered. Aiming at this concurrent multi-target problem, Yue et al. [38] developed a multi-UUV collaborative RL localization mechanism. By jointly optimizing signal weights and spatial grid positions, the localization robustness under extreme acoustic channels was significantly enhanced; to resolve the crisis of high beacon energy consumption triggered by dense interactions among multiple nodes, Liu et al. [39] proposed a beacon-assisted RL localization scheme. By dynamically selecting the optimal beacon and adaptively adjusting the transmission power, a perfect tradeoff between localization accuracy and total system energy consumption was achieved; however, severe risks of privacy leakage and eavesdropping are subsequently exposed by frequent beacon acoustic broadcasts. In order to ensure communication security while improving localization efficiency, Fan et al. [40] introduced an RL secure localization protocol combined with collaborative beamforming, by which security requirements and energy consumption were successfully transformed into joint optimization objectives, and eavesdropping from external malicious nodes was effectively resisted.

## 3. Preliminaries

### 3.1. Heterogeneous Network Architecture and Geometric Graph Model

Before the detailed derivation of the node localization algorithm and the calculation of ranging errors, the complex underwater physical environment and network structure must first be mathematically described. To overcome the limited bandwidth, high propagation delay, and severe multipath interference of a single underwater acoustic link, this study considers a three-dimensional heterogeneous sensor network integrating underwater acoustic communication and underwater optical wireless communication (UOWC).

In this system, the network is primarily composed of GPS-equipped surface buoy nodes, underwater anchor nodes with known coordinates, and unknown nodes to be localized. Each unknown node acquires acoustic and optical observations from neighboring anchor nodes for heterogeneous ranging and coordinate estimation. The localization considered in this study is single-epoch static or quasi-static three-dimensional node localization rather than submarine or AUV trajectory tracking. During each localization interval, the coordinate of the unknown node is assumed to remain unchanged, whereas the environmental conditions and the uncertainties of the acoustic and optical ranging observations may vary. No kinematic model involving velocity, acceleration, or heading is incorporated, and continuous dynamic-state estimation is beyond the scope of the present study.

To support the subsequent uncertainty modeling and GP-AC optimization, the underwater localization problem is represented as a weighted connected-graph estimation problem in a three-dimensional Euclidean space. Based on this architecture, the network topology, feasible localization region, and physical inter-node distances are defined below.

**Figure 1:**
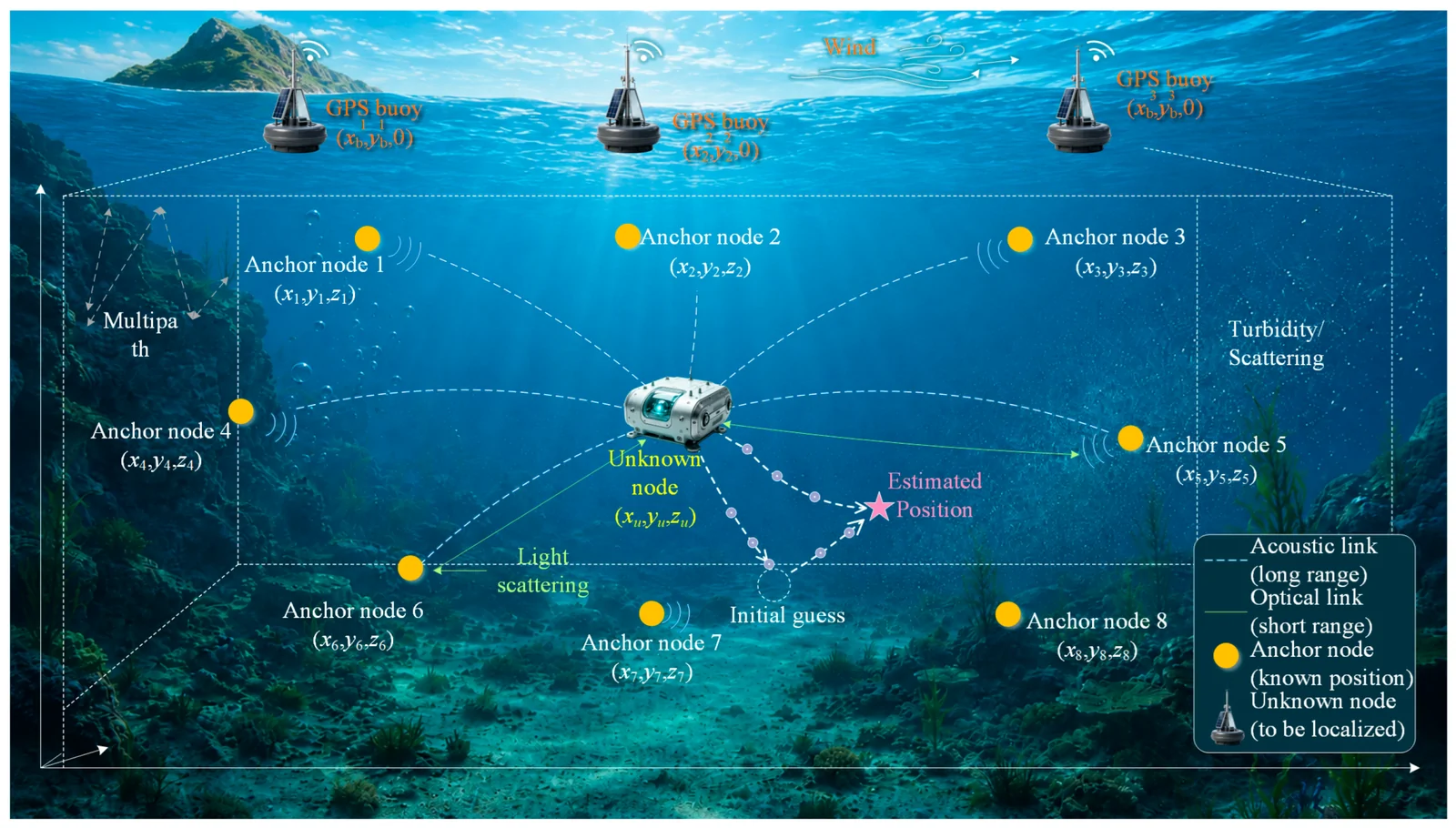
Example 3D topology of the proposed underwater acoustic–optical cooperative localization framework.

To provide an intuitive illustration of the considered underwater localization scenario, an example three-dimensional topology of the proposed acoustic–optical cooperative localization framework is presented in Figure 1. The topology consists of GPS-equipped surface buoys, multiple underwater anchor nodes with known coordinates, and an unknown node to be localized. Acoustic and optical ranging links are established between the unknown node and the available anchor nodes, while environmental disturbances such as wind, multipath propagation, turbidity, and light scattering are also considered.

#### 3.1.1. Topology Definition

The heterogeneous graph is defined as G=(V,E,P), where the node set V is composed of two parts:

(1) $$V=A\cup U=\left\{1,\ldots ,M\right\}\cup \left\{M+1,\ldots ,N\right\}$$

where A is the Anchor Set, and its position coordinate matrix $P_{A}\in R^{3\times M}$ is known; U is the Unknown Set (the set of nodes to be localized), and its coordinate matrix $X_{U}\in R^{3\times \left(N-M\right)}$ is the variable to be solved.

(2) $$x_{i}={\left[x_{i},y_{i},z_{i}\right]}^{T}\in R^{3},\;\forall i\in U$$

#### 3.1.2. Feasible Localization Region

To ensure that the coordinate estimation remains within the monitored underwater volume, the feasible localization region is defined as:

(3) $$\Omega =\left\{x={\left[x,y,z\right]}^{T}\in R^{3}\left|x_{min}\leq x\leq x_{max},y_{min}\leq y\leq y_{max},z_{min}\leq z\leq z_{max}\right.\right\}$$

where $x_{min},x_{max},y_{min},y_{max},z_{min},z_{max}$ denote the spatial boundaries of the monitored underwater region. The true coordinates of the unknown nodes and the candidate coordinates generated during GP-AC optimization are constrained within Ω. This feasible region is subsequently used to bound the continuous MDP state space and to project each coordinate update back into the valid underwater region.

#### 3.1.3. Physical Distance Definition

Each link $\left(i,j\right)$ in the edge set E corresponds to an objectively existing true physical Euclidean distance $d_{ij}$:

(4) $$d_{ij}={\left\|p_{i}-p_{j}\right\|}_{2}=\sqrt{{\left(x_{i}-x_{j}\right)}^{2}+{\left(y_{i}-y_{j}\right)}^{2}+{\left(z_{i}-z_{j}\right)}^{2}}$$

Although $d_{ij}$ is unknown, it is considered the core independent variable that determines all subsequent physical-layer parameters, such as delay, attenuation, and SNR.

### 3.2. Physical Layer Heterogeneous Ranging Mechanism and Signal Processing

In this section, the analytical mapping relationship among the physical signal waveform, channel impulse response, and observation distance $z_{ij}$ is established.

#### 3.2.1. Acoustic Ranging

Linear Frequency Modulation (LFM) spread spectrum signals are adopted by the acoustic links to resist multipath effects. Assuming the time-domain expression of the LFM signal $s\left(t\right)$ transmitted by source node i is:

(5) $$s\left(t\right)=\mathrm{rect}\left(\frac{t}{T_{s}}\right)\exp\left(j2\pi \left(f_{c}t+\frac{1}{2}K_{chirp}t^{2}\right)\right)$$

where $T_{s}$ is the signal period, $f_{c}$ is the carrier frequency, Kchirp=BTs is the frequency modulation slope, and B is the signal bandwidth. After the signal is transmitted through the underwater acoustic multipath channel, the received signal $y_{ij}\left(t\right)$ is modeled as the superposition of L paths:

(6) $$y_{ij}\left(t\right)=\sum_{l=0}^{L-1}\beta_{ij,l}\cdot s\left(t-\tau_{ij,l}\right)+n_{ac}\left(t\right)$$

where $\beta_{ij,l}$ and $\tau_{ij,l}$ denote the amplitude attenuation and time delay of the l-th path, respectively. The main path (Line-of-Sight, LoS, l = 0) component is focused on:

(7) $$\tau_{ij,0}=\frac{d_{ij}}{v_{sound}}$$

The squared modulus of the main path attenuation factor $\beta_{ij,0}$ is determined by the passive sonar equation:

(8) $${\left|\beta_{ij,0}\right|}^{2}=\frac{P_{rx}}{P_{tx}}=d_{ij}^{-\kappa }\cdot 10^{-\frac{\alpha_{abs}\left(f\right)\cdot d_{ij}}{1000\cdot 10}}$$

Matched Filtering (MF) is performed on $y_{ij}\left(t\right)$ by the receiver, and the cross-correlation function $R_{ys}\left(\tau \right)$ is defined as:

(9) $$R_{ys}\left(\tau \right)=\int_{-\infty }^{+\infty }y_{ij}\left(t\right)s^{*}\left(t-\tau \right)dt$$

The estimated time delay ${\hat{\tau }}_{ij}$ is determined by the peak of the cross-correlation:

(10) $${\hat{\tau }}_{ij}=\arg\underset{\tau }{\max\left|R_{ys}\left(\tau \right)\right|}$$

Let the matched-filter delay estimate be expressed as ${\hat{\tau }}_{ij}=\tau_{ij}+\varepsilon_{\tau ,ij}$ where $\varepsilon_{\tau ,ij}$ denotes the random delay-estimation error. For the deterministic LFM signal in Equation (5), the Fisher information associated with the propagation delay is determined by the effective RMS bandwidth and the matched-filter SNR. The effective RMS bandwidth is defined as

(10a) $$\beta_{rms}^{2}=\frac{\int_{-\infty }^{+\infty }{\left(f-\bar{f}\right)}^{2}\left|S{\left(f\right)}^{2}\right|df}{\int_{-\infty }^{+\infty }{\left|S(f)\right|}^{2}df}$$

where $\bar{f}$ is the spectral centroid and $\beta_{rms}^{2}$ has units of Hz^2^. Under the adopted complex-baseband signal convention, the CRLB of the matched-filter TOA estimate is

(10b) $$\mathrm{Var}\left(\varepsilon_{\tau ,ij}\right)\geq \frac{1}{8\pi^{2}\beta_{rms}^{2}\Gamma_{\mathrm{MF},ij}}$$

where $\Gamma_{\mathrm{MF},ij}$ is the dimensionless linear SNR associated with the received signal energy at the matched-filter input. For an approximately rectangular LFM spectrum with bandwidth *B*, $\beta_{rms}=B/\sqrt{12}$. Because one-way TOA ranging is adopted in Equation (7), the corresponding matched-filter ranging variance is

(10c) $$\sigma_{\mathrm{MF},ij}^{2}=v_{s}^{2}\mathrm{Var}\left(\varepsilon_{r,ij}\right)\geq \frac{v_{s}^{2}}{8\pi^{2}\beta_{\mathrm{rms}}^{2}\Gamma_{\mathrm{MF},ij}}$$

where $v_{s}$ is measured in m/s, $\mathrm{Var}\left(\varepsilon_{r,ij}\right)$ is measured in $s^{2}$, and $\sigma_{\mathrm{MF},ij}^{2}$ is measured in $m^{2}$.

Accordingly, under the one-way TOA model adopted in Equation (7), the acoustic observation distance $z_{ac}$ is expressed as:

(11) $$z_{ac}=v_{sound}\cdot {\hat{\tau }}_{ij}=d_{ij}+n_{ToA}$$

where $n_{ToA}=v_{s}\varepsilon_{\tau ,ij}$ denotes the matched-filter ranging error in meters, and its ideal variance is bounded by Equation (10c).

#### 3.2.2. Optical Ranging

For optical links, ranging based on Received Signal Strength (RSS) is utilized.

1. Geometric Optical Transmission Model

The normal vector of the transmitting node $n_{i}$ and the normal vector of the receiving node $n_{j}$ are defined. Based on the Lambertian radiant source model, the emission angle ϕ and the reception angle ψ satisfy:

(12) $$\cos\phi =\frac{n_{i}^{T}\left(x_{j}-x_{i}\right)}{d_{ij}},\;\cos\psi =\frac{n_{j}^{T}\left(x_{i}-x_{j}\right)}{d_{ij}}$$

The Lambertian mode number m is determined by the half-power angle of the light source $\Phi_{1/2}$:

(13) $$m=\frac{-\ln2}{\ln\left(\cos\Phi_{1/2}\right)}$$

2. Alignment Assumption and Transcendental Equation

Assuming that LoS alignment is achieved during the ranging phase (ϕ≈0, ψ≈0), the received optical power $P_{rx}$ is simplified as follows:

The optical RSS model is conditioned on a detectable LoS ranging link and does not assume continuous perfect alignment during node motion. It describes a short ranging interval in which the selected optical anchor and the unknown node maintain an available LoS link with sufficiently small emission and reception angles. The feasibility of LED-based underwater optical communication and positioning with mobile nodes has been experimentally demonstrated in [21], while dynamic optical localization through available anchor-node selection has been investigated in [22].

Therefore, for detectable optical observations, the local approximations (ϕ≈0) and (ψ≈0) are adopted to derive a tractable RSS-to-distance relationship. If motion or orientation changes interrupt the LoS link, Equation (14) is not applied to that ranging interval. Optical link availability estimation and alignment tracking are beyond the scope of this study. Under this conditional LoS assumption, the received optical power (*P_rn_*) is simplified as:

(14) $$P_{rx}\left(d_{ij}\right)=P_{tx}\eta_{opt}\frac{\left(m+1\right)A_{rx}}{2\pi d_{ij}^{2}}\exp\left(-c\left(\lambda ,J\right)d_{ij}\right)$$

Let the lumped parameter be $C_{sys}=P_{tx}\eta_{opt}\frac{\left(m+1\right)A_{rx}}{2\pi }$. The standard form of the Lambert W function can then be expressed as:

(15) $$\frac{c\left(\lambda ,J\right)d_{ij}}{2}\cdot \exp\left(\frac{c\left(\lambda ,J\right)d_{ij}}{2}\right)=\frac{c\left(\lambda ,J\right)}{2}\sqrt{\frac{C_{sys}}{P_{rx}}}$$

In practical RSS-based optical ranging, the received optical power is affected by random perturbations introduced by the photodetector and receiver circuits. Therefore, the noise is first introduced in the received-power domain rather than being directly added to the distance obtained after nonlinear inversion. Based on the noise-free received optical power $P_{rn}\left(d_{ij}\right)$ defined in Equation (14), the measured received optical power is expressed as:

(16a) $${\tilde{P}}_{rn}=P_{rn}\left(d_{ij}\right)+n_{\mathrm{RSS}},\;E\left[n_{\mathrm{RSS}}\right]=0,\;\mathrm{Var}\left(n_{\mathrm{RSS}}\right)=\sigma_{\mathrm{RSS}}^{2}$$

where ${\tilde{P}}_{rn}$ denotes the measured received optical power, $n_{\mathrm{RSS}}$ denotes the random perturbation in the received-power domain, and $\sigma_{\mathrm{RSS}}^{2}$ denotes its variance.

For compactness, the nonlinear inverse mapping from received optical power to propagation distance is defined as:

(16b) $$g\left(P\right)=\frac{2}{c\left(\lambda ,J\right)}W_{0}\left[\frac{c\left(\lambda ,J\right)}{2}\sqrt{\frac{C_{sys}}{P_{rn}\left(d_{ij}\right)}}\right]$$

where $W_{0}\left(\cdot \right)$ denotes the principal branch of the Lambert W function. By applying this nonlinear inverse mapping to the measured received power, the optical observation distance is obtained as:

(16c) $$z_{op}=g\left({\tilde{P}}_{rn}\right)=\frac{2}{c\left(\lambda ,J\right)}W_{0}\left[\frac{c\left(\lambda ,J\right)}{2}\sqrt{\frac{C_{sys}}{{\tilde{P}}_{rn}}}\right],\;g\left(P_{rn}\left(d_{ij}\right)\right)=d_{ij}$$

Equation (15) transforms the nonlinear received-power equation (Equation (14)) into the standard form uexp(u) = q, where u = c(λ, J)*d*_ij_/2, thereby enabling a closed-form solution through the Lambert *W* function [41]. Under the noise-free propagation model, $g\left(P_{rn}\left(d_{ij}\right)\right)=d_{ij}$, whereas the actual optical observation $z_{op}=g\left({\tilde{P}}_{rn}\right)$ remains affected by the received-power perturbation $n_{RSS}$.

It should be emphasized that the Lambert *W*-based inversion does not eliminate the nonlinearity of the optical ranging process. Instead, $z_{op}$ remains a nonlinear function of the measured received optical power ${\tilde{P}}_{rn}$. Since the left-hand side of Equation (15) is monotonically increasing for $d_{ij}\geq 0$, the principal branch $W_{0}\left(\cdot \right)$ provides a unique nonnegative physical solution. The influence of $n_{RSS}$ on the distance-domain observation is therefore propagated through the nonlinear function $g\left(\cdot \right)$ before heterogeneous ranging fusion is performed.

## 4. A High-Precision Quantification Model Considering Adaptive Robust Fusion Under Underwater Environmental Uncertainty

Complex underwater physical environments induce a substantial number of NLOS errors in acoustic and optical ranging, which are difficult to quantify accurately and make multi-source heterogeneous networks highly susceptible to fluctuations caused by time-varying environmental interference. To address this issue, a high-precision quantification model with adaptive robust fusion is proposed in this paper. This model is primarily divided into two core sub-modules. The first is the environmental uncertainty modeling and ranging variance characterization sub-module. By deeply integrating the Wenz environmental noise model with the Haltrin bio-optical model, the dynamic distribution of underwater time-varying noise is accurately characterized by this sub-module; thereby, the physical isolation of adverse observation data and the high-precision quantitative characterization of the heterogeneous ranging error variance are achieved. The second is the adaptive weighted fusion sub-module based on MVUE. By utilizing the perceived ranging variance as a dynamic feedback input, the confidence weights of the acoustic and optical links are calculated and adjusted in real time by this sub-module, whereby the efficient merging of multi-modal observation data is executed. The overall fusion model therefore exhibits strong robustness and high environmental adaptability. The transmission path of extreme long-tail noise toward the localization calculation can be intelligently blocked from the underlying physical mechanism, and efficient robust fusion under complex and variable hydrological conditions is realized. Consequently, a highly reliable distance input benchmark is provided for the subsequent accurate optimization of the 3D coordinates of the target node.

**Figure 2:**
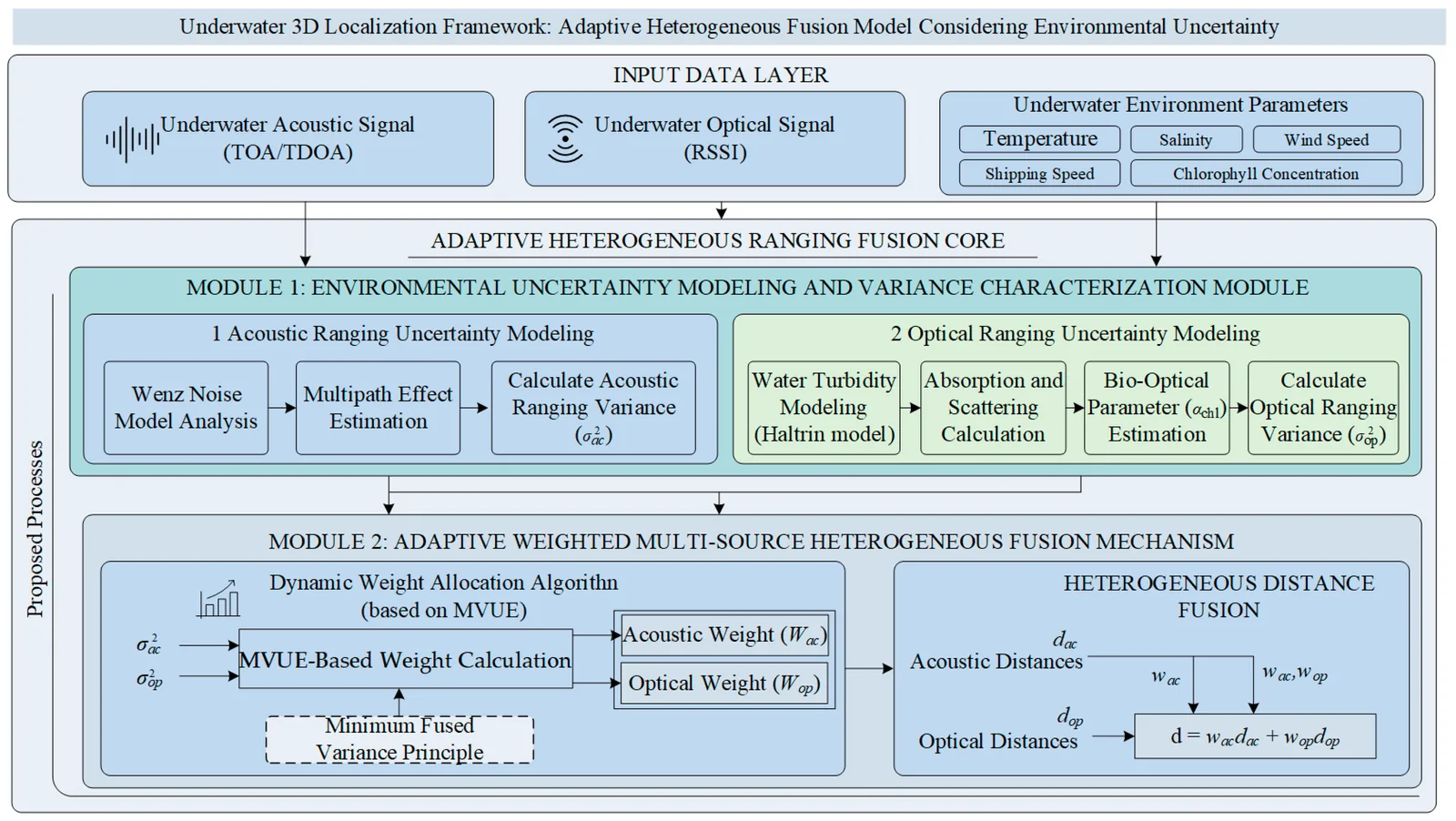
Overall architecture diagram of the adaptive fusion model.

To clearly demonstrate the logical interactions and data flows among the aforementioned core modules, the overall architecture of the adaptive fusion model proposed in this section is illustrated in Figure 2. The complete processing pipeline, ranging from the perception of physical environmental parameters and the quantitative characterization of error variance to the final completion of the robust fusion of multi-source heterogeneous distances, is intuitively presented in this figure.

### 4.1. Heterogeneous Error Ranging Model Under Underwater Environmental Uncertainty

Achieving high-precision localization in complex and dynamic underwater environments is difficult when relying directly on raw acoustic or optical observations. This is because acoustic ranging provides wide coverage but is highly susceptible to sea-surface wind and wave noise as well as sound-speed-profile bending, which induce large measurement variances and systematic biases. Meanwhile, optical ranging provides high precision at short distances but is extremely sensitive to water turbidity (such as chlorophyll concentration), leading to rapid attenuation. Single-modal observations therefore exhibit significant time-varying characteristics and environmental uncertainties. Therefore, measurement values cannot be simply averaged; instead, an environment-driven error analytical model must first be established to quantify the ranging error variance under different environmental parameters. On this basis, utilizing the complementary characteristics of acoustic and optical media, an adaptive weighted fusion model $d_{fuse}=\alpha z_{ac}+\beta z_{op}$ is constructed. By dynamically adjusting the weight coefficients, ranging uncertainty is minimized in a statistical sense, whereby an optimal estimation approaching the true physical distance is obtained.

The environmental parameters used in the uncertainty model are treated as externally measured or estimated environmental-state inputs rather than variables inferred by the proposed localization algorithm. In practice, the sea-surface wind speed can be obtained from a surface buoy or a nearby meteorological station [3], while the shipping activity factor can be estimated from regional vessel-traffic information and normalized to [0, 1] [2]. The sound-speed-profile gradient can be calculated from measured sound-speed–depth data [4], and the chlorophyll concentration can be obtained from in situ optical sensors or ocean-color observations [5]. In the simulations, representative values of these parameters are prescribed according to [2,3,4,5].

It should be clarified that the inverse-variance fusion is not directly performed on the raw biased measurements. Before fusion, the deterministic sound-speed-profile bending bias in the acoustic range is compensated using the ISSP-based correction model, while the deterministic optical attenuation effect is incorporated into the Haltrin-based RSS distance inversion. Therefore, the acoustic and optical quantities entering the fusion stage are bias-compensated ranging observations, and only their remaining environment-dependent residual errors are modeled as zero-mean random variables. For clarity, the definitions of the key symbols used in Section 4 and Section 5 are summarized in Appendix A.

#### 4.1.1. Acoustic Uncertainty Modeling

Let $z_{ac}^{raw}$ denote the raw acoustic ranging result and ${\overset{\wedge }{b}}_{ssp}$ denote the deterministic bending-bias estimate obtained from the ISSP-based correction model. The bias-compensated acoustic range is defined as $z_{ac}=z_{ac}^{raw}-{\overset{\wedge }{b}}_{ssp}$. The acoustic observation entering the fusion stage is then modeled as:

(17) $${\tilde{z}}_{ac}=z_{ac}+\xi_{ac},\;\xi_{ac}∼N\left(0,\sigma_{ac}^{2}\right)$$

where $\xi_{ac}$ denotes the remaining random ranging error after the model-based bias compensation and is approximated as a zero-mean Gaussian variable. Its variance $\xi_{ac}$ is environment-dependent and characterizes the residual acoustic ranging uncertainty under the current underwater conditions. According to the physical characteristics of underwater acoustics, the derivation process of this variance is as follows:

1. Environmental Noise Level Modeling

To connect the environmental noise model with the matched-filter CRLB in Equation (10b), the Wenz noise level must first be converted from the logarithmic power spectral density to a linear effective noise spectral density over the LFM bandwidth. Let $N_{\mathrm{ref}}$ denote the acoustic reference spectral density. The linear noise spectral density and its bandwidth-averaged value are given by:

(18a) $$N_{0}\left(f\right)=N_{\mathrm{ref}}10^{N_{L}\left(f\right)/10},\;N_{0}^{\mathrm{eff}}\left(f_{c},B\right)=\frac{1}{B}\int_{f_{c}-B/2}^{f_{c}+B/2}N_{0}\left(f\right)df$$

After the transmission loss is converted to the linear domain, the received acoustic power and the matched-filter energy SNR are expressed as:

(18b) $$P_{r}\left(d_{ij}\right)=P_{t}d_{ij}^{-k}10^{-\alpha_{\mathrm{abs}}\left(f_{c}\right)d_{ij,\mathrm{km}}/10},\;\Gamma_{\mathrm{MF},ij}=\frac{P_{r}\left(d_{ij}\right)T_{s}}{N_{0}^{\mathrm{eff}}\left(f_{c},B\right)}$$

where $P_{t}$ and $P_{r}$ are expressed using the same acoustic power or pressure-squared reference, $T_{s}$ is the LFM pulse duration in seconds, *B* is the signal bandwidth in Hz, $d_{ij,km}=d_{ij}/1000$ is the propagation distance in kilometers, $\alpha_{abs}$ is measured in dB/km, and $\Gamma_{\mathrm{MF},ij}$ is dimensionless. The absorption coefficient $\alpha_{abs}\left(f_{c}\right)$ and the environmental noise level $NL\left(f\right)$ involved in Equation (18a,b) are further specified below.

First, the acoustic absorption coefficient $\alpha_{abs}\left(f_{c}\right)$ used in the transmission-loss term of Equation (18b) is calculated using the classical Thorp empirical formula:

(19) $$\alpha_{abs}\left(f\right)=\frac{0.11f^{2}}{1+f^{2}}+\frac{44f^{2}}{4100+f^{2}}+2.75\times 10^{-4}f^{2}+0.003$$

where f is expressed in kHz and $\alpha_{abs}\left(f\right)$ is expressed in dB/km. In Equation (18b), $\alpha_{abs}\left(f_{c}\right)$ is evaluated at the acoustic carrier frequency $f_{c}$.

Second, the environmental noise level $NL\left(f\right)$ appearing in Equation (18a) is calculated using the Wenz model [42]. The total one-sided noise power spectral density is obtained by linearly superimposing the turbulence, shipping, wind-driven, and thermal noise components:

(20) $$10^{\frac{NL\left(f\right)}{10}}=10^{\frac{NL_{turb}\left(f\right)}{10}}+10^{\frac{NL_{ship}\left(f\right)}{10}}+10^{\frac{NL_{wind}\left(f\right)}{10}}+10^{\frac{NL_{th}\left(f\right)}{10}}$$

The physical parameterization formulas for each component are as follows:

Turbulence Noise (1 Hz < f < 10 Hz):

(21) $$NL_{turb}\left(f\right)=17-30\log_{10}\left(f\right)$$

Shipping Noise (10 Hz < f < 100 Hz):

The shipping factor $s\in \left[0,1\right]$ (where 0 represents low shipping and 1 represents high shipping) is introduced:

(22) $$NL_{ship}\left(f\right)=40+20\left(s-0.5\right)+26\log_{10}\left(f\right)-60\log_{10}\left(f+0.03\right)$$

Wind-Driven Noise (100 Hz < f < 25 kHz):

The sea surface wind speed $v_{wind}$ is introduced:

(23) $$NL_{wind}\left(f\right)=50+7.5\sqrt{v_{wind}}+20\log_{10}\left(f\right)-40\log_{10}\left(f+0.4\right)$$

Thermal Noise (f > 100 kHz):

(24) $$NL_{th}\left(f\right)=-15+20\log_{10}\left(f\right)$$

Synthesizing the aforementioned four items, the complete analytical expression of the total environmental Noise Level $NL\left(f\right)$ is given by:

(25) $$NL\left(f\right)=10\log_{10}\left(10^{\frac{17-30\mathrm{lg}f}{10}}+10^{\frac{40+20\left(s-0.5\right)+26\mathrm{lg}f-60\mathrm{lg}\left(f+0.03\right)}{10}}+10^{\frac{50+7.5\sqrt{v}+20\mathrm{lg}f-40\mathrm{lg}\left(f+0.4\right)}{10}}+10^{\frac{-15+20\mathrm{lg}f}{10}}\right)$$

Equation (25) yields the total frequency-dependent one-sided environmental noise level $NL\left(f\right)$ by summing the turbulence, shipping, wind-driven, and thermal noise components in the linear power domain and then converting the result back to the logarithmic domain. Therefore, the dominant noise source varies with the operating frequency and environmental conditions. In particular, increases in shipping activity or sea-surface wind speed raise the total noise level within the corresponding frequency band. The resulting $NL\left(f\right)$ is subsequently converted into the effective linear noise spectral density in Equation (18a), which reduces the matched-filter SNR in Equation (18b) and ultimately increases the acoustic ranging variance in Equation (26). Thus, Equation (25) establishes the quantitative link between the measured underwater environment and acoustic ranging uncertainty.

2. Stratification Effect and Acoustic Ray Bending Correction

The underwater sound speed is not constant but varies with depth according to an Isogradient Sound Speed Profile (ISSP). This stratification bends the acoustic propagation path, thereby inducing a systematic bias in ranging based on the straight-line propagation assumption. The deterministic component of the acoustic ray-bending error is first compensated for using the estimated sound-speed-profile gradient. However, because the sound-speed gradient and propagation path cannot be estimated perfectly, a residual correction error may remain. This residual component is treated as a zero-mean model-mismatch error whose variance increases with the depth difference, propagation distance, and uncertainty of the sound-speed-profile estimate.

3. Joint Error Variance Model

Accordingly, the effective acoustic ranging variance is formulated as a noise-stratification jointly driven residual uncertainty model:

(26) $$\sigma_{ac}^{2}\approx \underset{\underset{\mathrm{SNR-Dependent}\;\mathrm{Jitter}}{︸}}{K_{noise}\left(v_{wind},s\right)\cdot d_{ij}}+\underset{\underset{\mathrm{Stratification-Induced}\;\mathrm{Bias}}{︸}}{K_{bend}\left(\Delta z_{ij},g_{ssp}\right)\cdot d_{ij}^{2}}+\sigma_{ac,0}^{2}$$

where $K_{noise}\left(v_{wind},s\right)$ is the environmental noise degradation factor, satisfying $K_{noise}\left(v_{wind},s\right)\propto N_{ship}\left(f,s\right)+N_{wind}\left(f,v_{wind}\right)$. It increases with increasing sea-surface wind speed $v_{wind}$ and shipping density s, reflecting the increased ranging uncertainty caused by the reduced SNR. $K_{bend}\left(\Delta z_{ij},g_{ssp}\right)$ is the residual ray-bending uncertainty factor after the model-based SSP correction; $g_{ssp}$ is the sound speed profile gradient (with a typical value of $0.017\;s^{-1}$). This term characterizes the uncertainty caused by imperfect SSP compensation, indicating that the residual ranging variance increases when the depth difference or propagation distance becomes large. It should be emphasized that this term represents the variance of the remaining correction mismatch rather than an uncompensated deterministic bias. $\sigma_{ac,0}^{2}$ is the system acoustic background noise variance. The distance-independent hardware inherent errors (such as clock synchronization jitter) are characterized by it.

#### 4.1.2. Optical Ranging Error

According to Equation (16a–c), the optical observation $z_{op}$ is obtained by applying the nonlinear inverse mapping $g\left(\cdot \right)$ to the noisy received-power measurement ${\tilde{P}}_{rn}=P_{rn}\left(d_{ij}\right)+n_{\mathrm{RSS}}$. Therefore, the received-power uncertainty must be propagated through the nonlinear Lambert *W*-based inversion before an equivalent distance-domain error model can be established.

When the received-power perturbation is sufficiently small relative to the noise-free received power, i.e., $\left|n_{\mathrm{RSS}}\right|<P_{rn}\left(d_{ij}\right)$, a second-order Taylor expansion of $g\left({\tilde{P}}_{rn}\right)$ around the noise-free operating point $P_{rn}\left(d_{ij}\right)$ gives [43]:

(27a) $$\begin{array}{cc} z_{op} & =g\left(P_{rn}\left(d_{ij}\right)+n_{\mathrm{RSS}}\right)\approx g\left(P_{rn}\left(d_{ij}\right)\right)+g^{\prime }\left(P_{rn}\left(d_{ij}\right)\right)n_{\mathrm{RSS}}+\frac{1}{2}g^{\prime \prime }\left(P_{rn}\left(d_{ij}\right)\right)n_{\mathrm{RSS}}^{2}\\ & =d_{ij}+g^{\prime }\left(P_{rn}\left(d_{ij}\right)\right)n_{\mathrm{RSS}}+\frac{1}{2}g^{\prime \prime }\left(P_{rn}\left(d_{ij}\right)\right)n_{\mathrm{RSS}}^{2} \end{array}$$

For compactness, the argument of the Lambert *W* function is denoted by:

(27b) $$q\left(P\right)=\frac{c\left(\lambda ,J\right)}{2}\sqrt{\frac{C_{\mathrm{sys}}}{P}}$$

Using the derivative property of the Lambert *W* function, the first derivative of the nonlinear inverse mapping in Equation (16b) is obtained as:

(27c) $$g^{\prime }\left(P\right)=-\frac{W_{0}\left(q\left(P\right)\right)}{c\left(\lambda ,J\right)P\left[1+W_{0}\left(q\left(P\right)\right)\right]}$$

At the noise-free operating point $P=P_{rn}\left(d_{ij}\right)$, the inverse relationship between Equations (14) and (16c) gives:

(27d) $$\begin{array}{c} W_{0}\left(q\left(P_{rn}\left(d_{ij}\right)\right)\right)=\frac{c\left(\lambda ,J\right)d_{ij}}{2},\\ g^{\prime }\left(P_{rn}\left(d_{ij}\right)\right)=-\frac{d_{ij}}{P_{rn}\left(d_{ij}\right)\left[2+c\left(\lambda ,J\right)d_{ij}\right]} \end{array}$$

The second derivative of the nonlinear inverse mapping is:

(27e) $$g^{\prime \prime }\left(P\right)=\frac{W_{0}\left(q\left(P\right)\right)\left[2W_{0}^{2}\left(q\left(P\right)\right)+4W_{0}\left(q\left(P\right)\right)+3\right]}{2c\left(\lambda ,J\right)P^{2}{\left[1+W_{0}\left(q\left(P\right)\right)\right]}^{3}}$$

Because $E\left[n_{\mathrm{RSS}}\right]=0$, the first-order term in Equation (27a) has zero mean. However, since $g^{\prime \prime }\left(P\right)\neq 0$, the second-order term introduces a nonlinear inversion bias. Under the second-order delta-method approximation, this bias is expressed as:

(27f) $$b_{op}\approx \frac{1}{2}g^{\prime \prime }\left(P_{rn}\left(d_{ij}\right)\right)\sigma_{\mathrm{RSS}}^{2}$$

Such a bias is a general consequence of nonlinear RSS-to-distance inversion and has also been reported in RSS-based optical distance estimation [44]. Accordingly, the optical observation entering the heterogeneous fusion stage is corrected by subtracting the second-order inversion bias. The bias-corrected optical observation and its locally equivalent residual error are expressed as:

(27g) $$\begin{array}{c} {\tilde{z}}_{op}=z_{op}-b_{op}=d_{ij}+\xi_{op}\\ \xi_{op}\approx g^{\prime }\left(P_{rn}\left(d_{ij}\right)\right)n_{\mathrm{RSS}}+\frac{1}{2}g^{\prime \prime }\left(P_{rn}\left(d_{ij}\right)\right)\left(n_{\mathrm{RSS}}^{2}-\sigma_{\mathrm{RSS}}^{2}\right)\\ E\left[\xi_{op}\left|e\right.\right]\approx 0 \end{array}$$

where *e* denotes the measured environmental state. Equation (27g) shows that the second-order mean component has been removed, while the remaining random component is retained in the equivalent distance-domain residual. Under the adopted small-noise approximation, the first-order term dominates the residual variance, while the contribution of order $O\left(\sigma_{\mathrm{RSS}}^{4}\right)$ is neglected. Therefore, the residual is approximated by a zero-mean Gaussian variable with the environment-dependent variance:

(27h) $$\begin{array}{c} \xi_{op}\overset{\mathrm{approx}}{∼}N\left(0,\sigma_{op}^{2}\right),\\ \sigma_{op}^{2}=Var\left(\xi_{op}\left|e\right.\right)\approx \approx {\left[g^{\prime }\left(P_{rn}\left(d_{ij}\right)\right)\right]}^{2}\sigma_{\mathrm{RSS}}^{2}+\sigma_{op,0}^{2}\\ . \end{array}$$

where $\sigma_{op}^{2}$ denotes the optical-system intrinsic variance independent of the received signal strength, as further specified in Equation (36). Therefore, Equation (27a–h) do not re-declare the nonlinear optical channel as a linear propagation model. Instead, they establish a locally equivalent and approximately conditionally unbiased distance-domain observation after the received-power uncertainty has been propagated through the nonlinear Lambert *W*-based inverse function and the second-order inversion bias has been removed.

The signal-dependent component of $\sigma_{op}^{2}$ is subsequently parameterized according to the photodetector noise, received optical power, and inherent bio-optical properties of the water body. The detailed derivation is given below.

For the optical link, the shot noise of the photodetector is considered the principal signal-dependent error source. According to quantum detection theory and the local uncertainty-propagation relationship established in Equation (27a–h), the received-power-dependent component of the equivalent optical ranging variance is expressed as:

(28) $$\sigma_{op}^{2}=\frac{\hbar \nu B_{op}F}{2\cdot P_{rx}\left(d_{ij}\right)}+\sigma_{dark}^{2}$$

where ℏ is the Planck constant, taking the value of $6.626\times 10^{-34}\;J\cdot s$, and v is the optical frequency, formulated as ν=clightλ is the speed of light in a vacuum. $B_{op}$ is the electrical bandwidth of the optical receiver, by which the range of noise integration is determined. F is the noise figure of the photodiode, describing the additional noise introduced by the device. $P_{rx}\left(d_{ij}\right)$ is the received optical power, the value of which is determined by the attenuation coefficient of the water body $c\left(\lambda \right)$. $\sigma_{dark}^{2}$ is the dark current variance, representing the system background noise that is independent of illumination.

To endow $c\left(\lambda ,J\right)$ with a clear physical meaning, the Haltrin bio-optical model is adopted [45], and it is decomposed into the absorption coefficient $a\left(\lambda \right)$ and the scattering coefficient $a\left(\lambda \right)$:

(29) $$c\left(\lambda \right)=a\left(\lambda \right)+b\left(\lambda \right)$$

1. Absorption Model

The total absorption coefficient is composed of pure seawater, phytoplankton (chlorophyll), and colored dissolved organic matter:

(30) $$a\left(\lambda \right)=a_{w}\left(\lambda \right)+a_{chl}^{0}\left(\lambda \right)\cdot {\left[C_{chl}\right]}^{e\left(\lambda \right)}+a_{CDOM}\left(\lambda \right)$$

where $C_{chl}$ is the chlorophyll concentration (mg/m^3^), which is considered the core indicator for distinguishing Jerlov water types [46]. $a_{CDOM}\left(\lambda \right)$ exhibits exponential decay with wavelength:

(31) $$a_{CDOM}\left(\lambda \right)=a_{CDOM}\left(440\right)\exp\left(-S_{dom}\left(\lambda -440\right)\right)$$

2. Scattering Model

The total scattering coefficient is composed of pure seawater scattering and suspended particle scattering:

(32) $$b\left(\lambda \right)=b_{w}\left(\lambda \right)+b_{p}\left(\lambda \right)$$

An empirical relationship between the particle scattering coefficient $b_{p}\left(\lambda \right)$ and the chlorophyll concentration $C_{chl}$ (Gordon–Morel model) is formulated as:

(33) $$b_{p}\left(\lambda \right)=0.30\cdot {\left(C_{chl}\right)}^{0.62}\cdot \left(\frac{550}{\lambda }\right)$$

3. Mapping between Jerlov Water Types and $C_{chl}$

Different chlorophyll concentration ranges correspond to different Jerlov types *J*. For example:

(34) $$C_{chl}\left(J\right)=\left\{\begin{array}{ll} 0.03\;\mathrm{mg}/m^{3}, & J=\mathrm{Type}\;I\\ 2.00\;\mathrm{mg}/m^{3}, & J=\mathrm{Type}\;\mathrm{III}\\ 10.0\;\mathrm{mg}/m^{3}, & J=\mathrm{Coastal}\;1 \end{array}\right.$$

Synthesizing Equations (30) to (34), the complete parameterized formula for the beam attenuation coefficient $c\left(\lambda ,C_{chl}\right)$ is given by:

(35) $$c\left(\lambda ,C_{chl}\right)=a_{w}\left(\lambda \right)+a_{chl}^{0}\left(\lambda \right){\left[C_{chl}\right]}^{e}+a_{CDOM}\left(440\right)e^{-S\left(\lambda -440\right)}+b_{w}\left(\lambda \right)+0.3{\left(C_{chl}\right)}^{0.62}\left(\frac{550}{\lambda }\right)$$

By substituting Equation (35) into Equation (28) and incorporating the local nonlinear-inversion sensitivity in Equation (27c,d), the received-power-dependent terms are collected into the environment-dependent coefficient $K_{op}\left(C_{chl}\right)$. The final bio-optical-dependent variance model is therefore obtained as:

(36) $$\sigma_{op}^{2}\approx K_{op}\left(C_{chl}\right)\cdot d_{ij}+\sigma_{op,0}^{2}$$

This model indicates that the optical ranging uncertainty, represented by $K_{op}$, is directly determined by the concentration of biochemical components $C_{chl}$ in the water body. $\sigma_{op,0}^{2}$ denotes the intrinsic variance of the optical system. The signal-strength-independent noise components of the optical receiver are mainly characterized by this term, including the dark-current noise and thermal noise in Equation (28): $\sigma_{op,0}^{2}=\sigma_{dark}^{2}+\sigma_{thermal}^{2}$, where $\sigma_{dark}^{2}=2qI_{dark}B_{op}F$ represents the shot-noise variance associated with the dark current of the photodiode ($I_{dark}$ is the dark current and q is the electron charge), and $\sigma_{thermal}^{2}=4k_{B}T_{sys}B_{op}/R_{L}$ represents the thermal-noise variance generated by the load resistor $R_{L}$ ($k_{B}$ is the Boltzmann constant and $T_{sys}$ is the system temperature).

### 4.2. Adaptive Linear Weighted Fusion Mechanism

#### 4.2.1. Linear Fusion Model

After the bias-compensated acoustic observation ${\tilde{z}}_{ac}$ and the nonlinear-inversion-bias-corrected optical observation ${\tilde{z}}_{op}$ are obtained, they are linearly fused to produce a more reliable distance estimate $d_{fuse}$:

(37) $$d_{fuse}=\lambda_{1}{\tilde{z}}_{ac}+\lambda_{2}{\tilde{z}}_{op}$$

The fusion is performed in the distance domain after the deterministic acoustic ray-bending bias and the second-order optical inversion bias have been compensated for. Conditioned on the measured environmental state e, the acoustic residual error is modeled as zero mean, while the optical residual error is approximately zero mean according to the local nonlinear uncertainty-propagation model in Equation (27a–h). Therefore, the conditional expectation of Equation (37) is

(38) $$E\left[d_{fuse}\left|e\right.\right]=\lambda_{1}E\left[{\tilde{z}}_{ac}\left|e\right.\right]+\lambda_{2}E\left[{\tilde{z}}_{op}\left|e\right.\right]\approx \left(\lambda_{1}+\lambda_{2}\right)d_{\mathrm{real}}$$

where $d_{\mathrm{real}}=d_{\mathrm{ij}}$ for the considered acoustic–optical link. To satisfy the conditional local unbiasedness requirement $E\left[d_{fuse}\left|e\right.\right]\approx d_{\mathrm{real}}$, the weight coefficients must satisfy the normalization constraint $\lambda_{1}+\lambda_{2}=1$.

Equation (39) is therefore imposed on the post-compensation and locally equivalent distance-domain observations rather than directly on the nonlinear received-power measurements. The approximation in Equation (38) arises from the higher-order terms neglected in the local Taylor expansion and does not imply that the nonlinear relationship in Equations (14)–(16c) has been discarded.

#### 4.2.2. Derivation of Optimal Weight Coefficients

The following inverse-variance derivation is conditioned on the measured environmental state e, including the sea-surface wind speed, shipping activity, chlorophyll concentration, and sound-speed-profile gradient. After the deterministic acoustic ray-bending bias has been compensated and the second-order nonlinear inversion bias of the optical observation has been removed, the remaining acoustic and optical distance-domain errors are denoted by $\xi_{ac}$ and $\xi_{op}$, respectively. They are assumed to satisfy

(39) $$E\left[\xi_{ac}\left|e\right.\right]=0,\;E\left[\xi_{op}\left|e\right.\right]\approx 0,\;\mathrm{Cov}\left(\xi_{ac},\xi_{op}\left|e\right.\right)=0$$

The first two conditions represent the conditional unbiasedness of the acoustic observation and local conditional unbiasedness of the optical observation, respectively. The covariance condition is adopted because the acoustic and optical residual errors arise from different propagation media, ranging mechanisms, and receiver noise sources. Therefore, the original optical power channel is not assumed to be globally linear, nor is its nonlinear inverse assumed to be exactly unbiased. Instead, the minimum-variance weights are derived for the post-inversion and bias-corrected distance-domain observations.

Under the above conditional local unbiasedness and independence assumptions, the variance of the fused estimation error is obtained according to the law of error propagation as

(40) $$\sigma_{\mathrm{fuse}}^{2}=\lambda_{1}^{2}\sigma_{ac}^{2}+\lambda_{2}^{2}\sigma_{op}^{2}$$

The optimization objective is to determine the weight coefficient $\lambda_{1}$ that minimizes $\sigma_{\mathrm{fuse}}^{2}$. By substituting the normalization constraint $\lambda_{2}=1-\lambda_{1}$ into Equation (40) and taking the first-order derivative with respect to $\lambda_{1}$, it follows that

(41) $$\frac{\partial \left(\sigma_{\mathrm{fuse}}^{2}\right)}{\partial \lambda_{1}}=2\lambda_{1}\sigma_{ac}^{2}-2\left(1-\lambda_{1}\right)\sigma_{op}^{2}=0$$

By solving Equation (41), the conditional minimum-variance weight coefficients are obtained as

(42) $$\lambda_{1}=\frac{\sigma_{op}^{2}}{\sigma_{ac}^{2}+\sigma_{op}^{2}}$$

(43) $$\lambda_{1}=\frac{\sigma_{ac}^{2}}{\sigma_{ac}^{2}+\sigma_{op}^{2}}$$

Within the adopted local small-noise approximation, Equations (42) and (43) constitute a conditional local MVUE solution for the two bias-corrected distance-domain observations. The acoustic ranging variance $\sigma_{ac}^{2}$ is directly obtained from Equation (26), whereas the optical ranging variance $\sigma_{op}^{2}$ is obtained from the nonlinear uncertainty propagation in Equation (27a–h) and the bio-optical parameterization in Equation (36). Therefore, the nonlinearity of the optical RSS inversion is retained through its contribution to $\sigma_{op}^{2}$ and is consequently reflected in the adaptive optical weight $\lambda_{2}$.

When the sea-surface wind speed is extremely high and the water is relatively clear, the acoustic ranging variance increases, resulting in $\lambda_{1}\to 0$ and $\lambda_{2}\to 1$; therefore, the fused distance mainly relies on the optical observation. Conversely, when the water is highly turbid and the sea state is calm, the optical ranging variance increases, resulting in $\lambda_{1}\to 1$ and $\lambda_{2}\to 0$; therefore, the fused distance mainly relies on the acoustic observation. These results indicate that the weight coefficients remain functions of environmental parameters, including wind speed, turbidity, and depth difference.

Residual NLOS outliers or model-mismatch errors that cannot be fully removed by the physical compensation and local bias-correction processes are not assumed to satisfy the above conditional local unbiasedness conditions. Their influence is further suppressed by the generalized Huber loss in Equation (67) during the subsequent coordinate-optimization stage.

## 5. 3D Coordinate Optimization Framework Based on GP-AC Algorithm

**Figure 3:**
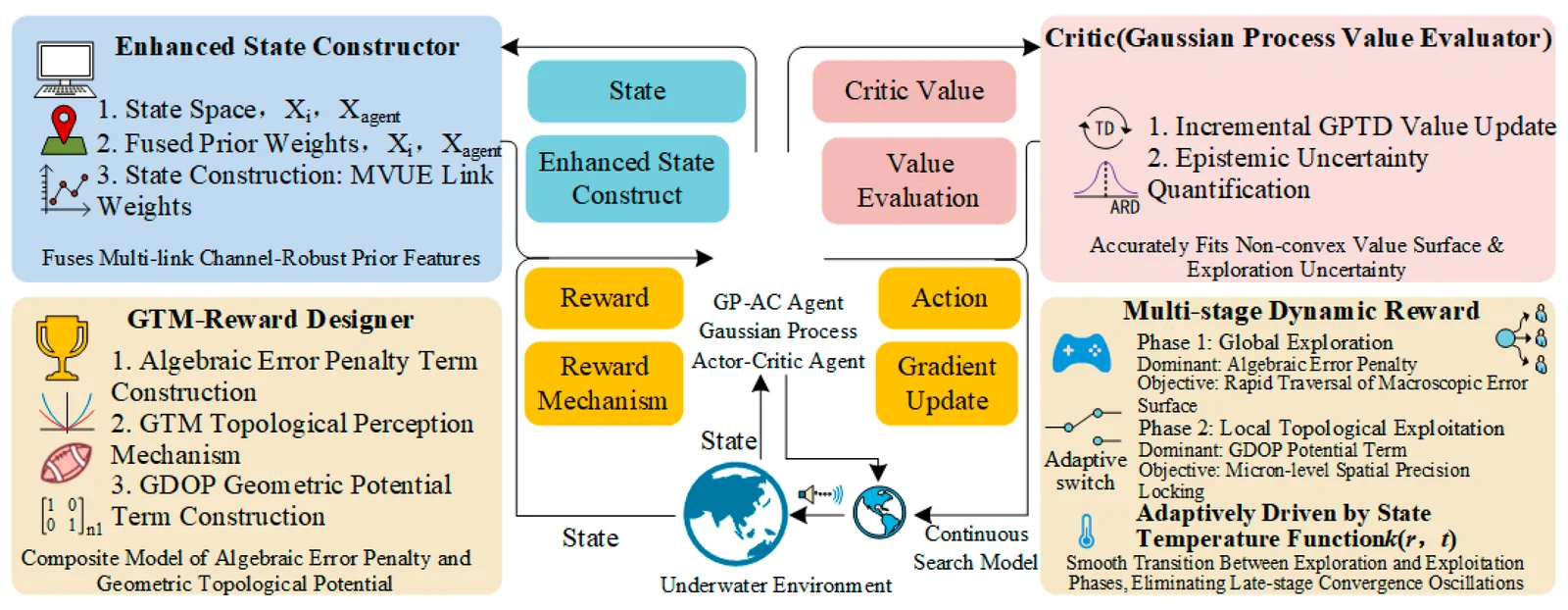
Overall framework of the proposed algorithm. The arrows indicate the direction of information flow and interactions among the functional modules, while the lines with different colors are used only for visual distinction and do not represent additional physical quantities or algorithmic categories.

Complex deep-water environments expose acoustic and optical ranging links to severe dynamic disturbances, such as sea-surface wind and water turbidity, resulting in difficult-to-quantify ranging uncertainty. Moreover, traditional optimization algorithms may become trapped in local minima or exhibit degraded performance under ill-conditioned anchor geometries when solving highly non-convex three-dimensional localization problems. To address these challenges, a geometric topology-perception-enhanced multi-stage reinforcement learning framework, termed GP-AC, is proposed for node localization in underwater heterogeneous networks. Although recursive GPTD updating, NPG optimization, and GDOP evaluation introduce moderate per-step computations, GP-AC requires no offline training dataset and reduces online computational requirements through recursive learning from observed transitions, single-agent sequential decision-making, and multi-stage guidance that avoids excessive blind exploration. Figure 3 presents the overall architecture and information flow of the proposed GP-AC framework, while its detailed step-by-step implementation is provided in Algorithm 1.

### 5.1. Problem Description

In Section 4, by analyzing the physical characteristics of underwater heterogeneous acoustic and optical communications, an environment-driven error analytical model was established, and the weighted fused observation distance $d_{fuse}$ between the unknown node to be localized and the anchor node, along with the corresponding total ranging error variance $\sigma_{total}^{2}$, was obtained utilizing the MVUE criterion. However, only scalar distance information is represented by $d_{fuse}$, and the 3D spatial coordinates of the node are not directly provided.

The essence of the precise localization problem is considered to be the process of inversely solving the coordinates of the unknown node by utilizing the coordinate information of known anchor nodes and the observation distance information obtained from Section 4. Let the true coordinate vector of the unknown node to be localized in the 3D underwater space be denoted as X:

(44) $$X={\left[x,y,z\right]}^{T}$$

Meanwhile, assuming that N heterogeneous anchor nodes with precise known positions are deployed in the network, the coordinate vector of the i−th ($i\in \left\{1,2,\ldots ,N\right\}$) anchor node is denoted as $A_{i}$:

(45) $$A_{i}={\left[x_{i},y_{i},z_{i}\right]}^{T}$$

In an ideal error-free physical space, the a priori true geometric distance $d_{i}^{*}$ between the unknown node X and the i−th anchor node $A_{i}$ can be directly expressed by the vector two-norm:

(46) $$d_{i}^{*}={\left\|X-A_{i}\right\|}_{2}$$

However, due to the high complexity and time-varying interference of the underwater environment, residual errors are inevitably contained in the fused observation distance $d_{fuse,i}$ obtained in Section 4. The underlying spatial observation model at this time can be established as:

(47) $$d_{fuse,i}=d_{i}^{*}+\varepsilon_{i}$$

where $\varepsilon_{i}$ is the residual environmental physical error. According to the environmental variance characterization model established previously, this error can be approximately modeled as following a zero-mean Gaussian distribution, and its degree of uncertainty is strictly bounded by the total variance derived in Section 4:

(48) $$\varepsilon_{i}∼N\left(0,\sigma_{total,i}^{2}\right)$$

The smaller the variance $\sigma_{total,i}^{2}$, the less the ranging value is contaminated by the underlying environment (e.g., sea surface wind speed, water turbidity), and the higher the locational confidence level contained therein.

Due to the objective existence of the error $\varepsilon_{i}$, an independent geometric point capable of perfectly satisfying all ranging Equations simultaneously does not exist in the 3D solution space (i.e., the system of Equations is overdetermined and lacks an exact analytical solution). The spatial ranging residual $e_{i}\left(X\right)$ between the observation distance from an arbitrary candidate coordinate point to the i−th anchor node and the a priori geometric distance is defined as:

(49) $$e_{i}\left(X\right)=d_{fuse,i}-{\left\|X-A_{i}\right\|}_{2}$$

Thus, it can be seen that the localization problem cannot be regarded as a simple rigid body geometric intersection solution; rather, it should be rigorously modeled as a spatial optimization problem. The core task of this section is to transform the a priori information (observation distance $d_{fuse,i}$ and confidence variance $\sigma_{total,i}^{2}$) outputted from Section 4 into a global optimization model, and an optimal coordinate point X is searched for within the feasible solution space, whereby the overall ranging residual of the system is minimized.

### 5.2. Construction of the Optimization Model

#### 5.2.1. Decision Variables and Constraint Conditions

Decision variable: The 3D coordinates of the node to be localized, $X={\left[x,y,z\right]}^{T}$.

Constraint conditions: The node coordinates are required to be located within the physical boundaries of the underwater monitoring area. Assuming that the monitoring area is a rectangular cuboid, its range is determined by $\left[x_{min},x_{max}\right],\left[y_{min},y_{max}\right],\left[z_{min},z_{max}\right]$. In addition, the node depth z must be situated below the sea surface and above the seabed.

(50) $$\left\{\begin{array}{c} x_{min}\leq x\leq x_{max}\\ y_{min}\leq y\leq y_{max}\\ z_{min}\leq z\leq z_{max} \end{array}\right.$$

#### 5.2.2. Construction of the Objective Function

To fully utilize the environmental perception information provided in Section 4, the Weighted Least Squares (WLS) method is adopted to construct the objective function. The calculated Euclidean distance between the coordinates to be solved X and the anchor node i is defined as:

(51) $$d_{calc,i}\left(X\right)=\sqrt{{\left(x-x_{i}\right)}^{2}+{\left(y-y_{i}\right)}^{2}+{\left(z-z_{i}\right)}^{2}}$$

The objective function $f\left(X\right)$ is defined as the sum of weighted squared residuals:

(52) $$\min f\left(X\right)=\sum_{i=1}^{M}\omega_{i}\cdot {\left(d_{calc,i}\left(X\right)-d_{fuse,i}\right)}^{2}$$

The design of the weight coefficient $\omega_{i}$ is considered the link connecting Section 4 and Section 5. According to the principle of Maximum Likelihood Estimation (MLE), the weight should be inversely proportional to the variance of the observation error. Using the total variance $\sigma_{total,i}$ calculated by Equation (40) in Section 4, the weight is defined as

(53) $$\omega_{i}=\frac{1}{\sigma_{total,i}^{2}}$$

The physical significance of this objective function is that, for links with minor environmental interference (e.g., windless or clear water), $\sigma_{total}^{2}$ is relatively small and the weight $\omega_{i}$ is relatively large, and the optimization result will be forced by the algorithm to approach the observation value of this link; conversely, for high-noise links, large fitting residuals are permitted by the algorithm.

### 5.3. A Geometric Topology Perception-Enhanced Multi-Stage Reinforcement Learning Strategy

Aiming at the highly non-convex ranging error surfaces underwater and the problem that traditional optimization algorithms are extremely prone to falling into local minima of ill-conditioned topologies, the GP-AC algorithm is proposed in this section. By introducing Gaussian Process Regression (GPR) and dynamic penalty rewards into the Actor-Critic architecture, environmental cognitive uncertainty is effectively quantified and the agent is guided to circumvent spatial topological traps by this strategy; ultimately, rapid convergence and global high-precision coordinate calculations in complex manifold spaces are achieved.

Although Equation (52) defines a static WLS criterion, the MDP formulation does not alter the original localization objective; instead, it recasts the numerical solution as a sequential coordinate-refinement process, in which the current coordinate and uncertainty-weighted residuals constitute the state, the continuous coordinate increment constitutes the action, and the reductions in the WLS residual and geometric topology penalty constitute the reward. GP-AC is adopted for the specific problem considered here for three reasons. First, standard nonlinear least-squares methods mainly rely on local linearization and may become sensitive to initialization and ill-conditioned anchor geometries, whereas the GP posterior uncertainty provides directed exploration beyond a single local descent trajectory. Second, standard Bayesian optimization can suffer from dimensionality-related sample inefficiency when the search representation includes both coordinates and multi-link residuals, whereas GP-AC uses the GP only for uncertainty-aware value evaluation and employs the Actor to directly generate continuous coordinate updates. Third, the Actor-Critic formulation permits the WLS residual, GDOP, boundary constraints, and search-stage information to be incorporated into a unified adaptive reward, thereby enabling the optimization strategy to respond to time-varying underwater ranging uncertainty [47,48,49].

#### 5.3.1. Construction of Markov Decision Process (MDP) for Localization Problem

To solve the non-convex WLS problem constructed in Section 5.2 in a sequential and uncertainty-aware manner, the iterative coordinate-refinement process is formulated as a continuous-space MDP. This process can be represented by the tuple $M=\langle S,A,P,R,\gamma \rangle$. The core state space S and continuous action space A are first mathematically modeled rigorously in this subsection.

(1) Model Construction of State Space

In the optimization system based on GP-AC, not only does the absolute position of the Agent need to be represented by its state, but a priori observation information of the environment must also be integrated so that inputs with structural characteristics and statistical significance can be provided for the GP. Therefore, the environmental state $s_{t}\in S$ at time t is defined as the cascaded form of the agent’s current 3D coordinate vector and the multi-link weighted ranging residual vector. Its complete mathematical expression is given by:

(54) $$s_{t}={\left[p_{t}^{T},\Psi {\left(p_{t}\right)}^{T}\right]}^{T}={\left[x_{t},y_{t},z_{t},\psi_{1}\left(p_{t}\right),\psi_{2}\left(p_{t}\right),\ldots ,\psi_{N}\left(p_{t}\right)\right]}^{T}\in R^{3+N}$$

To maintain strict consistency with the WLS objective constructed in Section 5.2, the residual-vector component $\psi_{i}\left(p_{t}\right)$ is defined using the MVUE-fused acoustic–optical distance ${\hat{d}}_{fuse,i}$ and the corresponding WLS link weight $\omega_{i}$ defined in Equation (53). The square root of the WLS weight is incorporated into each residual component so that the squared Euclidean norm of the residual vector is exactly consistent with the WLS objective in Equation (52). Its expanded form is:

(55) $$\psi_{i}\left(p_{t}\right)=\sqrt{\omega_{i}}\cdot \left({\hat{d}}_{fuse,i}-\sqrt{{\left(x_{t}-x_{i}^{a}\right)}^{2}+{\left(y_{t}-y_{i}^{a}\right)}^{2}+{\left(z_{t}-z_{i}^{a}\right)}^{2}}\right),\;\forall i\in \left\{1,2,\ldots ,N\right\}$$

In Equation (55), $p_{i}^{a}={\left[x_{i}^{a},y_{i}^{a},z_{i}^{a}\right]}^{T}\in R^{3}$ denotes the known spatial coordinate of the i−th anchor node, and ${\hat{d}}_{fuse,i}$ denotes the MVUE-fused acoustic–optical observation distance between the unknown node and the i−th anchor node obtained in Section 4. This fused observation contains the complementary information of both the bias-compensated acoustic ranging measurement and the nonlinear-inversion-bias-corrected optical ranging measurement, rather than an acoustic-only observation.

The coefficient $\omega_{i}=1/\sigma_{total,i}^{2}$ is the WLS anchor-link weight defined in Equation (53), where $\sigma_{total,i}^{2}$ denotes the total ranging error variance of the i−th fused acoustic–optical link. It should be distinguished from the acoustic and optical modality weights used in the MVUE fusion process. Combining Equations (54) and (55), the squared norm of the residual vector is identical to the fused-distance WLS objective in Equation (52), thereby maintaining consistency between the MDP state representation and the optimization model in Section 5.2.

Considering the 3D deployment boundaries of the underwater sensor network and the maximum ranging capacity of acoustic communication, the complete continuous state space S is strictly constrained within the following bounded closed set:

(56) $$S=\left\{s_{t}\in R^{3+N}\left|\begin{array}{l} X_{min}\leq x_{t}\leq X_{max},\\ Y_{min}\leq y_{t}\leq Y_{max},\\ Z_{min}\leq z_{t}\leq Z_{max},\\ {\left\|\Psi \left(p_{t}\right)\right\|}_{2}\leq \xi_{max} \end{array}\right.\right\}$$

where parameters such as $X_{min},X_{max}$ define the spatial boundary coordinate extrema of the target water area; $\xi_{max}$ is the upper bound of the two-norm of the global residual vector, which is set according to the maximum communication line-of-sight and is utilized to prevent the state space from diverging. The spatial coordinate bounds in Equation (56) are consistent with the feasible localization region Ω defined in Equation (3).

(2) Model Construction of Continuous Action Space

Since underwater localization is essentially a high-precision coordinate root-finding problem in a continuous space, severe “curse of dimensionality” and truncation errors will be caused by traditional discretized action spaces. Therefore, the action $a_{t}\in A$ of the agent is defined in this paper as a continuous movement step vector along the three orthogonal coordinate axes in the 3D space, i.e., $a_{t}={\left[\Delta x_{t},\Delta y_{t},\Delta z_{t}\right]}^{T}$.

(57) $$a_{t}∼\pi_{\theta }\left(a_{t}\left|s_{t}\right.\right)=\frac{1}{\sqrt{{\left(2\pi \right)}^{3}\det\left(\sum_{\theta }\left(s_{t}\right)\right)}}\exp\left(-\frac{1}{2}{\left(a_{t}-\mu_{\theta }\left(s_{t}\right)\right)}^{T}{\sum_{\theta }\left(s_{t}\right)}^{-1}\left(a_{t}-\mu_{\theta }\left(s_{t}\right)\right)\right)$$

where θ is the set of parameters to be optimized during the Actor policy update process. $\mu_{\theta }\left(s_{t}\right)\in R^{3}$ is the mean vector of the Gaussian distribution, indicating the optimal expected step size for the agent to approach the extremum of the objective function defined in Section 5.2 under the current state $s_{t}$. $\sum_{\theta }\left(s_{t}\right)\in R^{3\times 3}$ is a diagonal covariance matrix, representing the exploration variance of the action output. In the early stages of optimization, kinetic energy is endowed to the agent by a larger variance to escape from local minimum regions.

To ensure the stationarity of the position search transition process and prevent the “overshooting” phenomenon caused by gradient explosion, the continuous action space A cannot be unbounded. A saturation constraint function that dynamically shrinks with the search iteration number t is imposed in this paper, such that the actual action space is constrained to satisfy:

(58) $$A=\left\{a_{t}\in R^{3}\left|{\left\|a_{t}\right\|}_{\infty }=\max\left(\left|\Delta x_{t}\right|,\left|\Delta y_{t}\right|,\left|\Delta z_{t}\right|\right)\leq \kappa \cdot e^{-\lambda_{a}t}\right.\right\}$$

where κ is the initially set maximum spatial search step size (e.g., κ=5.0 m), $e^{-\lambda_{a}t}$ is the exponential action envelope decay factor, and $\lambda_{a}>0$ is the decay rate. The absolute stability of the position search update equation $p_{t+1}=p_{t}+a_{t}$ is not only guaranteed by this Dynamic Bound Constraint, but a mathematical foundation is also laid for the subsequent realization of the adaptive optimization logic characterized by “large-step global exploration in the early stage and fine-grained local exploitation in the later stage”.

#### 5.3.2. Improvement Strategy

(1) Actor-Critic Optimization Mechanism Based on Gaussian Process

In unknown underwater environments without offline data support, the global state-value mapping cannot be obtained by the agent in advance. Therefore, the GPTD algorithm and NPG are introduced in this subsection to construct an Actor-Critic continuous optimization architecture based purely on online exploration trajectories.

To avoid notational ambiguity, $V\left(s\right)$ denotes the scalar state-value function, $\theta \in R^{d_{\theta }}$ denotes the Actor parameter vector, and $\pi_{\theta }\left(\left.a\right|s\right)$ denotes the continuous Gaussian policy. In the following, $F_{\pi }\left(\theta \right)$ is used exclusively for the Fisher information matrix of the policy, whereas $I_{geo}\left(p\right)$ denotes the geometric information matrix used in the GDOP calculation.

1. Value Evaluation (Critic)

The core task of the Critic network is to evaluate the long-term expected value $V\left(s_{t}\right)$ of the current state $s_{t}$. To accurately fit the value surface mapped by the highly non-convex localization objective function in Section 5.2, the state-value function is assumed to follow a GP prior with a mean of zero, i.e., $V\left(s\right)∼GP\left(0,k\left(s,s^{\prime }\right)\right)$.

First, to measure the nonlinear similarity between any two states $s_{i}$ and $s_{j}$ in the state space (which contains the current coordinates and multi-link residuals), a Squared Exponential (SE) kernel function with Automatic Relevance Determination (ARD) is constructed in this paper:

(59) $$k\left(s_{i},s_{j}\right)=\sigma_{f}^{2}\exp\left(-\frac{1}{2}{\left(s_{i}-s_{j}\right)}^{T}\Lambda^{-1}\left(s_{i}-s_{j}\right)\right)$$

Before the kernel evaluation, each dimension of the composite state vector is normalized to [0, 1] using the corresponding lower and upper bounds defined in Equation (56). Consequently, the ARD length scales are dimensionless, and the coordinate and residual components contribute to the kernel evaluation on comparable numerical scales.

Here, $s_{i},s_{j}\in R^{3+N}$ are the composite state vectors defined in Section 5.3.1, encompassing the 3D physical coordinates and the N-dimensional MVUE ranging residuals. $\sigma_{f}^{2}\in R^{+}$ is the signal variance of the Gaussian process, by which the overall amplitude prior of the value function surface is controlled. $\Lambda =\mathrm{diag}\left(l_{1}^{2},l_{2}^{2},\ldots ,l_{3+N}^{2}\right)\in R^{\left(3+N\right)\times \left(3+N\right)}$ is the length-scale diagonal matrix, and the sensitivity weights of different state dimensions (coordinate dimensions and ranging links with different confidence levels) to the value evaluation are determined by their diagonal elements.

During online interaction, an online state dictionary $D_{t}=d_{1},\ldots ,d\left(M_{t}\right)$ is maintained, where $M_{t}$ is the current dictionary size. The kernel feature vector of an arbitrary state s is defined as $\phi_{t}\left(s\right)=\left[[k{\left(s,d\right)}_{1},\ldots ,k\left(s,d\left(M_{t}\right)\right){]}^{T}\in R\left(M\right)\right]$.

The online GPTD Critic maintains the posterior mean vector $m\left(t-1\right)\in R\left(M_{t}\right)$ and posterior covariance matrix $P\left(t-1\right)\in R\left(M_{t}\times M_{t}\right)$. Let $w\in R^{M_{t}}$ denote the latent kernel coefficient vector of the state-value function. Conditioned on the online transition history $H_{t-1}$, its posterior distribution is represented as $w\left|H_{t-1}\right.\sim N\left(m_{t-1},P_{t-1}\right)$. The posterior mean and variance of the state value are expressed as

(60) $${\hat{V}}_{t-1}\left(s\right)=\phi_{t}{\left(s\right)}^{T}m_{t-1},\;{\hat{\sigma }}_{V,t-1}^{2}\left(s\right)=\phi_{t}{\left(s\right)}^{T}P_{t-1}\phi_{t}\left(s\right)$$

For the transition $\left(s_{t^{\prime }},a_{t^{\prime }},r_{t^{\prime }},s_{t+1}\right)$, the GPTD observation model is obtained from the Bellman equation as

(61) $$h_{t}=\phi_{t}\left(s_{t}\right)-\gamma \phi_{t}\left(s_{t+1}\right),\;r_{t}=h_{t}^{T}w+\varepsilon_{t},\;\varepsilon_{t}∼N\left(0,\sigma_{\mathrm{TD}}^{2}\right)$$

where γ∈[0,1) is the discount factor and $\sigma_{TD}^{2}$ denotes the variance of the Bellman-observation noise. The TD innovation and the corresponding Bayesian gain are then calculated as

(62) $$\delta_{t}=r_{t}-h_{t}^{T}m_{t-1}=r_{t}+\gamma {\hat{V}}_{t-1}\left(s_{t+1}\right)-{\hat{V}}_{t-1}\left(s_{t}\right),\;q_{t}=\frac{P_{t-1}h_{t}}{\sigma_{\mathrm{TD}}^{2}+h_{t}^{T}P_{t-1}h_{t}}$$

Accordingly, both the posterior mean and posterior covariance of the online GPTD Critic are recursively updated as

(63) $$m_{t}=m_{t-1}+q_{t}\delta_{t},\;P_{t}=P_{t-1}-q_{t}h_{t}^{T}P_{t-1}$$

Therefore, m_t_ determines the posterior value estimate, whereas P_t_ quantifies the remaining epistemic uncertainty caused by insufficient exploration. All quantities in Equations (60)–(63) are updated using only the currently observed transition and do not require an offline training dataset. After completing the posterior update at iteration t, the newly visited state **s**_t+1_ is appended to the online dictionary for the next iteration, i.e., D_*t*+1_ = D*_t_* U{**s**_t+1_}. The posterior mean and covariance are correspondingly augmented by a zero prior mean and the kernel prior variance $\sigma_{f}^{2}$, so that their dimensions remain consistent with the expanded dictionary.

2. Continuous Policy Update (Actor)

The Actor network is responsible for updating the Gaussian policy parameters θ defined in Section 5.3.1 according to the value feedback provided by the Critic. To overcome the problems of “Overshooting” and “Premature Convergence” easily caused by ordinary policy gradients in the complex, non-convex solution spaces underwater, the NPG based on the Fisher information matrix is introduced on the parameter update manifold in this paper.

The one-step TD innovation in Equation (62) provides a sample estimate of the conventional advantage. To encourage uncertainty-directed exploration, the posterior standard deviation of the next state is introduced as an Upper Confidence Bound term. The uncertainty-compensated scalar advantage is therefore defined as:

(64) $${\hat{A}}_{t}^{\mathrm{GP}}\left(s_{t},a_{t}\right)=\delta_{t}+\eta_{\mu }\sqrt{{\hat{\sigma }}_{V,t-1}^{2}\left(s_{t+1}\right)}$$

where ${\hat{A}}_{t}^{\mathrm{GP}}\in R$ is a scalar advantage estimate and $\eta_{\mu }\geq 0$ is the GP uncertainty-exploration coefficient. A larger $\eta_{\mu }$ assigns greater preference to actions leading to insufficiently explored states. It should be emphasized that Equation (64) defines only the scalar advantage used for the policy update. The three-dimensional coordinate transition remains $p_{t+1}=\Pi_{\Omega }\left(p_{t}+a_{t}\right)$, as defined by the continuous action and boundary constraints in Equations (57) and (58).

For the Gaussian policy in Equation (57), the policy parameter vector θ contains the parameters of the mean function and the diagonal log-standard-deviation function. Its log-likelihood is $\log\pi_{\theta }\left(a\left|s\right.\right)=-\frac{1}{2}\left[{\left(a-\mu_{\theta }\right)}^{T}\sum_{\theta }^{-1}\left(a-\mu_{\theta }\right)+\log\det\left(2\pi \sum_{\theta }\right)\right]$.

Let $B_{t}^{on}$ denote the most recent on-policy transition batch and define the score vector $g_{t}=\nabla_{\theta }\log\pi_{\theta }\left(a_{t}\left|s_{t}\right.\right)\in R^{d_{\theta }}$.

The theoretical policy Fisher information matrix is the expectation of $g_{\tau }g_{\tau }^{T}$. In implementation, it is approximated using the following damped empirical estimator:

(65) $${\hat{F}}_{\pi ,t}=\frac{1}{\left|B_{t}^{on}\right|}\sum_{\tau \in B_{t}}g_{\tau }g_{\tau }^{T}+\epsilon_{F}I_{d_{\theta }}$$

where ${\hat{F}}_{\pi ,t}\in R^{d_{\theta }\times d_{\theta }},\;\epsilon_{F}>0$ is a numerical damping coefficient, and $I_{d_{\theta }}$ is the identity matrix. The damping term guarantees that the empirical Fisher matrix remains positive definite and numerically solvable when the trajectory batch is short or the score vectors are nearly linearly dependent.

Using the uncertainty-compensated advantage in Equation (64), the empirical policy-gradient vector is calculated as ${\hat{g}}_{\pi ,t}=\frac{1}{\left|B_{t}^{on}\right|}\sum_{\tau \in B_{t}^{on}}g_{\tau }{\hat{A}}_{\tau }^{\mathrm{GP}}$.

Instead of explicitly calculating the matrix inverse, the natural-gradient direction $d_{t}$ is obtained by solving the damped linear system

(66) $${\hat{F}}_{\pi ,t}d_{t}={\hat{g}}_{\pi ,t},\;\theta_{t+1}=\theta_{t}+\eta_{a}d_{t}$$

where $\eta_{a}>0$ is the Actor learning rate. In implementation, the linear system is solved using a numerically stable linear solver rather than explicitly forming ${\hat{F}}_{\pi ,t}^{-1}$. The Fisher preconditioner rescales the ordinary policy gradient according to the local sensitivity of the policy distribution, thereby improving the conditioning and stability of the continuous-action update.

(2) Multi-Stage Adaptive Reward Function Based on Geometric Topology Potential Field (GTM-Reward)

In standard RL frameworks, a simple Root Mean Square Error (RMSE) penalty is usually adopted by the reward function. However, the solution space of underwater localization is affected not only by the scalar dimension of ranging errors but also by the geometric characteristics of the spatial layout of anchor nodes. If the optimization relies solely on “algebraic errors”, the agent is highly prone to becoming trapped in local minima within ill-conditioned topological regions where the nodes are coplanar or aggregated. To this end, the local GDOP is introduced in this subsection as the spatial topology potential energy, and a multi-stage adaptive switching mechanism is designed. Its complete mathematical evolution model is derived as follows:

1. Basic Objective Function Penalty Term

First, the fused-distance WLS objective constructed in Section 5.2 is mapped to the basic algebraic penalty $L_{base}\left(s_{t}\right)$ of the agent under the current state $s_{t}$. To improve robustness against residual long-tailed NLOS errors and model mismatch, the quadratic loss in Equation (52) is extended using a generalized Huber loss, while the MVUE-fused acoustic–optical distance and the WLS link weight defined in Equation (53) are retained. The basic penalty is expressed as

(67) $$L_{base}\left(s_{t}\right)=\sum_{i=1}^{N}\omega_{i}\cdot H_{\delta }\left({\hat{d}}_{fuse,i}-\sqrt{{\left(x_{t}-x_{i}^{a}\right)}^{2}+{\left(y_{t}-y_{i}^{a}\right)}^{2}+{\left(z_{t}-z_{i}^{a}\right)}^{2}}\right)$$

The generalized Huber operator $H_{\delta }\left(e\right)$ is defined as

(68) $$H_{\delta }\left(e\right)=\left\{\begin{array}{cc} \frac{1}{2}e^{2} & ,\mathrm{if}\;\left|e\right|\leq \delta \\ \delta \left|e\right|-\frac{1}{2}\delta^{2} & ,\mathrm{if}\;\left|e\right|>\delta \end{array}\right.$$

In Equations (67) and (68), ${\hat{d}}_{fuse,i}$ is the MVUE-fused acoustic–optical observation distance associated with the i−th anchor link, and ${\left(x_{i}^{a},y_{i}^{a},z_{i}^{a}\right)}^{T}$ denotes the known spatial coordinate of the corresponding anchor node. The vector ${\left(x_{t},y_{t},z_{t}\right)}^{T}$ denotes the instantaneous candidate coordinate of the agent at iteration t. The coefficient $\omega_{i}=1/\sigma_{\mathrm{total},i}^{2}>0$ is the WLS anchor-link weight defined in Equation (53), rather than an acoustic or optical modality weight, and δ>0 is the Huber truncation threshold.

Equation (67) is therefore a robust extension of the fused-distance WLS objective in Equation (52), rather than a separate acoustic-only objective. When the absolute residual does not exceed δ, the Huber term is quadratic and $L_{base}\left(s_{t}\right)$ is proportional to the original WLS objective. When a residual becomes excessively large because of remaining NLOS contamination or model mismatch, the penalty changes from quadratic to linear growth, thereby limiting the influence of outlying fused-distance observations and preventing excessively large policy updates.

2. Geometric Topology Perception Mechanism

To endow the agent with the capability of perceiving the spatial structure of surrounding anchor nodes, allowing it to actively move towards regions with the “most robust observation geometric topology”, the Direction Cosine Jacobian Matrix $J\left(p_{t}\right)\in R^{N\times 3}$ corresponding to the current coordinate vector $p_{t}={\left[x_{t},y_{t},z_{t}\right]}^{T}$ is defined as:

(69) $$J\left(p_{t}\right)=\left[\begin{array}{ccc} \frac{\partial {\left\|p_{t}-p_{1}^{a}\right\|}_{2}}{\partial x_{t}} & \frac{\partial {\left\|p_{t}-p_{1}^{a}\right\|}_{2}}{\partial y_{t}} & \frac{\partial {\left\|p_{t}-p_{1}^{a}\right\|}_{2}}{\partial z_{t}}\\ \vdots & \vdots & \vdots \\ \frac{\partial {\left\|p_{t}-p_{N}^{a}\right\|}_{2}}{\partial x_{t}} & \frac{\partial {\left\|p_{t}-p_{N}^{a}\right\|}_{2}}{\partial y_{t}} & \frac{\partial {\left\|p_{t}-p_{N}^{a}\right\|}_{2}}{\partial z_{t}} \end{array}\right]=\left[\begin{array}{ccc} \frac{x_{t}-x_{1}^{a}}{D_{t,1}} & \frac{y_{t}-y_{1}^{a}}{D_{t,1}} & \frac{z_{t}-z_{1}^{a}}{D_{t,1}}\\ \vdots & \vdots & \vdots \\ \frac{x_{t}-x_{N}^{a}}{D_{t,N}} & \frac{y_{t}-y_{N}^{a}}{D_{t,N}} & \frac{z_{t}-z_{N}^{a}}{D_{t,N}} \end{array}\right]$$

where $D_{t,i}={\left\|p_{t}-p_{i}^{a}\right\|}_{2}$ is the instantaneous Euclidean distance. To distinguish the localization geometry from the policy-space Fisher matrix in Equation (65), the weighted geometric information matrix is denoted by $I_{\mathrm{geo}}\left(p_{t}\right)=J{\left(p_{t}\right)}^{T}WJ\left(p_{t}\right)+\lambda_{g}I_{3}$.

The corresponding GDOP-based spatial topology potential is then calculated as:

(70) $$L_{geo}\left(s_{t}\right)=\sqrt{\mathrm{Tr}\left[I_{\mathrm{geo}}{\left(p_{t}\right)}^{-1}\right]}$$

where $\mathrm{Tr}\left[\cdot \right]$ denotes the matrix trace. The quantity $L_{geo}\left(s_{t}\right)$ measures the aggregate geometric amplification of the localization uncertainty along the three coordinate directions. A smaller value indicates a more informative and better-conditioned anchor geometry around the current candidate position. $\lambda_{g}>0$ is a small Tikhonov regularization coefficient, and $I_{3}$ denotes the 3 × 3 identity matrix. The regularization term $\lambda_{g}I_{3}$ ensures that $I_{\mathrm{geo}}\left(p_{t}\right)$ remains positive definite and invertible under nearly coplanar or spatially aggregated anchor configurations, thereby improving the numerical stability of the GDOP calculation.

3. Multi-Stage Adaptive Switching

Under the GP-AC framework, to achieve a smooth transition between global exploration and local exploitation, rigid threshold switching is not adopted in this paper; instead, a high-order dynamic temperature function $\beta \left(s_{t},t\right)$ driven dually by the “basic algebraic error residual” and the “iteration process” is designed:

(71) $$\beta \left(s_{t},t\right)=\beta_{min}+\left(\beta_{max}-\beta_{min}\right)\cdot \frac{1}{1+\exp\left(-\kappa_{\beta }\left(\frac{L_{base}\left(s_{t}\right)}{L_{th}}-1\right)\right)}\cdot \exp\left(-\frac{\lambda_{T}\cdot t}{T_{max}}\right)$$

where $\beta \left(s_{t},t\right)\in \left[\beta_{min},\beta_{max}\right]$ is the outputted dynamic temperature weight coefficient, and its value interval is usually restricted between [0.1, 0.9]. $L_{th}\in R^{+}$ is the preset residual convergence critical constant; $\kappa_{\beta }\in R^{+}$ is the smoothness adjustment scalar of the sigmoid logistic curve; t and $T_{max}$ are the current search step number and the maximum allowable search step number, respectively; and $\lambda_{T}>0$ is the time decay rate.

Synthesizing all the aforementioned mechanisms, the immediate reward $r\left(s_{t}\right)$ fed back to the GP-AC agent by the environment under state $s_{t}$ is manifested as the nonlinear composite superposition of the algebraic error penalty, the geometric topology penalty, and the out-of-bounds penalty:

(72) $$r\left(s_{t}\right)=-\underset{\mathrm{Dynamic}\;\mathrm{Composite}\;\mathrm{Penalty}}{\left[\beta \left(s_{t},t\right)\cdot \frac{L_{base}\left(s_{t}\right)}{\max(L_{base})}+\left(1-\beta \left(s_{t},t\right)\right)\cdot \frac{L_{geo}\left(s_{t}\right)}{\max\left(L_{geo}\right)}\right]}-\underset{\mathrm{Boundary}\;\mathrm{Constraint}}{\xi \cdot \max{\left(0,{\left\|p_{t}\right\|}_{\infty }-B_{bound}\right)}^{2}}$$

Through this composite reward model, the optimization process is automatically decoupled into two smoothly transitioning stages:

The first stage (Global Exploration Period): When $L_{base}\left(s_{t}\right)\gg L_{th}$, the temperature coefficient $\beta \left(s_{t},t\right)$ approaches $\beta_{max}$ in the early stage of optimization. At this time, the entire reward term is dominated by the basic objective function, and the Gaussian Process (GP) is utilized by the agent to quickly cross the macroscopic error surface within a large range, approaching the approximate region where the global extremum is located.

The second stage (Local Topology Exploitation Period): As the residual shrinks below the critical value and time elapses, $\beta \left(s_{t},t\right)$ decays to $\beta_{min}$ with a dual exponential and logistic rate. At this time, the weight of the GDOP potential energy term is fully highlighted. The agent is forced by this mechanism to abandon large-scale trial and error; in deep-water areas densely populated with local minima, fine-grained spatial refinement is performed along the topology-improving direction characterized by the weighted geometric information matrix $I_{\mathrm{geo}}\left(p_{t}\right)$. Thereby, deviations caused by poor node layouts are substantially mitigated, and numerical oscillations at the end of convergence are eliminated. The pseudocode of the proposed GP-AC algorithm is presented in Algorithm 1.
**Algorithm 1:** A Geometric Topology Perception-Enhanced Multi-Stage Reinforcement Learning Algorithm**Input:** The 3D coordinates of known anchor nodes ${\left\{p_{i}^{anc}\right\}}_{i=1}^{N}$, MVUE-fused acoustic–optical observation distances ${\left\{{\hat{d}}_{fuse,i}\right\}}_{i=1}^{N}$, total fused ranging error variances ${\left\{\sigma_{\mathrm{total},i}^{2}\right\}}_{i=1}^{N}$, and the corresponding WLS link weights ${\left\{\omega_{i}=1/\sigma_{\mathrm{total},i}^{2}\right\}}_{i=1}^{N}$, maximum number of iterations *T*_max_, Actor learning rate η_a_, discount factor γ, initial maximum action step *B*_0_, action-envelope decay rate λ_a_, uncertainty-exploration coefficient η_u_, temporal switching decay rate λ_T_, Bellman-observation noise variance $\sigma_{TD}^{2}$, Fisher damping coefficient $\epsilon_{F}$, geometric regularization coefficient λ_g_, and on-policy batch size *N*_B_.
**Output:** Optimal 3D estimated coordinates of the unknown node ${\hat{x}}^{*}$
1: System Initialization2: For each independent localization run, randomly initialize the Actor parameter vector θ_0_; the Actor parameters learned from previous runs are not retained or reused.3: Randomly initialize the starting coordinate p_0_ within the constrained underwater region $\Omega =\left[x_{\min},x_{\max}\right]\times \left[y_{\min},y_{\max}\right]\times \left[z_{\min},z_{\max}\right]$ and construct the corresponding initial state s_0_.4: Initialize the online GPTD dictionary as D_0_ = {s_0_}, the posterior mean as m_0_ = 0, the posterior covariance as $P_{0}=\sigma_{f}^{2}I$, and the on-policy transition batch as $B_{0}^{\mathrm{on}}=\emptyset$.5: for $t=0,1,2,\ldots ,T_{\max}-1$ do6: State Construction and Action Execution7: Construct and normalize the current composite state by combining the current coordinate and the WLS-weighted fused-distance residual vector defined in Equation (55).8: A continuous 3D movement step size a_t_ is sampled by the Actor according to the Gaussian policy distribution $\pi_{\theta_{t}}\left(a_{t}\left|{\tilde{s}}_{t}\right.\right)∼N\left(\mu \left({\tilde{s}}_{t}\left|\theta_{t}\right.\right),\Sigma \right)$.9: The action is clipped by applying a dynamic boundary constraint, a_t_ ∈ [−B_t_, B_t_], where the envelope shrinkage function is defined as $B_{t}=B_{0}\cdot e-\lambda_{a}\cdot t$.10: Execute the action and update the candidate coordinate as **p**_(t+1)_ = **Π**_Ω_ (**p**_t_ + **a**_t_), where Ω is the feasible localization region defined in Equation (3).11: GTM-Reward Adaptive Calculation12: Calculate the basic algebraic penalty L_base_(s_t+1_) using the MVUE-fused acoustic–optical distances, the WLS link weights, and the generalized Huber operator according to Equation (67).13: Construct the direction-cosine Jacobian matrix $J\left(p+1\right)$, calculate the weighted geometric information matrix $I_{\mathrm{geo}}\left(p_{t+1}\right)=J{\left(p_{t+1}\right)}^{T}WJ\left(p_{t+1}\right)+\lambda_{g}I_{3}$, and obtain the topology potential $L_{geo}\left(s_{t+1}\right)$ according to Equation (70).14: Calculate the dynamic switching coefficient $\beta \left(s_{t+1},t\right)$ according to the current algebraic residual and iteration progress using Equation (71).15: Calculate the composite immediate reward r_t_ by combining the normalized algebraic penalty, the topology potential, and the boundary penalty according to Equation (72). 16: Store the current transition (**s**_t_, **a**_t_, **r**_t_, **s**_t+1_) in the on-policy batch $B_{t}^{on}$. If $\left|B_{t}^{on}\right|>N_{B}$, retain only the most recent $N_{B}$ transitions.17: Critic Value Evaluation and Online GPTD Update18: Construct the kernel feature vectors $\phi_{t}\left(s_{t}\right)$ and $\phi_{t}\left(s_{t+1}\right)$, and calculate the Bellman feature vector $h(t)=\phi_{t}\left(s_{t}\right)-\gamma \phi_{t}\left(s_{t+1}\right)$.19: Calculate the TD innovation $\delta_{t}$ and Bayesian gain $q_{t}$ according to Equation (62).20: Update the GPTD posterior mean $m_{t}$, posterior covariance $p_{t}$, and next-state value variance according to Equations (60)–(63); then append $s_{t+1}$ to the online dictionary and augment the posterior parameters for the next iteration. 21: Calculate the scalar uncertainty-compensated advantage ${\hat{A}}_{t}^{\mathrm{GP}}$ according to Equation (64)22: Actor Policy Update: Manifold Optimization Based on Natural Gradient23: Calculate the score vectors and construct the damped empirical policy Fisher matrix ${\hat{F}}_{\pi ,t}$ and the policy-gradient vector ${\hat{g}}_{\pi ,t}$ using the recent on-policy transition batch.24: Solve ${\hat{F}}_{\pi ,t}d_{t}={\hat{g}}_{\pi ,t}$ and update the Actor parameters as $\theta_{t+1}=\theta_{t}+\eta_{a}d_{t}$.25: end for26: return the final state coordinates $x_{T_{max}}$ at the end of the iterations as the optimal 3D coordinate estimate of the unknown node

## 6. Experimental Section

### 6.1. Simulation Parameters and Environment

To ensure a systematic and reproducible evaluation of the proposed method, this section first introduces the simulation platform and hardware/software environment, then specifies the main underwater network, acoustic–optical channel, environmental, and algorithm parameters, and finally defines the evaluation metrics and statistical protocol used in the subsequent experiments.

#### 6.1.1. Simulation Environment

The simulation experiments of this study are implemented based on the Matrix Laboratory (MATLAB) R2019b platform. To comprehensively verify the effectiveness of the algorithm proposed in this paper, a high-fidelity 3D underwater network simulation environment is constructed, which is utilized to simulate the processes of random node deployment, dynamic communication ranging, and RL localization calculation within the underwater acoustic–optical hybrid network. The acoustic–optical heterogeneous propagation loss model and the environment-driven MVUE robust model described in Section 4 are deeply integrated into this simulation environment, and the continuous space optimization algorithm based on GP-AC proposed in Section 5 is embedded.

Not only can the heterogeneous interference of complex hydrological conditions (such as dynamic turbidity, as well as multipath and NLOS effects induced by wind and waves) on the link ranging quality be simulated by this platform, but the sequential decision-making and trajectory exploration processes of the localization agent in the non-convex 3D solution space, which are driven by GDOP and multi-stage adaptive rewards, can also be intuitively reproduced. The specific hardware configurations and software operating environment parameters adopted in the experiments are presented in Table 1.

#### 6.1.2. Simulation Parameter Settings

The experimental scenario is set within a 3D underwater monitoring area of 100 m×100 m×100 m. The underwater environmental parameters are set with reference to the Jerlov water-type classification [46]. The acoustic and optical communication parameters are configured according to the corresponding uncertainty models presented in Section 4.1, where the acoustic ranging uncertainty is primarily affected by the sea-surface wind speed $v_{wind}$ and the shipping-activity factor *s*, while the optical ranging uncertainty is mainly affected by the chlorophyll concentration $C_{chl}$. The key simulation parameter settings are presented in Table 2.

To improve the transparency and reproducibility of the simulation environment, Table 3 summarizes the sensing modules implemented in the simulator, together with their noise models, sampling rates, motion assumptions, and parameter sources. The simulated acoustic and optical observations are generated from physics-based propagation and measurement models rather than from arbitrary empirical perturbations. Because the present study does not employ a dedicated physical testbed, the sampling rates listed in Table 3 represent simulator-side acquisition settings and should not be interpreted as measured specifications of a particular commercial sensor.

The acoustic sampling rate of 100 kHz is higher than twice the maximum frequency of the adopted LFM waveform (27.5 kHz), thereby satisfying the Nyquist sampling condition. The optical sampling rate refers to the electrical output of the photodetector rather than to the optical carrier itself. The environmental variables are updated at a lower rate because their variation is substantially slower than that of the acoustic and optical received signals.

It should also be emphasized that the reinforcement-learning iterations are numerical coordinate-refinement steps in the optimization space and are not sensor-sampling instants or physical motion steps. The current simulator therefore evaluates single-epoch static or quasi-static three-dimensional localization under time-varying sensing uncertainty, rather than continuous trajectory tracking.

#### 6.1.3. Evaluation Metrics

To comprehensively evaluate the localization performance and computational efficiency of the proposed algorithm, the following three key metrics are selected for quantitative analysis in this paper: RMSE, Average Localization Time, and Cumulative Distribution Function (CDF) of the localization error. To ensure the statistical reliability of the reported results and reduce the influence of random node deployment, channel-noise realizations, and algorithm initialization, unless otherwise stated, each simulation condition was independently repeated 100 times using different random seeds. Scalar performance values and plotted curve points are reported as the arithmetic means of the independent runs, whereas distributional results, including CDFs, box plots, standard deviations, and percentile errors, are calculated using the complete set of independent samples. For the statistical localization evaluation in Section 6.3.1 and the non-Gaussian noise robustness evaluation in Section 6.3.3, 200 independent Monte Carlo runs were conducted for each condition.

RMSE is used as the core metric for localization accuracy and reflects the deviation between the estimated and true coordinates. The calculation formula is as follows:

(73) $$RMSE=\sqrt{\frac{1}{M}\sum_{k=1}^{M}\left[{\left(x_{k}-{\hat{x}}_{k}\right)}^{2}+{\left(y_{k}-{\hat{y}}_{k}\right)}^{2}+{\left(z_{k}-{\hat{z}}_{k}\right)}^{2}\right]}$$

where M is the number of Monte Carlo experiments or the total number of unknown nodes, $\left(x_{k},y_{k},z_{k}\right)$ are the true coordinates of the k−th node, and $\left({\hat{x}}_{k},{\hat{y}}_{k},{\hat{z}}_{k}\right)$ are the estimated coordinates outputted by the algorithm. The smaller the RMSE value, the higher the localization accuracy. The average localization time metric is utilized to evaluate the computational complexity and real-time performance of the algorithm. The average CPU execution time (unit: seconds) required for the algorithm to output the final coordinates from initialization to the end of iterations is recorded.

(74) $$T_{avg}=\frac{1}{M}\sum_{k=1}^{M}t_{k}$$

where $t_{k}$ is the execution time of the k−th localization process.

The CDF of the localization error can reflect the distribution of localization errors more meticulously, representing the probability that the localization error is less than a specific threshold δ.

(75) $$CDF\left(\delta \right)=P\left(Error\leq \delta \right)$$

Compared to a single average RMSE value, the robustness of the algorithm can be demonstrated by the CDF. For instance, two curves may share the same RMSE, but for an algorithm whose CDF curve rises faster and possesses a shorter long tail, this indicates that small localization errors are obtained in most cases and that localization failures or extreme deviations are rare, providing stronger evidence of algorithm stability.

### 6.2. Verification and Analysis of the Heterogeneous Ranging Error Model

To prove the superiority of the environment-aware adaptive linear weighted fusion model proposed in Section 4.2 compared to traditional fixed-weight methods, comparative experiments are designed in this section. Environments with dynamically changing water turbidity (chlorophyll concentration $C_{chl}$) and sea states (wind speed $v_{w}$) are simulated in the experiments, and the ranging errors under the two fusion strategies are comparatively analyzed.

#### 6.2.1. Analysis of Fixed-Weight Fused Ranging

In existing literature on underwater multi-modal fusion (such as Reference [5]), due to the lack of real-time perception capability for environmental noise, empirically fixed weight coefficients are usually adopted for data fusion. The fixed-weight fused distance ${\tilde{z}}_{1}$ is defined as:

(76) $${\tilde{z}}_{1}=\lambda_{1}^{\prime }z_{ac}+\lambda_{2}^{\prime }z_{op}$$

**Figure 4:**
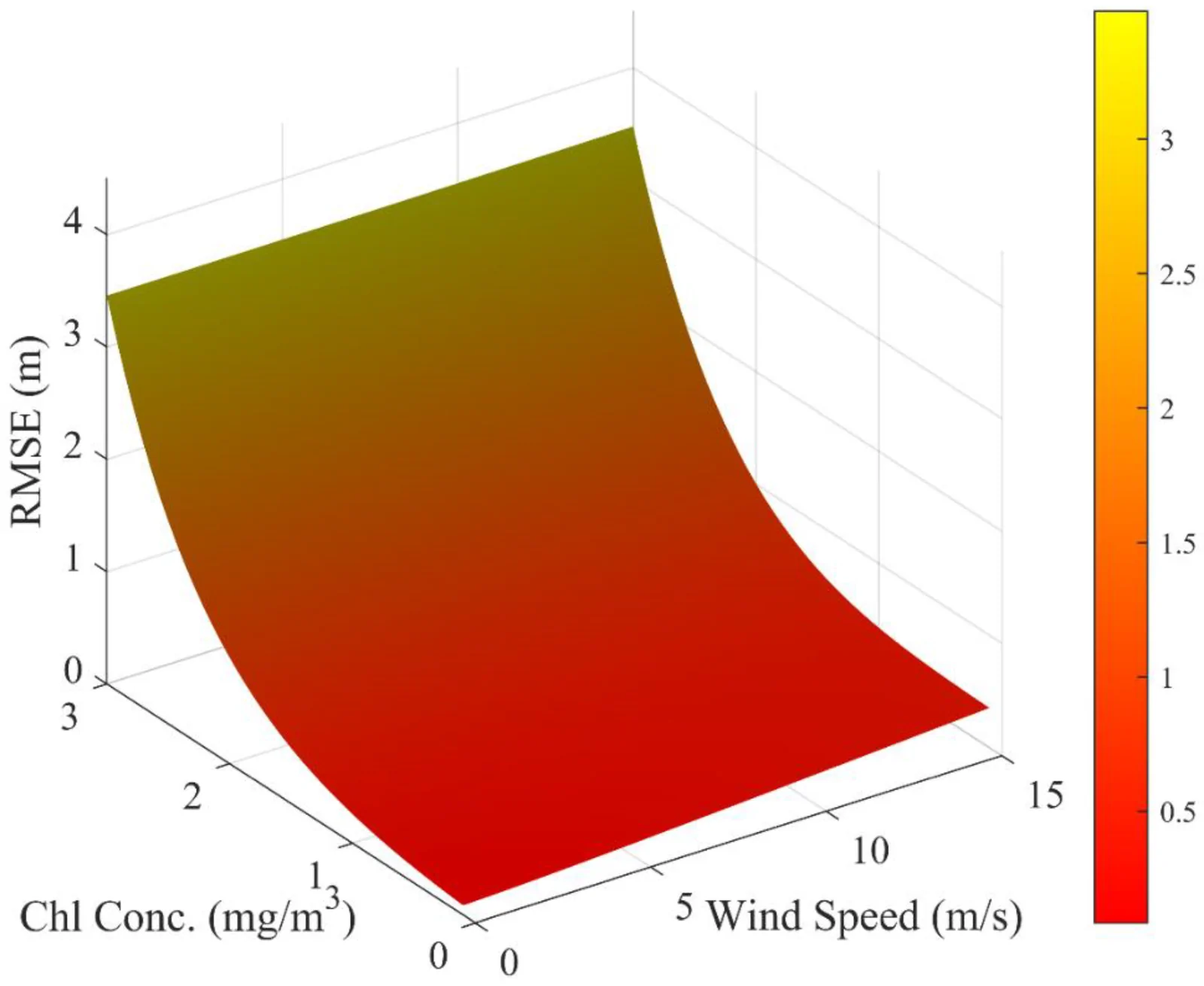
RMSE surface plot under fixed weights.

Referring to relevant studies, the empirical weights are set as $\lambda_{1}^{\prime }=0.3$ (acoustic) and $\lambda_{2}^{\prime }=0.7$ (optical). An a priori assumption is implicitly contained in this configuration: optical ranging is superior to acoustic ranging in most cases. The 3D error surface of RMSE varying with wind speed ($v_{w}$) and chlorophyll concentration ($C_{chl}$) under the fixed-weight strategy is illustrated in Figure 4. It can be intuitively observed from the figure that a significant “single-sided steep slope” feature is exhibited by the error surface. In the low-concentration region where $C_{chl}<1.0\;\mathrm{mg}/m^{3}$, the surface is relatively flat; however, as $C_{chl}$ increases to above 2.0 (highly turbid waters), the error surface bulges sharply along the Y-axis direction, forming a towering red error peak (RMSE > 4 m). This indicates that the fixed-weight model is extremely sensitive to water turbidity and cannot adapt to harsh optical environments.

**Figure 5:**
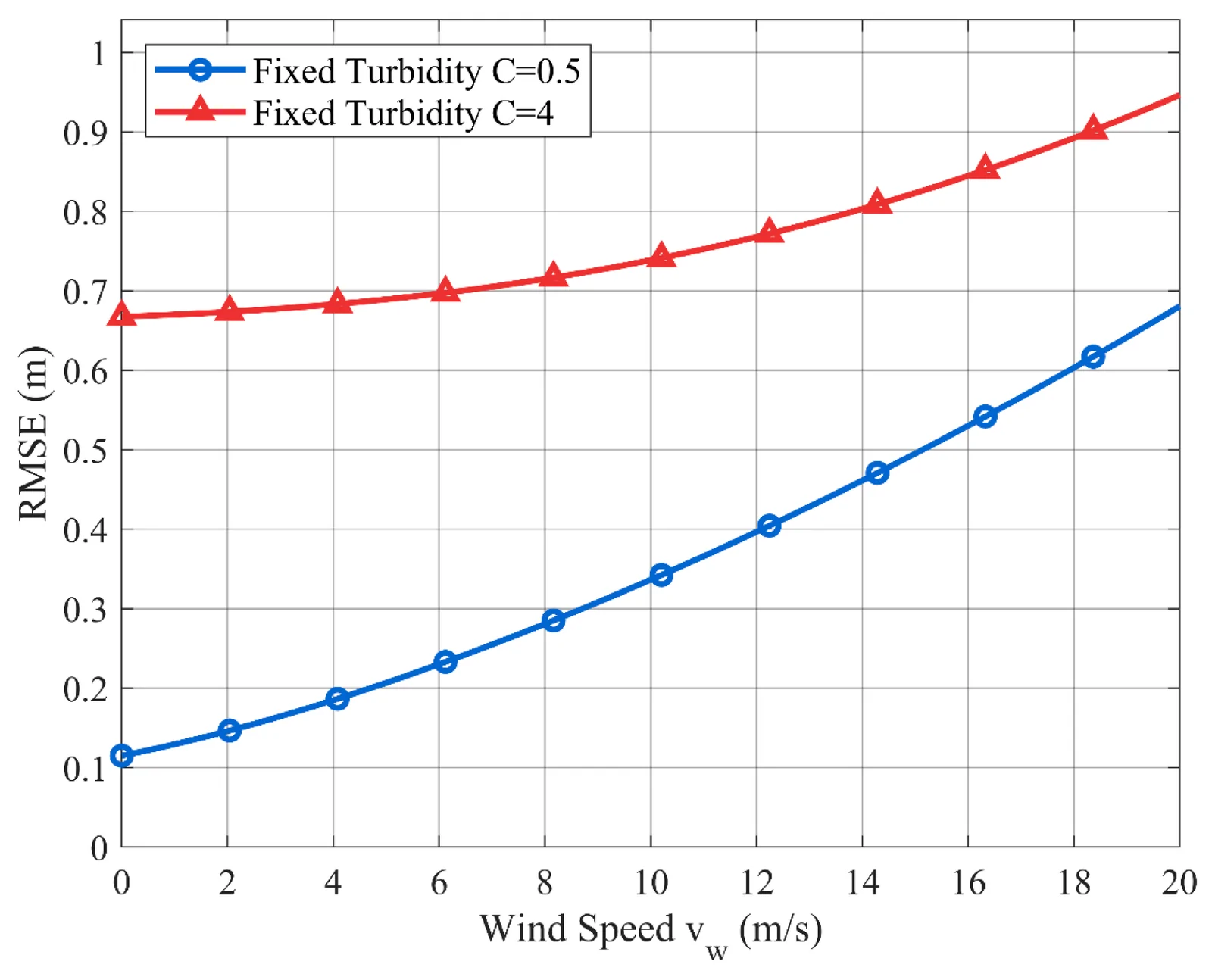
Analysis of the impact of water turbidity on ranging error under the fixed-weight strategy. where *C* denotes the water turbidity *C_chl_*.

To quantify the influence of sea-surface wind speed under different water-quality conditions, Figure 5 presents the ranging RMSE of the fixed-weight strategy under low- and high-turbidity conditions. Figure 5 shows the variation in localization RMSE with sea-surface wind speed *v_w_* under the fixed-weight strategy for clear water ($C_{chl}=0.5\;{\mathrm{mg}/m}^{3}$) and turbid water ($C_{chl}=4\;{\mathrm{mg}/m}^{3}$). Under both water-quality conditions, the RMSE increases monotonically as the wind speed rises from 0 to 20 m/s, indicating the accumulation of environmental noise. Increasing wind speed intensifies wave breaking and bubble generation, thereby enhancing multipath propagation and background noise in the underwater acoustic channel and increasing acoustic ranging uncertainty.

Since the fixed-weight strategy lacks an environment-aware adaptive weighting mechanism, the acoustic weight cannot be reduced when observation quality deteriorates. Consequently, wind-induced acoustic noise is directly propagated into the localization result. Under high turbidity, the optical channel already exhibits a relatively large baseline error, further increasing the overall localization error. Specifically, in clear water, the RMSE increases from 0.11 m to 0.68 m as the wind speed rises from 0 to 20 m/s, whereas under high turbidity, it approaches 1.0 m. These results demonstrate that fixed-weight fusion is sensitive to environmental variations and cannot ensure reliable localization under complex sea conditions.

#### 6.2.2. Analysis of Adaptive Weight Fused Ranging

Aiming at the limitations of fixed weights, the MVUE weights derived in Section 4.2 are adopted in this paper. The weight coefficients of the adaptive fused distance ${\tilde{z}}_{2}$ are calculated in real time according to the current environmental variances:

(77) $$\lambda_{1}=\frac{\sigma_{op}^{2}}{\sigma_{ac}^{2}+\sigma_{op}^{2}},\;\lambda_{2}=\frac{\sigma_{ac}^{2}}{\sigma_{ac}^{2}+\sigma_{op}^{2}}$$

**Figure 6:**
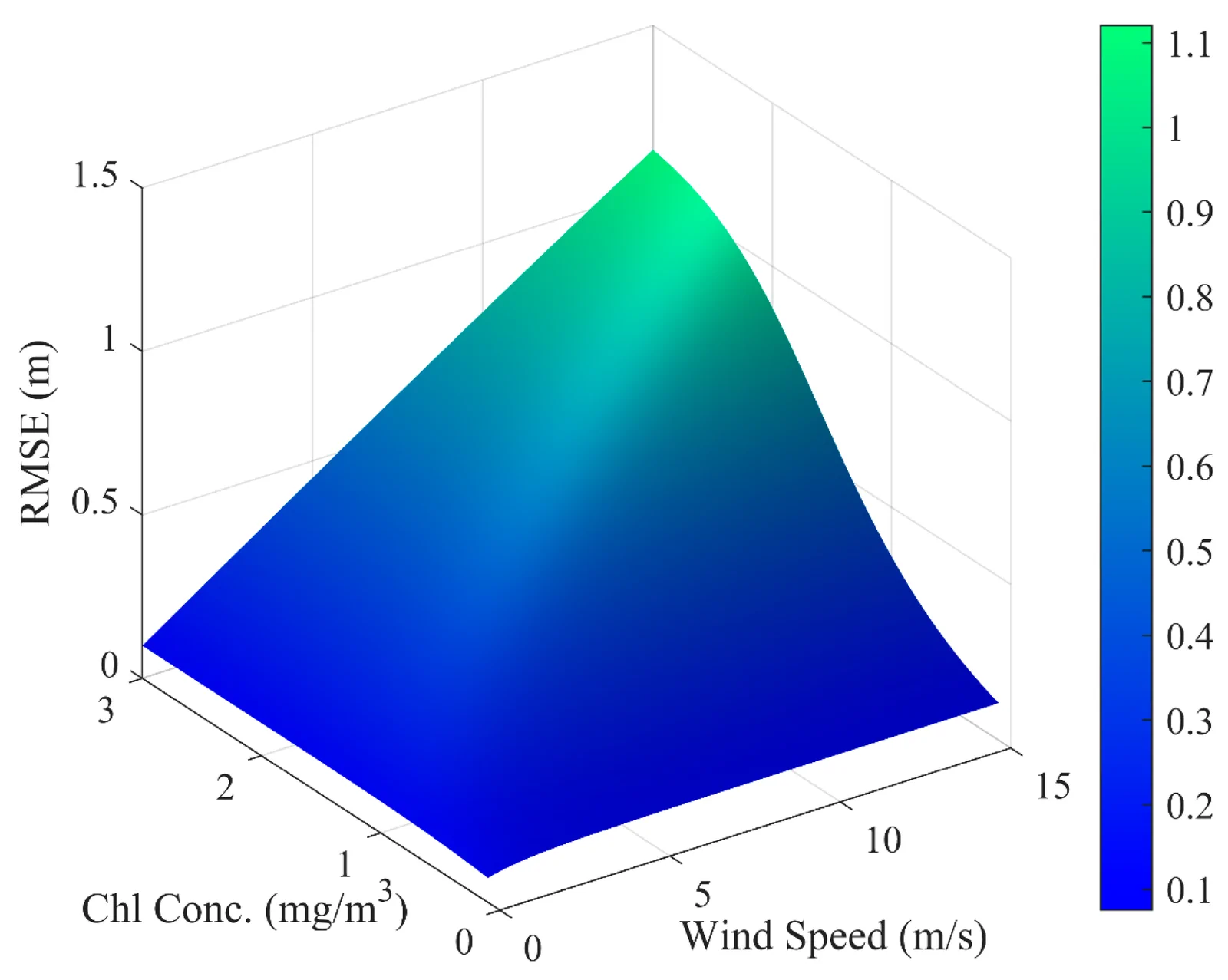
RMSE under adaptive weight.

The 3D error surface under the adaptive weight strategy is illustrated in Figure 6. In sharp contrast to Figure 4, the adaptive algorithm exhibits a much flatter error surface.

Even in the high-turbidity region where the error increases sharply in Figure 4 ($C_{chl}>2.0\;{\mathrm{mg}/m}^{3}$), the RMSE in Figure 6 remains suppressed at a low level of 0.5–0.8 m (dark-blue region). This result indicates that the algorithm successfully detects the degradation of the optical channel and automatically reduces the optical weight λ_2_ to nearly zero. Consequently, the fusion process relies more heavily on the less affected acoustic ranging observations, thereby maintaining robust ranging performance across the entire environmental range. Figure 6 therefore presents the overall response of the adaptive strategy to the joint variations in sea-surface wind speed and chlorophyll concentration.

**Figure 7:**
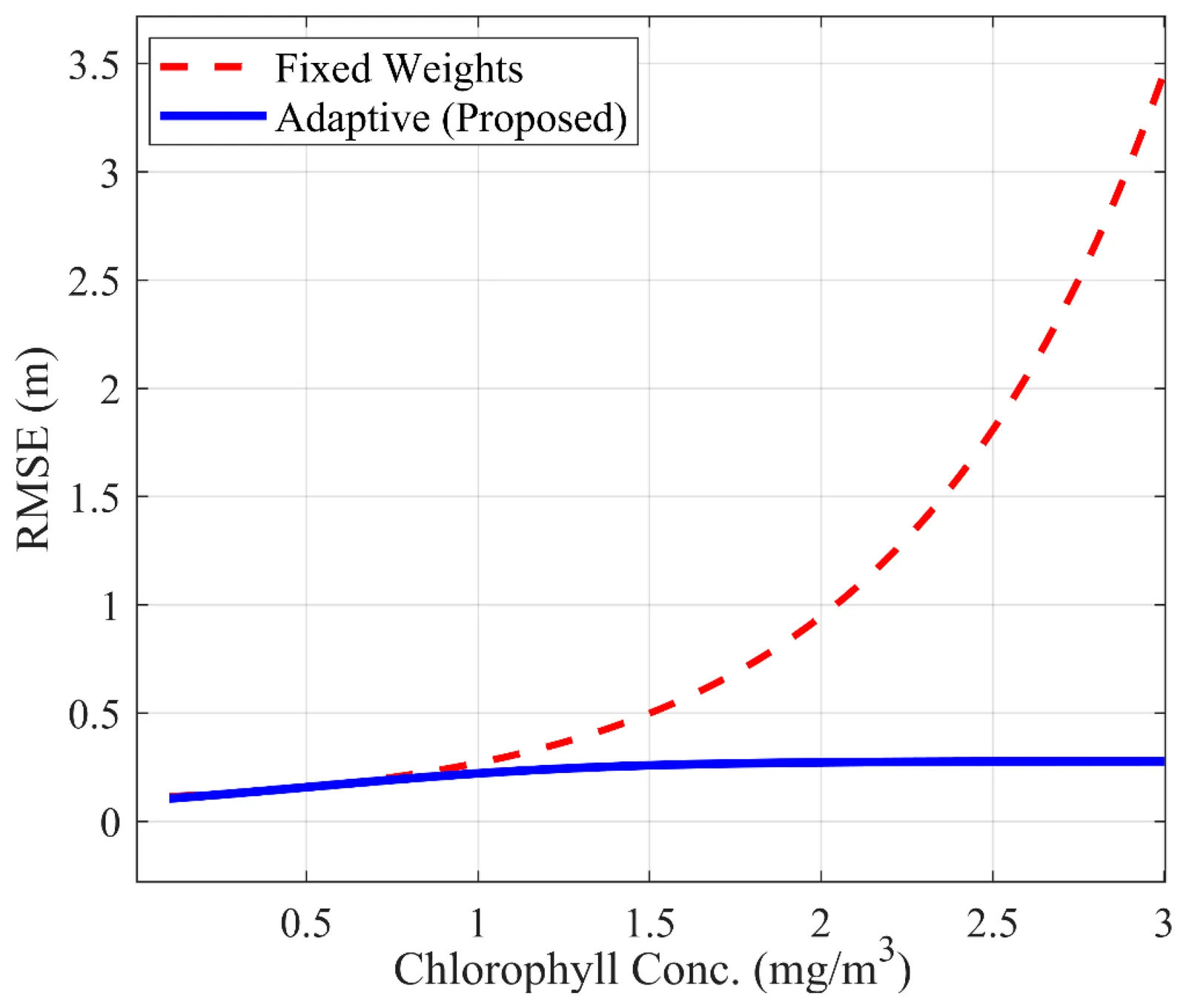
Comparative verification of ranging accuracy varying with water turbidity under different fusion strategies.

To further isolate the influence of water turbidity, Figure 7 compares the ranging RMSE of the fixed-weight and adaptive fusion strategies as the chlorophyll concentration $C_{chl}$ increases. The fixed-weight strategy exhibits pronounced nonlinear error growth. At low turbidity ($C_{chl}\;<3.0{\;\mathrm{mg}/m}^{3}$), the optical channel maintains relatively good transmission performance; however, as turbidity increases, enhanced photon scattering rapidly degrades the optical observations. Since the fixed weights cannot adapt to channel variations, the fusion result becomes increasingly affected by high-variance optical noise.

In contrast, the proposed adaptive fusion strategy remains relatively stable by combining environment-dependent variance characterization with MVUE-based adaptive weighting. As the optical error increases, its confidence weight is dynamically reduced, while greater reliance is placed on the less degraded acoustic observation. At $C_{chl}\;=3.0{\;\mathrm{mg}/m}^{3}$, the RMSE of the fixed-weight strategy reaches approximately 3.45 m, whereas that of the proposed adaptive strategy remains around 0.28 m, corresponding to an approximately 91.9% improvement in ranging accuracy. These results demonstrate that the proposed mechanism effectively suppresses turbidity-induced interference and improves robustness under degraded underwater optical conditions.

**Figure 8:**
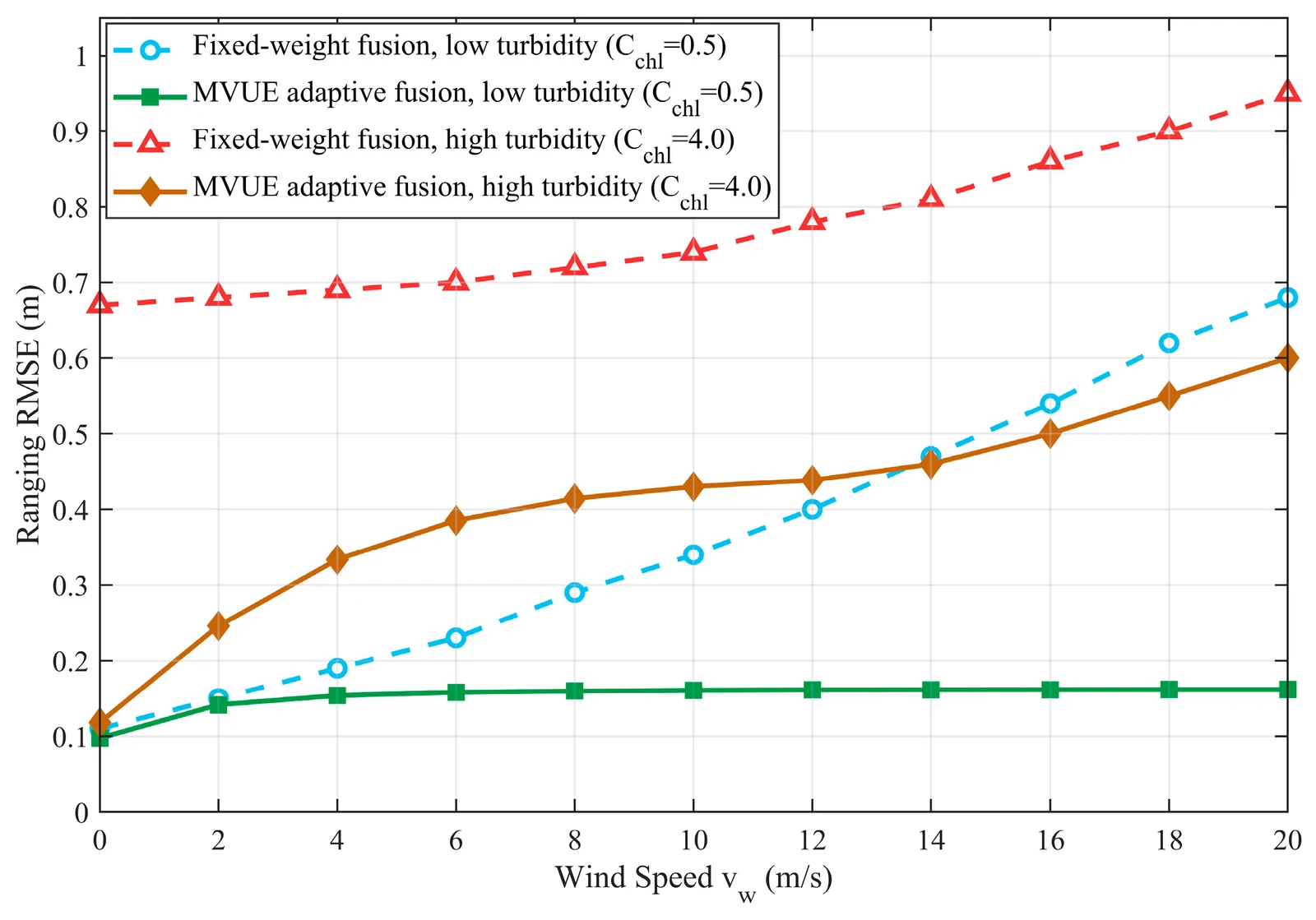
Robustness comparison of ranging error varying with sea surface wind speed under different fusion strategies.

To provide a matched comparison, Figure 8 compares the ranging RMSE of fixed-weight fusion and MVUE adaptive fusion under low- and high-turbidity conditions. Sea-surface wind speed varies from 0 m/s to 20 m/s at 2 m/s intervals, while the chlorophyll concentration is set to $C_{chl}=0.5\;mg/m^{3}$ and $C_{chl}=4\;mg/m^{3}$ for low and high turbidity, respectively; all other environmental and system parameters remain unchanged. The fixed-weight method uses constant acoustic and optical weights, whereas MVUE dynamically adjusts them according to environment-dependent ranging variances.

Under low turbidity ($C_{chl}=0.5\;mg/m^{3}$), the fixed-weight RMSE increases from approximately 0.11 m to 0.68 m as wind speed increases, whereas the MVUE adaptive fusion RMSE rises only from about 0.10 m to 0.16 m. This indicates that when the optical link remains reliable, MVUE suppresses wind-induced acoustic degradation by assigning greater confidence to optical observations. Under high turbidity ($C_{chl}=4\;\mathrm{mg}/m^{3}$), the fixed-weight RMSE increases from approximately 0.67 m to 0.95 m at 20 m/s, while the MVUE RMSE remains approximately 0.60 m.

The crossover between the fixed-weight low-turbidity curve and the MVUE high-turbidity curve in the strong-wind region does not indicate that high turbidity is more favorable. Rather, it reflects the increasing limitation of fixed-weight fusion under severe acoustic disturbance, while MVUE reallocates confidence between acoustic and optical observations according to their ranging variances. At $v_{w}=20\;m/s$, the fixed-weight RMSE values are approximately 0.68 m and 0.95 m under low- and high-turbidity conditions, compared with approximately 0.16 m and 0.60 m for MVUE adaptive fusion. Accordingly, MVUE reduces the ranging RMSE by approximately 76.5% and 36.8%, respectively, demonstrating improved robustness to both wind-induced acoustic degradation and turbidity-induced optical deterioration.

#### 6.2.3. Quantitative Analysis of Multi-Source Factors and Verification of Adaptive Fusion Model

To establish the environment perception model, the independent and coupled effects of communication distance d, sea-surface wind speed $v_{w}$, and chlorophyll concentration $C_{chl}$ on the ranging standard deviation σ are quantitatively analyzed using the control-variable method and the acoustic–optical propagation models developed in Section 4.

(1) Analysis of Linear Cumulative Characteristics of Acoustic Ranging Variance

**Figure 9:**
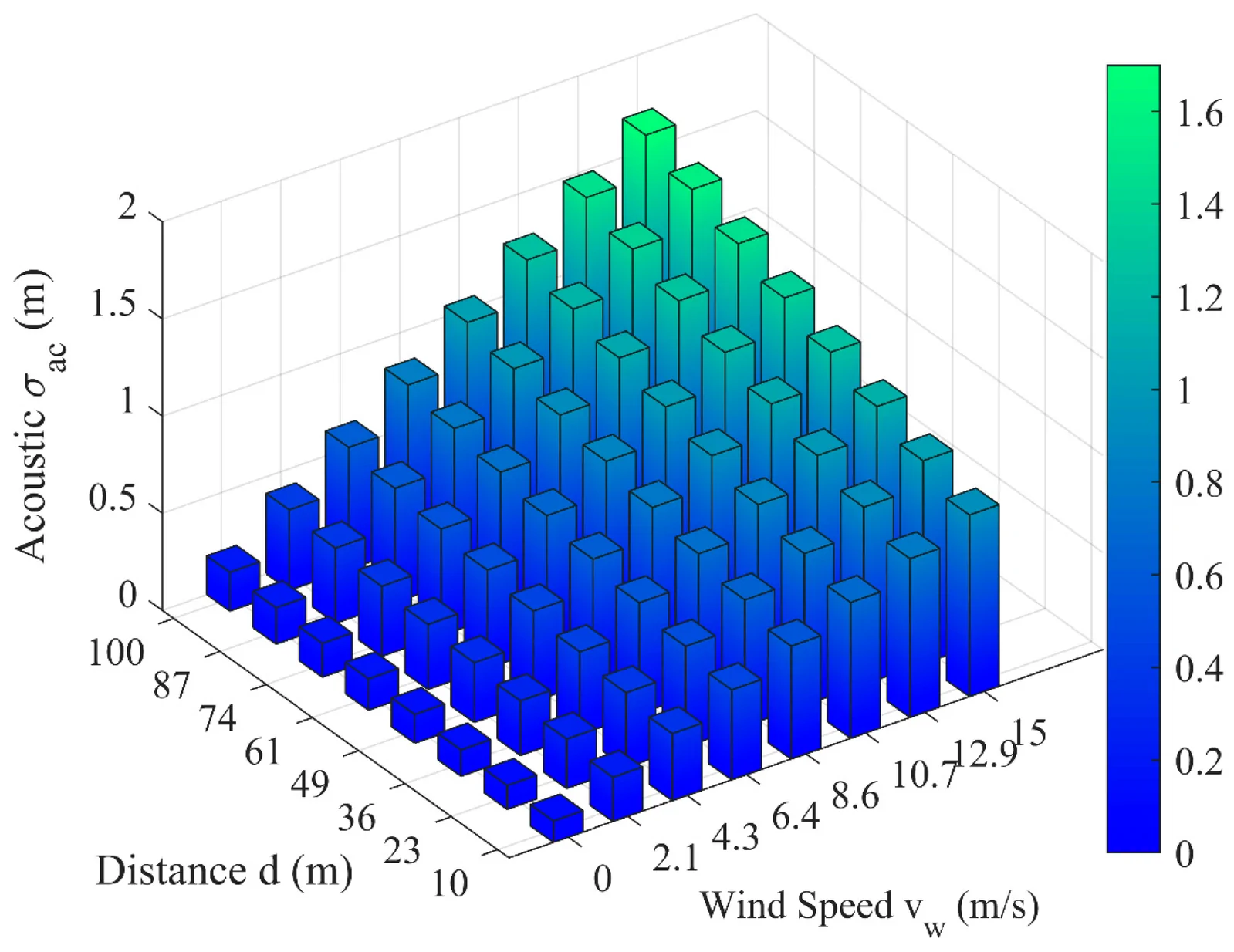
Analysis diagram of influencing factors on acoustic ranging variance.

Figure 9 illustrates the acoustic ranging standard deviation $\sigma_{ac}$ under the combined effects of communication distance $d\in \left[10,100\right]\;m$ and sea-surface wind speed $v_{w}\in \left[0,15\right]\;m/s$. Increasing wind speed raises wind-driven ambient noise and reduces the received SNR, while increasing distance causes greater transmission loss, jointly enlarging the acoustic ranging uncertainty. At d = 50 m, as the wind speed increases from 2 m/s to 14 m/s, the ranging standard deviation rises from 0.30 m to 0.90 m, corresponding to an increase of approximately 200%. The effect of wind speed becomes more pronounced at longer distances because propagation loss amplifies environmental noise effects. Nevertheless, the acoustic error increases gradually and remains bounded within the investigated range, reflecting the soft-degradation characteristic of the acoustic channel. Under severe optical degradation, the MVUE mechanism therefore assigns greater confidence to the acoustic observation, allowing the fused ranging result to degrade gradually rather than diverge abruptly.

(2) Analysis of Nonlinear Avalanche Effect of Optical Ranging Variance

**Figure 10:**
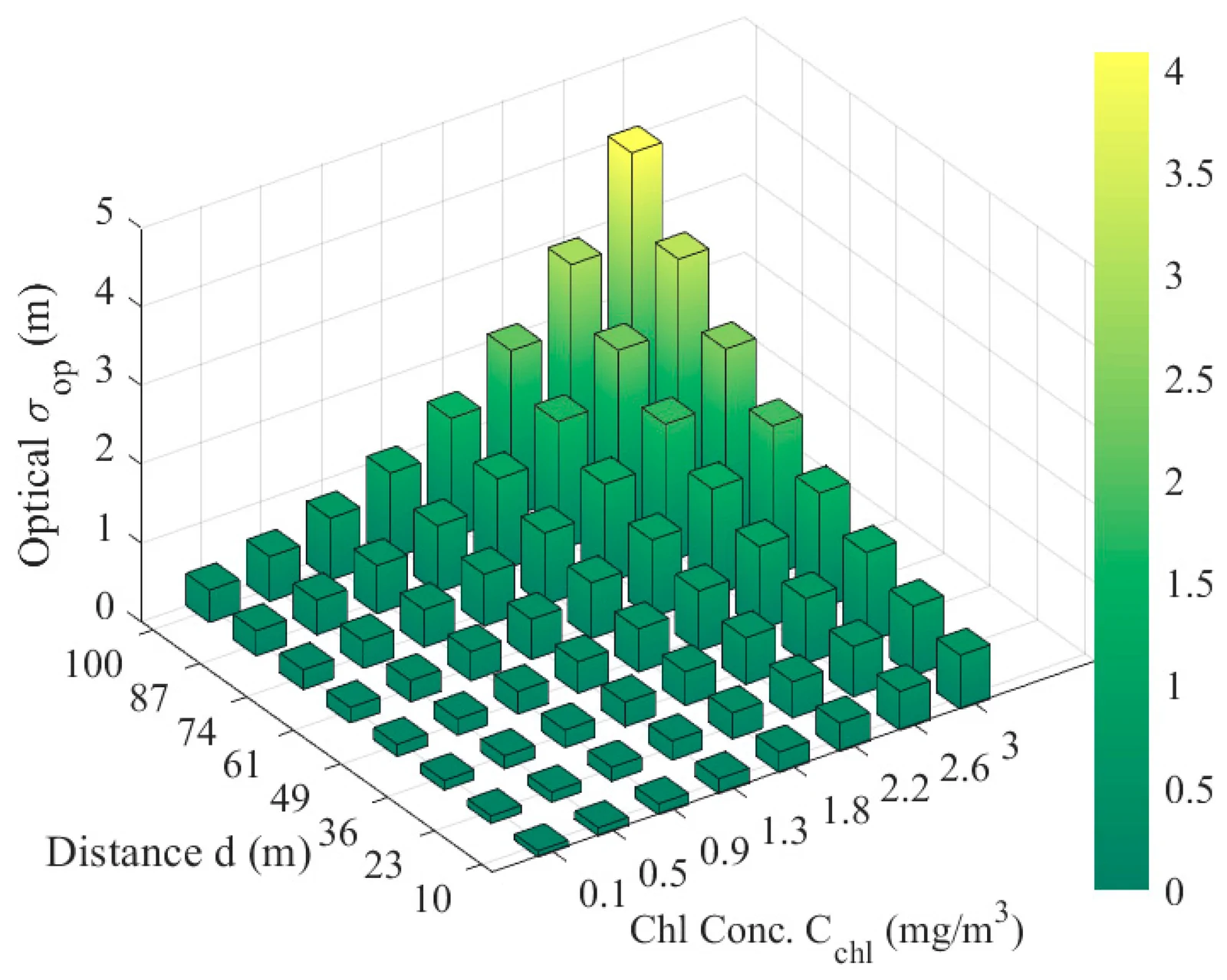
Analysis diagram of influencing factors on optical ranging variance.

The optical ranging standard deviation $\sigma_{op}$ under the combined effects of communication distance and water turbidity (chlorophyll concentration $C_{chl}$) is shown in Figure 10. As water turbidity increases, enhanced absorption and scattering reduce the received optical power, while the nonlinear RSS-to-distance inversion further amplifies the resulting ranging uncertainty. According to the Beer–Lambert law, this degradation increases exponentially with both distance and concentration, producing a clear performance boundary.

Under low turbidity and short to moderate communication distances ($C_{chl}<1.0\;mg/m^{3}$), $\sigma_{op}$ remains below 0.05 m, achieving approximately one order of magnitude higher accuracy than acoustic ranging. Once $C_{chl}$ exceeds 2.0 mg/m^3^, however, the optical error increases sharply. When $C_{chl}$ rises from 1.5 mg/m^3^ to 2.5 mg/m^3^, the standard deviation increases from 0.5 m to more than 4.5 m, corresponding to a degradation exceeding 800%. This clear degradation boundary requires the weighting mechanism to respond rapidly to increasing optical uncertainty. Accordingly, as turbidity increases and the estimated optical ranging variance rises sharply, the optical confidence weight is adaptively reduced toward zero, thereby limiting the influence of severely degraded optical observations on the fused ranging estimate.

(3) Complementarity Analysis of Acoustic–Optical Joint Ranging Variance

**Figure 11:**
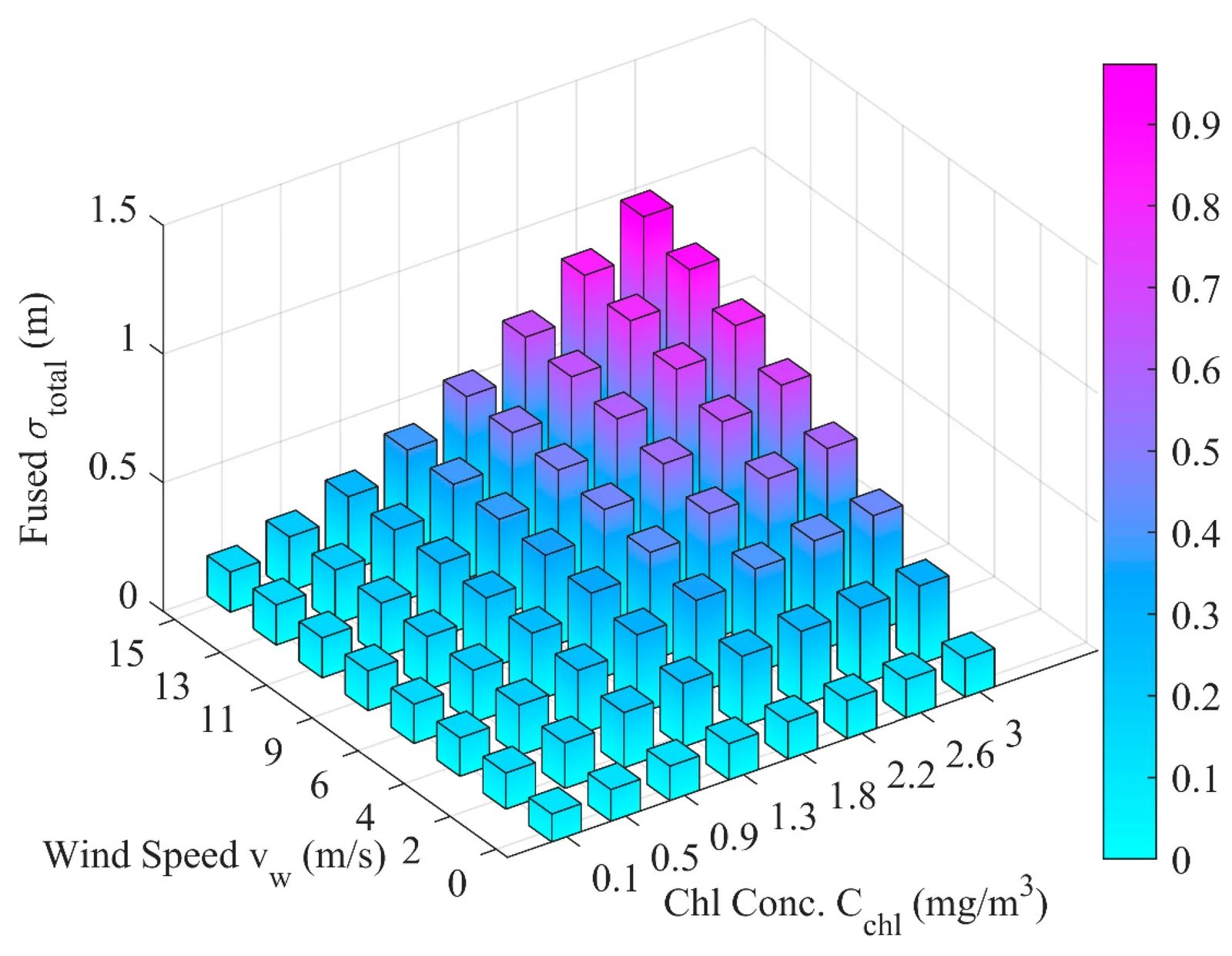
Analysis diagram of influencing factors on acoustic–optical joint ranging variance.

At a fixed communication distance of d = 50 m, Figure 11 shows the total fused ranging standard deviation $\sigma_{total}$ under varying wind speeds and turbidity. The heterogeneous fusion system remains in a low-error state as long as either the acoustic or optical channel remains reliable. For example, under high wind speeds with clear water or high turbidity without wind, the fusion standard deviation remains below 0.5 m, indicating that approximately 75% of the environmental conditions maintain high ranging accuracy through acoustic–optical complementarity. Only in the dual-harsh region with wind speed > 12 m/s and $C_{chl}>2.5\;mg/m^{3}$ does the error exceed 1.0 m. Compared with a single channel, the available operating range of the fusion system is improved by approximately 40%.

Figure 11 verifies the complementary effect of the MVUE-based acoustic–optical fusion mechanism and further motivates the use of GP-AC to handle the residual localization uncertainty under dual-harsh conditions. By perceiving the variance changes induced by wind speed and turbidity in real time, the MVUE mechanism dynamically reallocates confidence weights and suppresses severely degraded observations. For the residual high-dimensional uncertainty in the dual-harsh region, GP-AC further quantifies environmental uncertainty through GP regression and provides exploration compensation for the agent, thereby reducing blind exploration and local stagnation under extremely harsh sea states.

(4) Validity Verification and Analysis of the Environment-Aware Adaptive Fusion Model

To rigorously verify the superiority of the Environment-Aware Adaptive Linear Weighted Fusion (EA-ALWF) model proposed in Section 4 for processing underwater non-stationary heterogeneous data, a comparative experimental framework based on Monte Carlo simulation is constructed in this section. The experiment is designed to demonstrate that in complex underwater environments, ranging accuracy and robustness can be significantly improved by explicitly introducing environmental variance terms into the modeling (i.e., considering uncertainty), compared to simplified models where environmental impacts are ignored.

1. Definition of Experimental Variables and Construction of Control Groups

According to the propagation models derived in Section 4, the ranging strategies are classified into two categories: “environmental variance unconsidered” and “environmental variance considered”. The experimental conditions are set to mixed harsh sea states (high wind speed and high turbidity).

Strategy I: Fixed-weight baseline group (${\tilde{z}}_{1}$) ${\tilde{z}}_{1}=\lambda_{1}^{\prime }z_{ac}+\lambda_{2}^{\prime }z_{op}$

The basic observation data $z_{ac},\;z_{op}$ (without superimposed environmental variance terms) are utilized by this strategy, and empirically fixed weight coefficients are adopted. This is considered the most basic control group. This strategy neither perceives environmental changes nor adjusts the weights. Due to the erroneous assignment of an excessively high fixed weight (0.7) to the optical channel, the maximum error is induced at long distances as a result of the deterioration of the optical SNR. This is regarded as the worst-case performance baseline.

Strategy II: Basic adaptive control group (${\tilde{z}}_{2}$) ${\tilde{z}}_{2}=\lambda_{1}z_{ac}+\lambda_{2}z_{op}$

Although adaptive weights are adopted by this strategy, its inputs and weight calculations are solely based on the basic data $z_{ac},\;z_{op}$ (i.e., the environmental variances $\sigma_{ac},\;\sigma_{op}$ are ignored). The water body is assumed to be ideally clear, and the variance is calculated solely based on the communication distance. Due to the neglect of environmental uncertainty, the optical error in the actual environment is severely underestimated by this strategy. Therefore, the optical weight $\lambda_{2}$ calculated by this strategy is significantly higher than its appropriate value. This model–environment mismatch prevents complete suppression of environmental noise, resulting in suboptimal accuracy.

Strategy III: Environment-aware adaptive experimental group ($d_{fuse}$) $d_{fuse}=\lambda_{1}{\tilde{z}}_{ac}+\lambda_{2}{\tilde{z}}_{op}$

This strategy is considered the core contribution of this paper. The real data ${\tilde{z}}_{ac},\;{\tilde{z}}_{op}$ containing environmental variances are directly processed by it, and the environment perception module from Section 4 is utilized to accurately calculate the total variance encompassing the impacts of wind speed and turbidity; the optimal weights are allocated accordingly.

The superiority of $d_{fuse}$ lies in the precise modeling of uncertainty. Because environmental variances are explicitly introduced, the algorithm can perceive the current water turbidity; consequently, the calculated optical weight $\lambda_{2}$ is substantially reduced to a very low value. By this mechanism, environmental interference can be maximally suppressed by $d_{fuse}$, whereby the lowest ranging error is obtained.

2. Analysis of the Impact of Communication Distance on Ranging Accuracy

**Figure 12:**
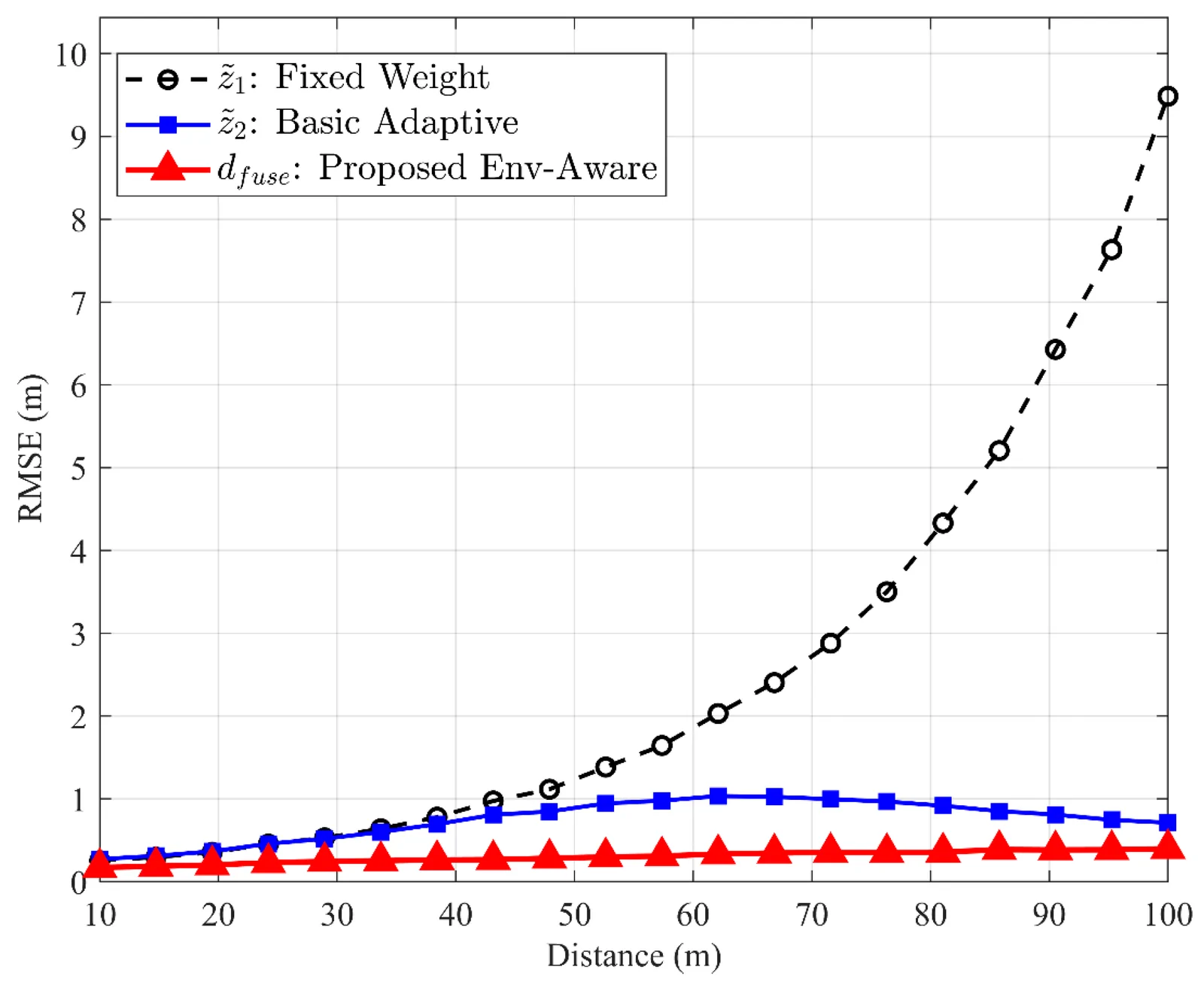
Comparison diagram of ranging RMSE varying with communication distance under different fusion strategies.

Figure 12 shows the RMSE variation of three fusion strategies with communication distance $d\in \left[10,100\right]\;m$ under a typical high-turbidity condition of $C_{chl}=2.0\;mg/m^{3}$, allowing their robustness to environmental non-stationarity to be quantitatively compared from the near field (10 m) to the far field (100 m).

The fixed-weight baseline maintains an RMSE below 1.0 m at d < 30 m, but increases rapidly with distance and reaches approximately 9.15 m at d = 100 m. This is mainly caused by exponential optical attenuation in turbid water. Although the optical SNR approaches zero at long distances, the fixed-weight strategy still assigns a confidence level of 0.7 to the optical link, causing the fused estimate to be dominated by environmental noise.

The basic adaptive method avoids nonlinear divergence, but its RMSE still fluctuates over the full distance range, peaking at approximately 1.05 m near d = 60 m and converging to approximately 0.68 m at d = 100 m. This is because the distance-dependent variance model underestimates abrupt turbidity-induced optical noise, leaving a model-mismatch error of approximately 0.3–0.6 m.

In contrast, the environment-aware adaptive method maintains the RMSE below 0.40 m across the entire distance range. At d = 100 m, it reduces the error from 9.15 m to 0.38 m relative to the fixed-weight baseline, corresponding to an improvement of 95.8%. Compared with the basic adaptive estimate of 0.68 m, the fused estimate is further reduced to 0.38 m, yielding an additional improvement of 44.1%. These results indicate that geometric distance adaptation alone is insufficient under harsh underwater conditions, whereas explicit environmental-variance perception is necessary to achieve robust and near-optimal ranging accuracy.

3. Analysis of Statistical Stability of Error Distribution and Outlier Suppression Characteristics

**Figure 13:**
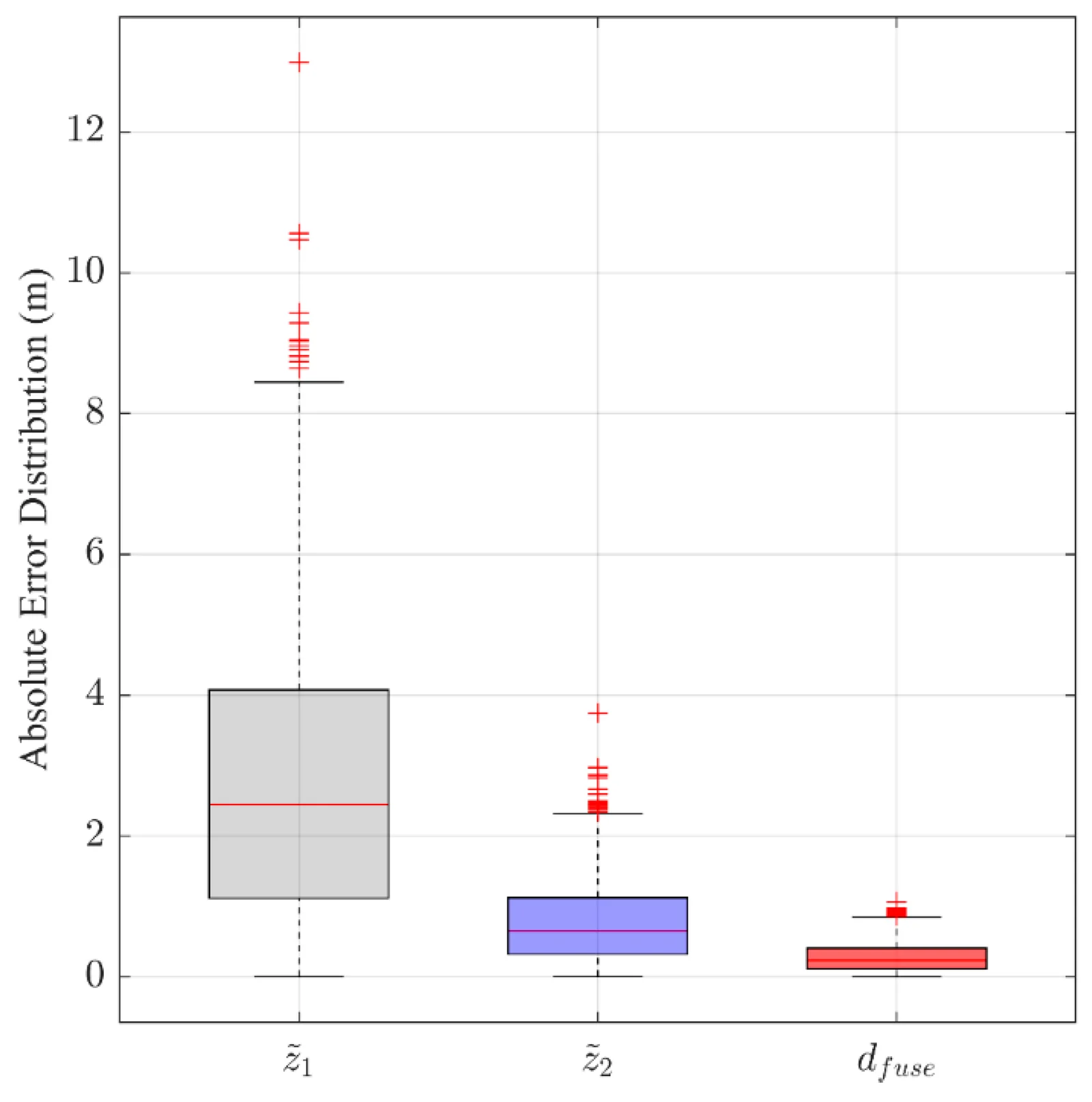
Results of statistical stability of ranging error distribution under typical far-field working conditions. The “+” symbols represent outliers, and the red horizontal lines inside the boxes indicate the median values.

Figure 13 presents the error distributions of the three strategies under a typical far-field condition of d = 80 m, based on 100 random Monte Carlo experiments. For comparative analysis, the fixed-weight baseline (${\tilde{z}}_{1}$), distance-oriented adaptive method (${\tilde{z}}_{2}$), and proposed environment-aware adaptive fusion method ($d_{fuse}$) are represented by black, blue, and red boxplots, respectively.

The fixed-weight baseline exhibits the widest error distribution and poorest statistical stability, with a median absolute error of approximately 2.4 m, an interquartile range of about 1.1–4.1 m, an upper whisker of approximately 8.4 m, and a maximum outlier approaching 13 m. The basic adaptive method reduces the median error to approximately 0.6 m and narrows the interquartile range to about 0.3 m; however, its upper whisker still reaches approximately 2.3 m, with the largest outlier close to 3.7 m, indicating that distance-dependent weighting alone cannot fully suppress unmodeled environmental variations. In contrast, the proposed environment-aware adaptive fusion method yields the most compact distribution, with a median error of approximately 0.2 m, most errors concentrated between about 0.1 m and 0.35 m, and the remaining outliers limited to approximately 1.0 m. These results show that incorporating environment-dependent ranging variances into the weighting process substantially reduces the median error, dispersion, and upper-tail risk, thereby improving the statistical stability and robustness of heterogeneous ranging fusion.

The representative acoustic, optical, and fused ranging standard deviations under four environmental scenarios are summarized in Table 4, demonstrating the complementary behavior of the two ranging modalities and the stability of the adaptive fusion mechanism. The single-channel results show distinctly different error-growth patterns. For acoustic ranging, when sea-surface wind speed increases from 2 m/s to 14 m/s, the standard deviation rises gradually from 0.30 m to 0.90 m, indicating bounded degradation with sea-state variation. In contrast, the optical channel exhibits pronounced nonlinear degradation: under clear water with $C_{chl}=0.5\;mg/m^{3}$, its standard deviation is only 0.05 m, whereas it exceeds 4.50 m when turbidity increases to $C_{chl}=2.5\;mg/m^{3}$ under an algal-bloom condition. The fused results confirm the acoustic–optical complementarity. In Scenario II with acoustic interference and Scenario III with severe optical degradation, the adaptive fusion standard deviations remain below 0.15 m and around 0.32 m, respectively, showing that sub-meter ranging accuracy can be maintained when at least one modality remains reliable. Under the dual-harsh condition of Scenario IV, the fused standard deviation increases to approximately 1.00 m, but remains substantially lower than the optical error of more than 4.50 m. This indicates that the MVUE mechanism reallocates confidence toward the less degraded acoustic observation, maintaining bounded ranging performance even when both modalities deteriorate.

### 6.3. Verification of the Optimization Framework

#### 6.3.1. Verification of Localization Performance

In the previous section, it was verified that high-quality distance inputs can be provided for the localization system by the environment-aware heterogeneous ranging model. In this section, the performance of the continuous space optimization model based on GP-AC proposed in Section 5 in solving underwater node coordinates will be primarily verified.

To comprehensively evaluate the stability and accuracy of the algorithm in solving high-dimensional, non-linear underwater localization problems, 200 independent Monte Carlo experiments were conducted within a 3D underwater monitoring area of 100 m×100 m×100 m. In the experiments, the number of anchor nodes was set to 8, the maximum number of exploration steps for a single optimization by the agent was set to $T_{max}=100$, and the Markov discount factor was set to γ=0.95.

1. Statistical Analysis of Localization Accuracy and Execution Time

The localization RMSE values obtained from the 200 independent runs were statistically summarized, and the results are presented in Table 5.

The localization results from 200 independent runs are summarized in Table 5. Hybrid acoustic–optical localization has been investigated for underwater sensor nodes [50], while practical underwater localization trials have reported a trajectory RMS positioning error of approximately 1.36 m [51]. Based on this reported engineering accuracy level, 1.36 m is adopted in this study as a literature-informed engineering reference error boundary. The proposed model achieves an average RMSE of 0.1420 m, substantially below this boundary, with optimal and worst values of 0.0632 m and 0.2854 m, respectively; even under severe noise and unfavorable random initialization, the error remains below 0.29 m. The standard deviation is only 0.0510 m, demonstrating stable convergence across different random anchor-node topologies. These results indicate that geometric topology perception and the Geometric Topology Multi-Stage Reward (GTM-Reward) mechanism effectively reduce premature convergence and localization failure under underwater multipath and coplanar topological blind zones. The average execution time for a single localization calculation is 7.45 s, mainly due to recursive GPTD posterior-covariance updates, the solution of the damped Fisher linear system, and the inversion of the regularized geometric information matrix for GDOP evaluation. Although these operations increase computational complexity, they improve localization accuracy from the meter level (>1.0 m) to the decimeter level (approximately 0.14 m). Since UWSNs are commonly used for long-term ocean monitoring and the considered nodes are quasi-static, a 7.45 s localization time provides a reasonable tradeoff between computational cost and high-reliability localization under complex sea conditions.

2. Representative Single-Run Localization Result

**Figure 14:**
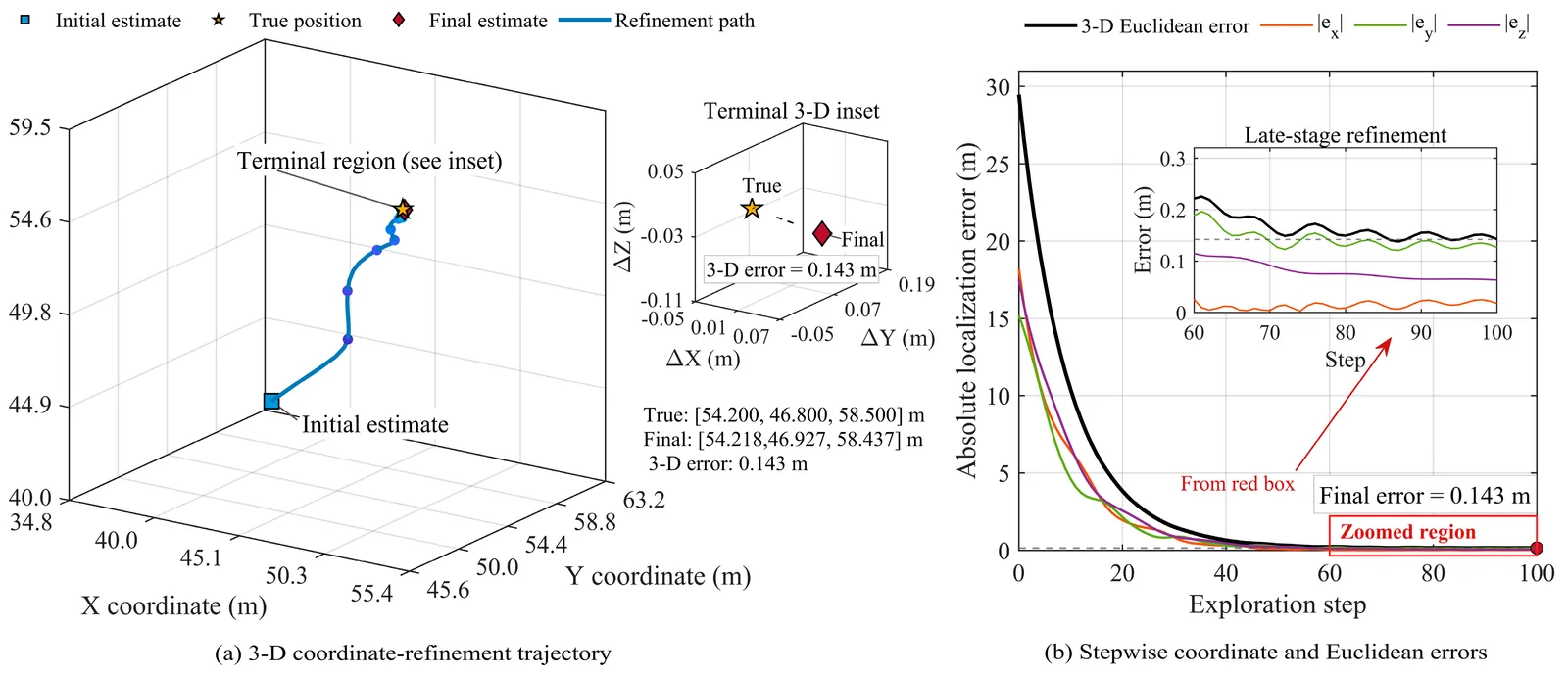
Representative single-run localization trajectory and error evolution of the proposed GP-AC method: (**a**) three-dimensional coordinate-refinement trajectory; (**b**) stepwise coordinate and Euclidean localization errors. The blue circles denote intermediate position estimates during the iterative refinement process, while the dashed lines are used as auxiliary guides to indicate the corresponding region and do not represent any additional physical quantity.

To complement the ensemble-level statistical evaluation presented in Table 5 and provide a more direct illustration of the obtained coordinate-level localization result, a representative single-run experiment is presented in Figure 14. The experiment was conducted using the same configuration as that adopted in the preceding statistical evaluation, including eight anchor nodes, a maximum exploration budget of 100 steps, and a discount factor of 0.95. The true coordinate of the unknown node was set to [54.200, 46.800, 58.500]^T^ m, while the initial candidate coordinate was [36.000, 62.000, 41.000]^T^ m.

As shown in Figure 14a, the candidate coordinate gradually moves from the initial estimate toward the true position. Relatively large coordinate updates occur during the early exploration stage, indicating that the agent first performs a broader search in the coordinate space. After the candidate position enters the terminal region, the update magnitude decreases and the trajectory becomes more concentrated around the final solution. The enlarged three-dimensional inset further illustrates the spatial relationship between the true position and the final estimate. The final estimated coordinate is [54.218, 46.927, 58.437]^T^ m, corresponding to a three-dimensional Euclidean localization error of approximately 0.143 m. Figure 14b further shows the evolution of the absolute errors along the *x*-, *y*-, and *z*-axes, together with the three-dimensional Euclidean error. The errors decrease rapidly during the early exploration stage and then gradually enter a comparatively stable decimeter-level range. The enlarged interval from Steps 60 to 100 shows small bounded variations during the late-stage refinement, suggesting that limited local adjustments are still performed near the estimated position. Overall, this representative run provides a direct illustration of the coordinate-refinement process and complements the statistical evaluation in Table 5. Its final error is also broadly consistent with the average RMSE of 0.142 m obtained from the 200 independent runs.

3. Convergence Behavior and Mechanism Analysis

**Figure 15:**
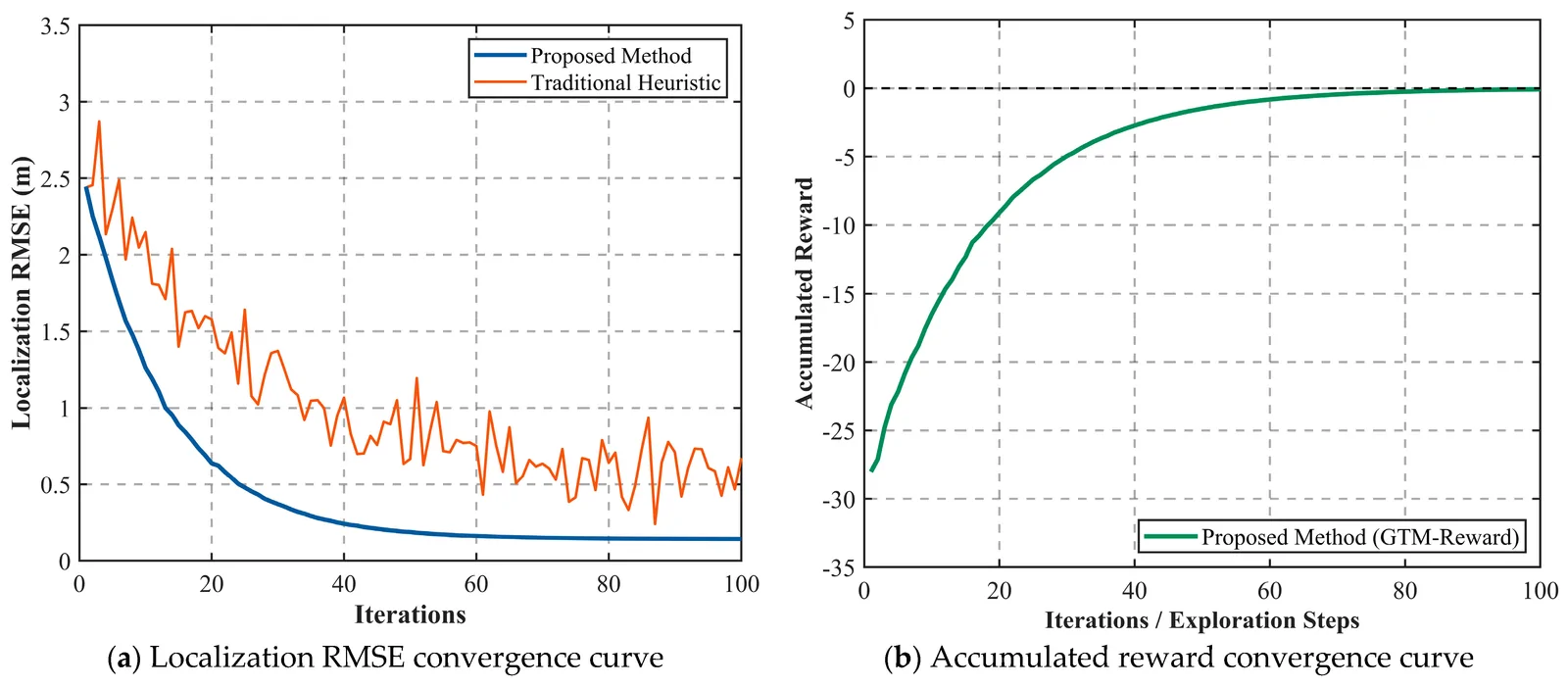
Convergence characteristic curves of RMSE and reward function of the proposed model.

Figure 15 presents the convergence of the proposed GP-AC method with eight anchor nodes, a maximum of 100 exploration steps, and a discount factor of 0.95; the remaining parameters are listed in Table 2. Figure 15a shows that the localization RMSE decreases rapidly during the early iterations and then stabilizes at approximately 0.142 m. Figure 15b shows that the reward rises from about −28 and gradually reaches a stable level. According to Equation (72), the reward is the negative weighted combination of the algebraic localization-error penalty, the GDOP-based geometric topology penalty, and the boundary penalty; therefore, a larger reward corresponds to a lower overall localization cost.

The two curves exhibit a consistent relationship: the reward increases as the RMSE decreases. In the early stage, large ranging residuals produce a low reward, while GP uncertainty-guided exploration rapidly reduces the coordinate error. During the middle and late stages, the GDOP-based term guides the agent toward geometrically better-conditioned regions, causing both curves to approach stable plateaus. This behavior indicates that the ranging, geometric topology, and boundary penalties are progressively reduced. Under the adopted configuration, the method reaches a stable solution within the 100-step exploration budget and achieves an RMSE of approximately 0.142 m. Noise-induced degradation is evaluated separately in Section 6.3.3 through repeated Monte Carlo experiments under different non-Gaussian contamination ratios.

#### 6.3.2. Analysis of Parameter Sensitivity and Robustness Boundaries

To evaluate the engineering applicability of the localization system in complex hydrological environments, Local Sensitivity Analysis (LSA) is employed to investigate the effects of environmental parameters and system configurations on localization accuracy. Both random observation-noise factors, including wind speed and turbidity, and sound-speed mismatch factors, including temperature and salinity, are considered to characterize the performance boundaries of the algorithm.

(1) Comparative Analysis of the Sensitivity of Multi-Source Environmental Factors

**Figure 16:**
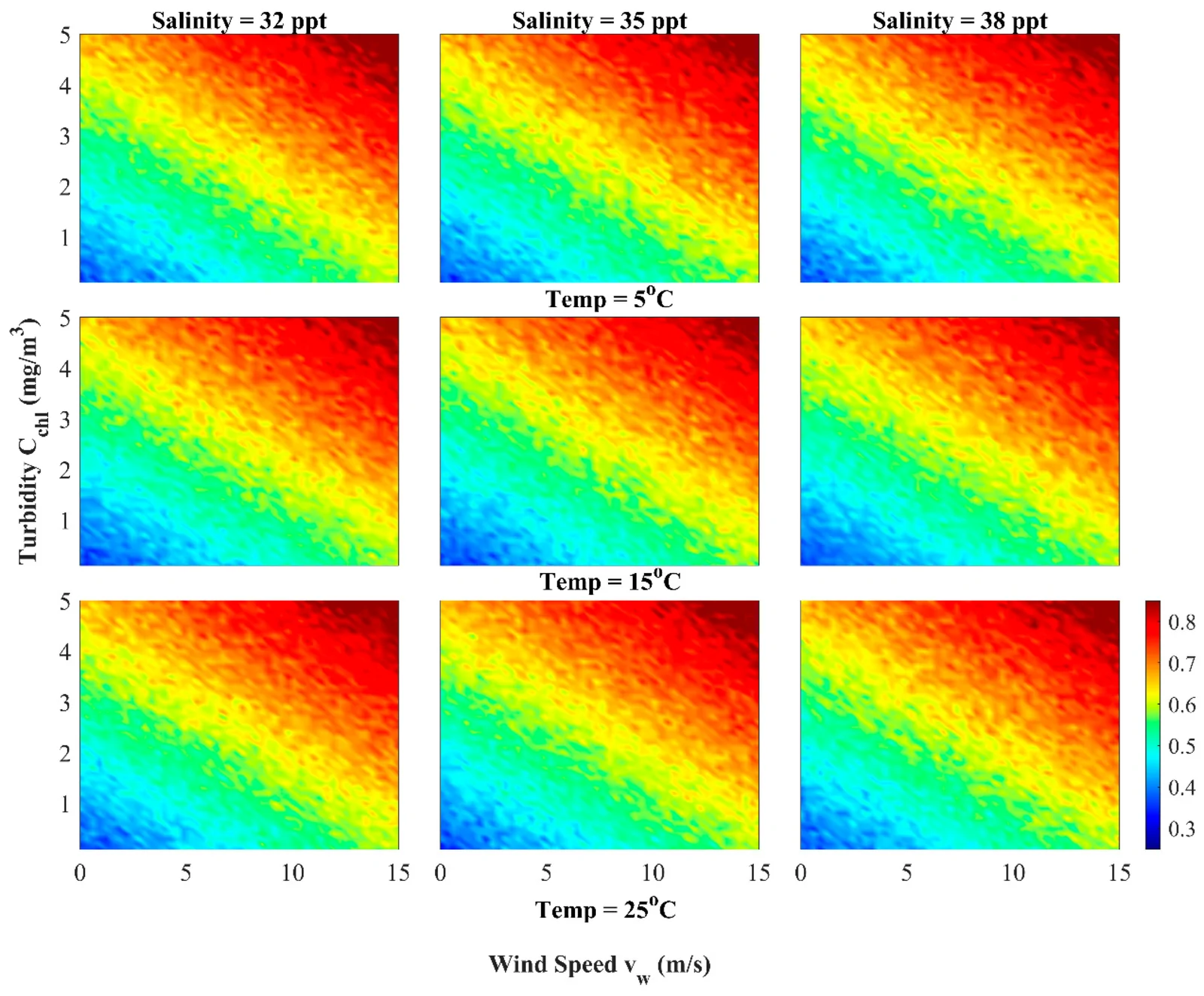
Comparative sensitivity analysis of multi-source environmental perturbation factors on localization accuracy.

To evaluate the global stability of the proposed model under complex hydrological conditions, a four-dimensional environmental stress test involving wind speed ($v_{w}$), turbidity ($C_{chl}$), temperature (T), and salinity (S) is conducted using faceted heatmaps. Figure 16 presents a 3 × 3 RMSE heatmap matrix, with three temperature levels as rows and three salinity levels as columns. Each subplot shows localization RMSE versus wind speed and turbidity under a specified temperature–salinity condition, thereby revealing both random observation disturbances and systematic sound-speed deviations.

1. Suppression of Random Observation Noise

Within each subplot, such as the central panel with T = 15 °C, S = 35 ppt, the localization error increases gradually from the lower-left region, representing low wind speed and low turbidity, to the upper-right region, representing high wind speed and high turbidity. The color changes from dark blue to cyan-yellow, while no dark-red failure region appears. This moderate and continuous increase indicates that the proposed method can suppress observation noise induced by wind waves and optical scattering without producing abrupt localization failure. The main reason is that the environment-aware weighting mechanism evaluates channel quality and reduces the weights assigned to degraded acoustic or optical links.

2. Adaptation to Systematic Sound-Speed Deviations

Across the complete 3 × 3 matrix, the color distribution patterns remain highly consistent. Even when the temperature increases from 5 °C to 25 °C, or when salinity changes, no significant RMSE shift is observed at the same wind-speed and turbidity coordinates. This result indicates that the proposed GP-AC model maintains strong adaptability to systematic deviations caused by sound-speed-profile variations. Although temperature and salinity changes alter the sound-speed profile and introduce systematic ranging deviations, their nonlinear effects on the value function are captured by the GP Critic, while the NPG Actor performs refined manifold updates in the continuous non-convex solution space. Consequently, model mismatch is adaptively compensated during coordinate updates, and the localization result remains comparatively stable across different temperature–salinity combinations.

Overall, Figure 16 shows that the proposed GP-AC method remains effective across multiple environmental combinations rather than under a single experimental condition, demonstrating stable localization performance under highly variable simulated marine environments.

(2) Sensitivity Analysis of System Configuration and Algorithm Parameters

In addition to the external environment, internal hardware deployment (the number of anchor nodes) and algorithm settings (the maximum number of exploration steps) are similarly considered key factors affecting system performance.

1. Impact of the Number of Anchor Nodes

**Figure 17:**
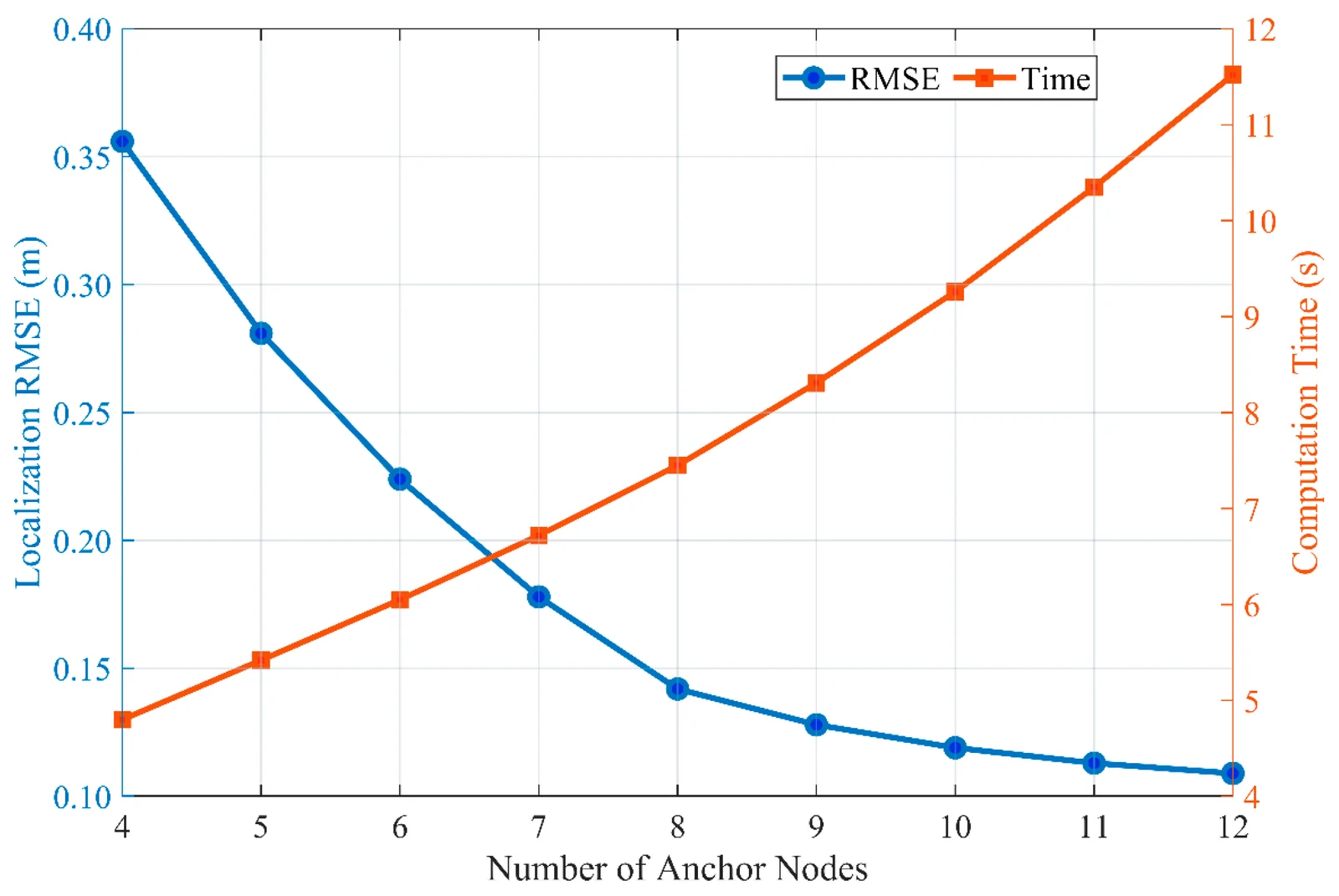
Effect of the number of anchor nodes on localization RMSE and computation time.

The dual-axis curves in Figure 17 illustrate the variation in localization RMSE and computation time as the number of anchor nodes increases from 4 to 12. As the number of anchors increases, the RMSE decreases monotonically from approximately 0.356 m at four anchors to 0.142 m at eight anchors and 0.109 m at twelve anchors. This improvement results from additional geometric constraints, which reduce the GDOP and improve the observability of the three-dimensional coordinates. Meanwhile, the computation time increases from approximately 4.8 s to 7.45 s and 11.5 s, respectively, because additional ranging residuals and geometric terms must be evaluated. The RMSE decreases rapidly before reaching eight anchors, whereas the improvement becomes marginal beyond eight anchors, while the computation time continues to increase. Therefore, eight anchor nodes are selected as a favorable configuration for balancing localization accuracy and computational efficiency.

2. Impact of the Number of Steps on Performance and Efficiency

**Figure 18:**
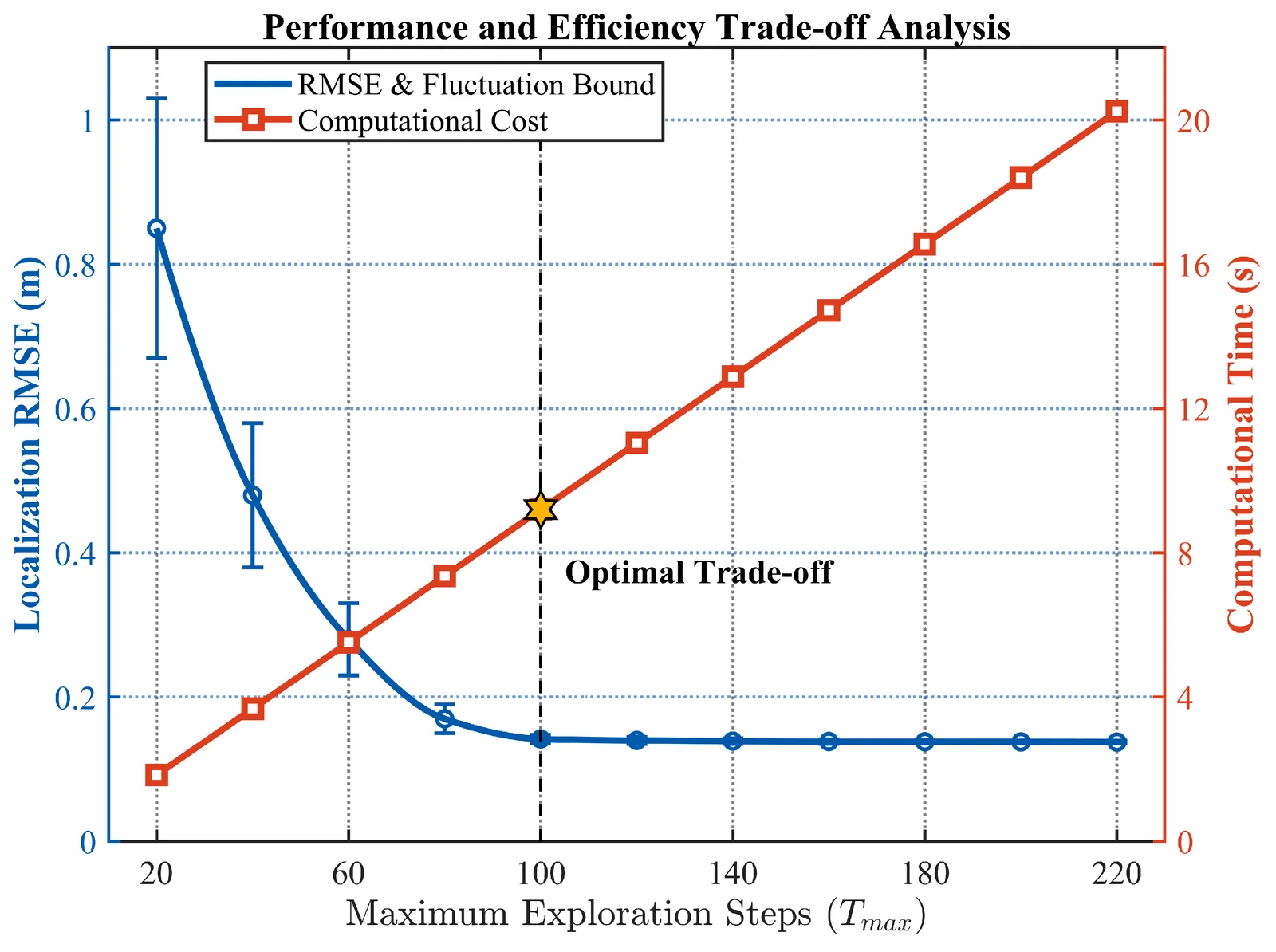
Trade-off between the maximum number of exploration steps and localization performance and computational efficiency. The star marks the selected optimal trade-off point, while the dashed and dot-dash lines serve as auxiliary reference lines.

The maximum exploration steps for a single localization calculation, $T_{max}$, is a key GP-AC hyperparameter affecting both localization accuracy and computation time. As shown in Figure 18, $T_{max}$ determines the sampling coverage of the unknown solution space and thus influences the exploration of the non-convex error surface. At $T_{max}=40$, the search space is insufficiently explored and the GP Critic dictionary remains sparse, resulting in premature convergence, noticeable fluctuations, and an average RMSE above 0.45 m. Increasing $T_{max}$ to 100 enables sufficient exploration and the transition from global search to local topology exploitation, reducing the RMSE to approximately 0.142 m. When $T_{max}$ is further increased to 200, the RMSE decreases only slightly to approximately 0.138 m, indicating diminishing accuracy gains.

The computation time increases approximately linearly with $T_{max}$ because a larger exploration budget proportionally increases the online inference burden. To balance accuracy and computational cost while keeping the single-localization delay below 10 s, $T_{max}=100$ is selected as the most cost-effective configuration. This setting provides sufficient Gaussian-space sampling to avoid topological blind zones while maintaining a favorable balance among localization accuracy, convergence reliability, and computational timeliness.

#### 6.3.3. Robustness Verification Under Non-Gaussian Noise Contamination

To examine whether the reported performance depends excessively on the Gaussian uncertainty model adopted in the simulations, an additional model-mismatch test was conducted under non-Gaussian noise. Drawing on the presentation approach adopted in [52], in which performance variations are characterized under controlled noise conditions using quantitative statistical indicators, the influence of non-Gaussian ranging noise was evaluated through repeated Monte Carlo trials and multiple error-distribution statistics. Specifically, a proportion ρ of the nominal Gaussian ranging error samples was replaced by zero-mean heavy-tailed Student’s t-distributed noise with three degrees of freedom, while the remaining samples followed the nominal Gaussian model. Thus, ρ denotes the proportion of ranging observations affected by non-Gaussian noise. The contamination ratio was set to 0%, 5%, 10%, 20%, and 40%, and 200 independent Monte Carlo trials were conducted for each condition. The GP-AC method continued to use the original uncertainty model without prior knowledge of the injected non-Gaussian noise. The results are presented in Table 6.

As shown in Table 6, the localization error increases gradually with the non-Gaussian noise contamination ratio. When ρ increases from 0% to 10%, the mean RMSE increases from 0.1420 m to 0.1796 m. Under the more severe 20% and 40% contamination conditions, it further increases to 0.2287 m and 0.3469 m, respectively, accompanied by increases in the standard deviation and 95th-percentile error. Nevertheless, even at ρ=40%, the maximum error remains 0.8427 m, below the literature-informed engineering reference error boundary of 1.36 m, and no error exceeding this reference boundary is observed in the 200 trials. These results indicate that the proposed method exhibits gradual degradation rather than abrupt divergence when the actual ranging error distribution deviates from the Gaussian model assumed by the uncertainty model.

#### 6.3.4. Ablation Experiments on Key Mechanisms

To separately quantify the contributions of the MVUE weighting mechanism, the RL-based coordinate optimizer, GP uncertainty compensation, the GDOP-based topology reward, and the NPG update, a controlled component-wise ablation study was conducted. Eight variants were constructed by progressively replacing or removing one component from the complete framework. Specifically, the WLS-only and MVUE + WLS variants were solved using the same conventional iterative solver, while the MVUE + WLS + RBFGS variant was introduced to evaluate adaptive weighting combined with a classical optimizer. The MVUE + Std-AC variant was used to examine the contribution of the RL optimizer without GP uncertainty, GDOP reward, or NPG. For the remaining three variants, only the indicated component was removed from the full GP-AC model, while all other modules were retained. All variants were evaluated using the same eight-anchor deployment, environmental realizations, initial coordinates, and stopping criteria. The maximum number of objective-function evaluations was fixed at 100, and each variant was independently executed 50 times.

As shown in Table 7, the localization accuracy improves progressively as the key components are introduced. MVUE weighting reduces the mean RMSE from 0.962 m to 0.728 m, while the RL optimizer further decreases it to 0.512 m. Among the single-component ablations, removing the GDOP reward causes the largest degradation, followed by the removal of the GP uncertainty bonus and NPG. The full GP-AC achieves the lowest mean RMSE of 0.142 m and the smallest standard deviation, confirming that MVUE weighting, uncertainty-guided exploration, topology-aware reward shaping, and NPG jointly contribute to the final performance.

### 6.4. Verification of the Proposed Algorithm’s Performance

#### 6.4.1. Comparison with Classical Nonlinear Estimation and Optimization Methods

To evaluate the relative advantage of reinforcement learning for the nonlinear coordinate-estimation problem considered in this study, EKF, UKF, PF, and RBFGS were selected as representative classical nonlinear estimation and optimization baselines. Since the original state and observation models of EKF, UKF, and PF differ from the acoustic–optical ranging model adopted here, they were consistently adapted to the present three-dimensional node-localization problem. The MVUE + WLS + RBFGS implementation used in the ablation study was retained to solve the same non-convex WLS objective under identical fused-ranging observations, initial coordinates, and computational budgets.

As shown in Table 8, GP-AC achieves the lowest mean RMSE of 0.142 m and the smallest standard deviation of 0.051 m. The mean RMSE values of EKF, UKF, PF, and RBFGS are 0.604 m, 0.492 m, 0.447 m, and 0.685 m, respectively. UKF outperforms EKF by avoiding direct first-order linearization, while PF provides improved nonlinear and non-Gaussian estimation capability at the cost of a longer computation time of 6.73 s. The relatively high error of RBFGS suggests that local quasi-Newton optimization remains sensitive to initialization and local structures of the non-convex error surface. Compared with EKF, UKF, PF, and RBFGS, GP-AC reduces the mean RMSE by approximately 76.5%, 71.1%, 68.2%, and 79.3%, respectively. Although EKF and UKF require less computation time, their error dispersion and 95th-percentile errors are less favorable, whereas PF remains affected by particle degeneracy and limited sample coverage. Overall, under the same acoustic–optical fused-ranging inputs and evaluation conditions, GP-AC provides a more favorable balance among localization accuracy, stability, and computational cost.

#### 6.4.2. Comprehensive Comparison of Localization Accuracy and Stability

To verify the advancement of the proposed GP-AC model in highly non-convex underwater localization problems, rigorous comparative experiments were conducted in this section between it and 10 state-of-the-art optimization algorithms. To ensure the comprehensiveness and fairness of the comparison, the baseline algorithms span beyond a single school of thought, covering three major technical categories together with one internal ablation baseline. Specifically, the Improved Sparrow Search Algorithm (ISSA) [53] and the Improved Whale Optimization Algorithm (IWOA) [54] were selected from the bio-inspired camp; the Riemannian Broyden-Fletcher-Goldfarb-Shanno (RBFGS) [55] and the Riemannian Stochastic Gradient Descent (RSGD) [56] were chosen from the Riemannian manifold gradient camp; 5 advanced baseline protocols customized specifically for UWSNs and complex time-varying channels in recent years (2022–2026) were selected from the RL camp. These include the mobile localization algorithm based on anchor node selection optimization (RMUL) [35], the asynchronous localization algorithm fusing scalable sampling (RL-PCM) [37], the multi-Unmanned Underwater Vehicle (multi-UUV) collaborative grid localization algorithm (RL-GLMU) [38], the efficient beacon-assisted localization algorithm (BA-RL) [39], and the shared-parameter multi-agent algorithm for resisting communication loss in non-stationary environments (SP-MARL) [57]. Furthermore, the Standard Actor-Critic (Std-AC) algorithm, which does not combine the GP and the local geometric potential energy term, was introduced as the basic ablation baseline.

To avoid disadvantaging any competing method through inappropriate parameter settings, the hyperparameters recommended in the corresponding original studies were used as the initial configurations for all baseline algorithms. Each baseline algorithm was then tuned independently using the same validation instances and an identical hyperparameter-search budget, and the configuration achieving the lowest average validation RMSE was adopted for the final comparison. Since the compared algorithms involve two fundamentally different optimization mechanisms, namely population-based parallel iteration and single-agent sequential decision-making, the maximum number of objective-function evaluations per run was uniformly limited to 100 for all algorithms to ensure computational parity. Under this common evaluation protocol, all algorithms were independently executed 50 times in the same 3D underwater simulation environment, and the reported results were obtained from these independent runs to reduce the statistical influence of random initialization.

**Figure 19:**
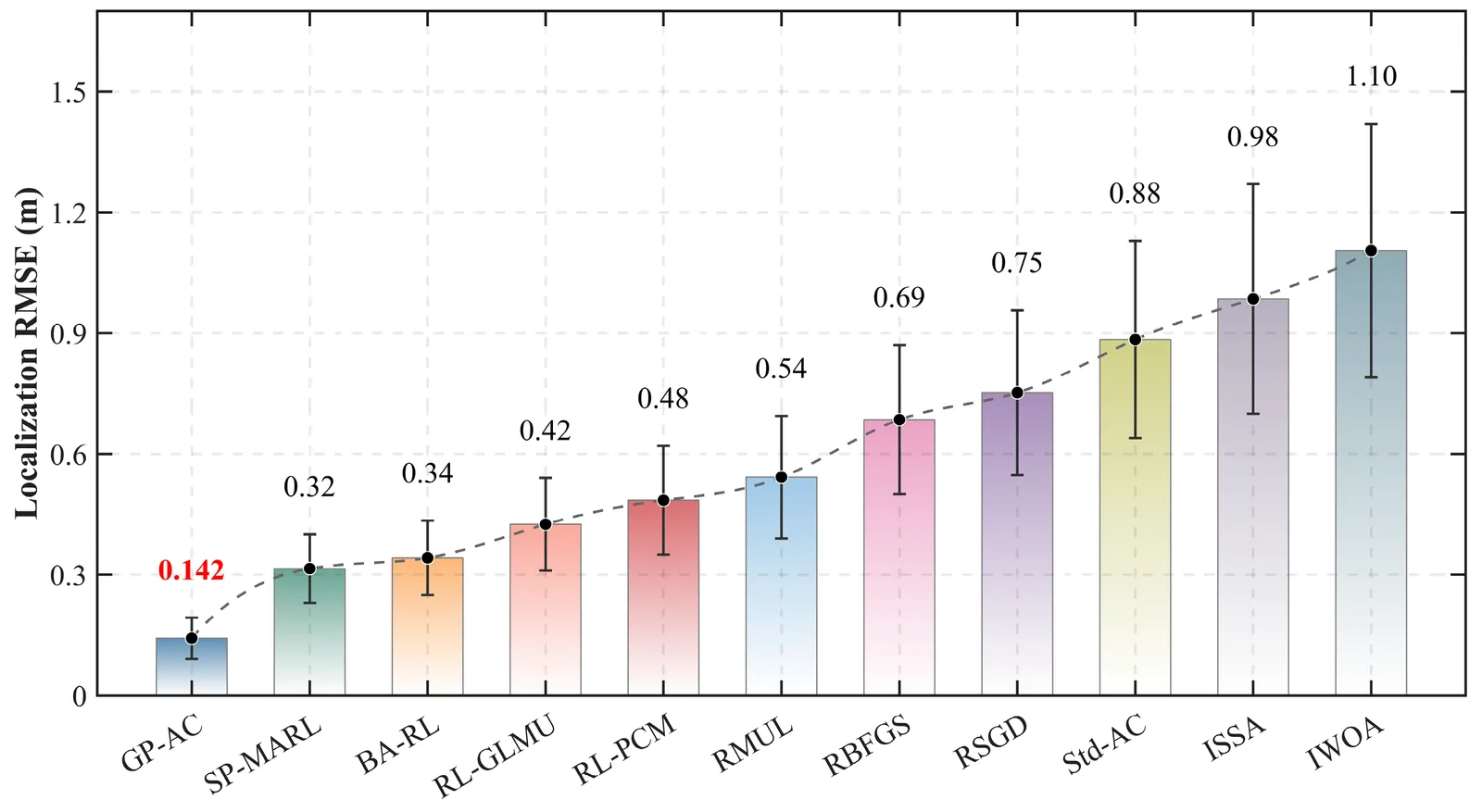
Statistical comparison of localization RMSE between the proposed algorithm and other algorithms. The red number highlights the localization RMSE of the proposed GP-AC method.

Figure 19 compares the RMSE statistics of GP-AC with 10 state-of-the-art algorithms under the same evaluation protocol. GP-AC achieves the lowest average RMSE of 0.142 m with a very small standard deviation, demonstrating high localization accuracy and robustness to underwater multipath noise and node-topology variations. Compared with the RL-based SP-MARL (RMSE = 0.315 m) and BA-RL (RMSE = 0.342 m), GP-AC improves localization accuracy by 54.9% and 58.5%, respectively. Unlike these methods, GP-AC incorporates local GDOP into a multi-stage dynamic reward, guiding the agent toward better-conditioned regions and avoiding unfavorable geometric layouts. GP-AC also outperforms RBFGS (RMSE = 0.685 m) and Std-AC (RMSE = 0.884 m), with improvements of 79.3% and 83.9%, respectively. This advantage mainly arises from GP-based epistemic uncertainty estimation and upper-confidence exploration, which enhance exploration in insufficiently sampled regions and reduce local stagnation and blind trial-and-error. Compared with the swarm-intelligence methods ISSA (RMSE = 0.985 m) and IWOA (RMSE = 1.105 m), GP-AC reduces the error by up to 87.1%. Under the same budget of 100 objective-function evaluations per run, population-based methods must distribute evaluations among multiple candidates, whereas GP-AC uses sequential policy-guided coordinate refinement to improve sample utilization and smoothly transition from global exploration to local exploitation. Overall, the superiority shown in Figure 19 results from the combined improvements in geometric guidance, uncertainty-aware exploration, environmental state evaluation, and interaction-signal design.

#### 6.4.3. Analysis of Cumulative Error Distribution Characteristics

**Figure 20:**
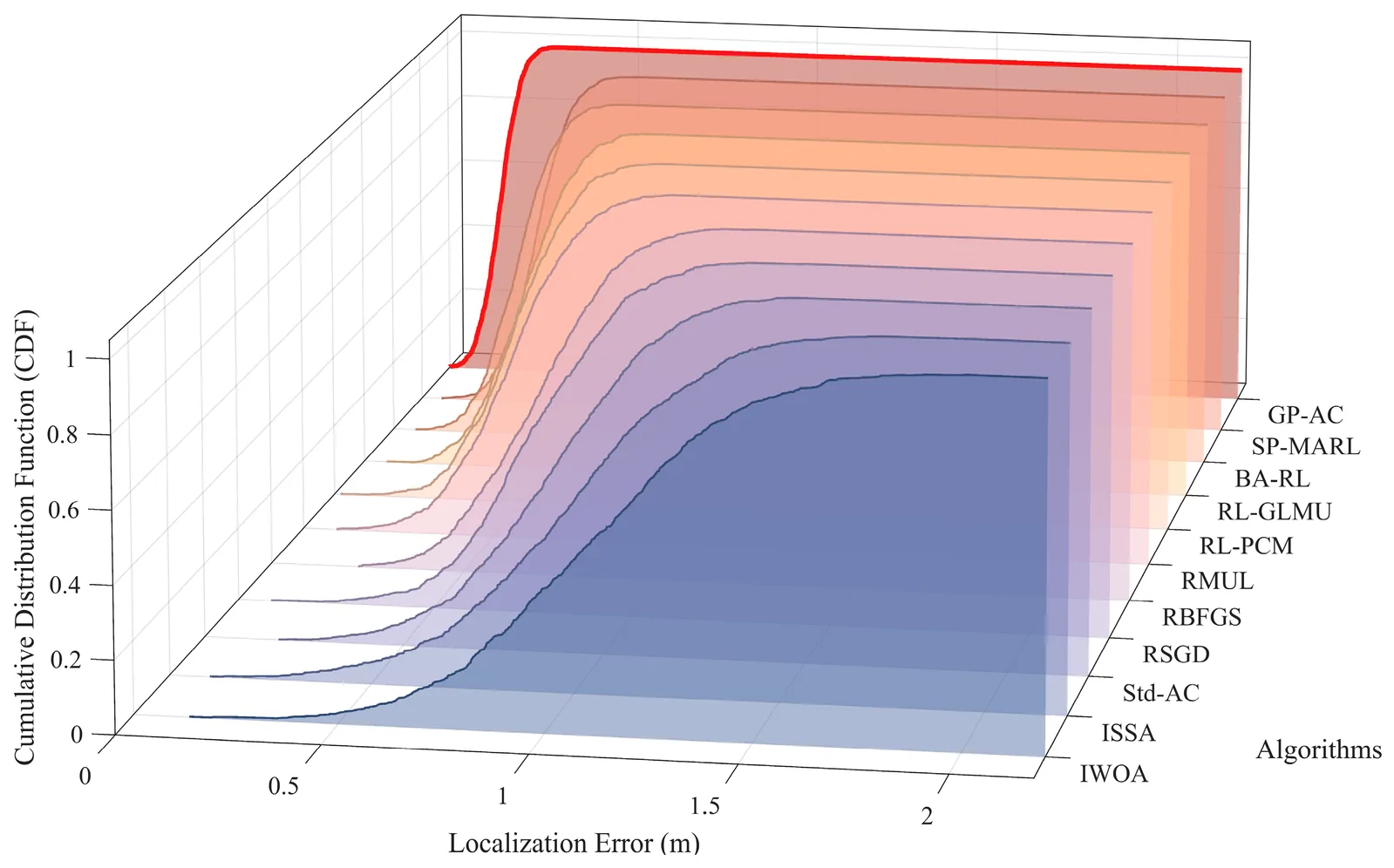
Comparison of cumulative distribution function curves of localization error for different algorithms. The red line highlights the CDF curve of the proposed GP-AC method, while the other colors are used to distinguish the different comparison algorithms.

Figure 20 presents the CDF curves of localization errors for GP-AC and the compared algorithms, reflecting error concentration, convergence consistency, and upper-tail behavior. GP-AC shows the steepest rise and most concentrated distribution, with more than 95% of errors below 0.25 m and the cumulative probability rapidly approaching 1.0. By comparison, SP-MARL and BA-RL approach saturation at approximately 0.45–0.50 m, while RBFGS and IWOA exhibit flatter long-tail distributions, with some residual errors exceeding 1.5 m.

RSGD, RBFGS, and Std-AC are more susceptible to saddle-point stagnation and insufficient exploration on highly non-convex localization surfaces. GP-AC alleviates this problem by using GP posterior variance as an uncertainty-guided exploration bonus, making its cumulative convergence probability below 0.3 m nearly 80% higher than that of Std-AC. For SP-MARL, BA-RL, RL-GLMU, RL-PCM, and RMUL, reward functions dominated by algebraic ranging residuals may lead to topological traps under coplanar or poorly conditioned geometries. At 0.3 m, their CDF values remain near 40%, whereas GP-AC reaches 100%. By incorporating local GDOP into a multi-stage dynamic penalty, GP-AC improves geometric robustness and convergence consistency.

ISSA and IWOA distribute limited evaluations among multiple candidates, reducing local refinement efficiency. In contrast, GP-AC uses sequential policy-guided coordinate refinement to transition from global exploration to local exploitation. Under the same computational budget, GP-AC achieves solutions that are up to 87.1% better than those of the swarm-intelligence methods. Overall, uncertainty-guided exploration and geometric topology-aware optimization provide GP-AC with higher localization reliability and stronger environmental adaptability.

#### 6.4.4. Analysis of Localization Time Cost and Efficiency

**Figure 21:**
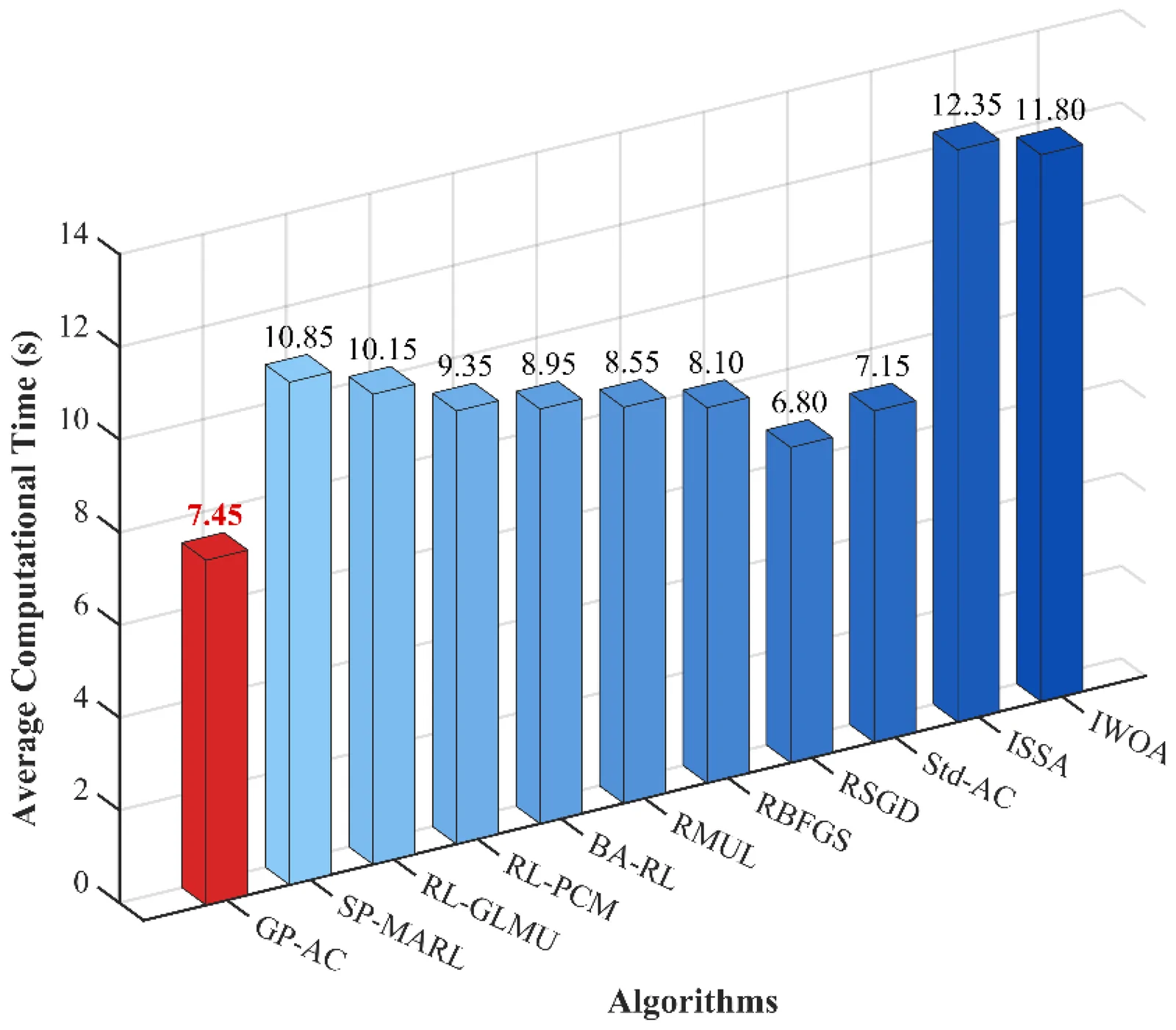
Comparison of average single localization calculation time consumption among different improved algorithms. The red bar and red number highlight the computational time of the proposed GP-AC method, while the other colors are used only to distinguish the comparison algorithms.

Figure 21 compares the average time required for a single localization calculation by GP-AC and 10 competing algorithms under the same hardware environment. GP-AC requires 7.45 s despite incorporating GP-based uncertainty estimation, GDOP evaluation, and a multi-stage reward mechanism.

The swarm intelligence and complex multi-agent methods, including ISSA, IWOA, SP-MARL, and RL-GLMU, require approximately 9.6–12.3 s. Compared with ISSA (12.35 s) and SP-MARL (10.85 s), GP-AC reduces the calculation time by 39.7% and 31.3%, respectively. This advantage mainly results from its single-agent sequential architecture, which avoids repeated population-wide evaluations, communication synchronization, and parameter sharing. The basic underwater RL and manifold methods, including RL-PCM, BA-RL, RMUL, and RBFGS, require approximately 8.1–9.3 s, making GP-AC about 15.2% faster on average. This indicates that the additional GP and GDOP operations do not offset the efficiency gained from end-to-end single-agent inference. RSGD and Std-AC are the fastest baselines, at 6.80 s and 7.15 s, respectively. GP-AC is slower by 9.6% and 4.2% because of GPTD covariance updates and GTM-Reward geometric state evaluation, but the additional cost remains below 0.65 s on average. In contrast, RSGD and Std-AC exhibit substantially higher RMSE values of approximately 0.75 m and 0.88 m.

Overall, GP-AC maintains a single-localization delay of 7.45 s while achieving decimeter-level accuracy, providing a favorable balance among computational cost, convergence quality, and environmental robustness.

## 7. Conclusions

Aiming at the problem that sensor ranging errors are difficult to quantify in complex underwater environments, a multi-stage RL strategy enhanced by geometric topology perception is proposed in this paper. First, an uncertainty quantification model under multi-source interference is established to characterize the time-varying noise distribution, whereby the effective isolation of harsh observation data and the characterization of error variance are achieved. Secondly, taking the aforementioned variance as input, an adaptive weighting mechanism based on MVUE is constructed, and the robust fusion of multimodal observation data is realized. Finally, an objective function is constructed based on the fused distance, and the GP-AC algorithm is designed for global optimization, whereby the 3D spatial coordinates of unknown nodes are obtained. Simulation and comparative experiments demonstrate that the proposed method achieves substantial improvements across multiple key indicators. In particular, the proposed environment perception and robust fusion model effectively mitigates the impact of extreme sea-state interference, resulting in a 91.9% relative improvement in the system’s anti-interference robustness under harsh environmental conditions. The constructed 3D localization optimization framework improves coordinate-estimation capability, resulting in a 54.9% improvement in average localization accuracy compared with other mainstream localization protocols. The proposed GP-AC algorithm also alleviates the tendency to become trapped in local extrema while maintaining an average localization time of 7.45 s, thereby achieving a favorable balance between localization accuracy and computational overhead. In summary, the proposed method provides a relatively complete closed-loop localization solution with environmental perception and robust adaptive capabilities for underwater localization in complex and uncertain environments. Since the present study assumes that the unknown node remains static or quasi-static during each localization interval, future work will extend the proposed framework to highly dynamic ocean current environments by incorporating Doppler compensation, explicit kinematic models, and spatiotemporal prediction for continuous trajectory tracking of moving nodes. Simultaneously, research on lightweight algorithm design will be further conducted to advance the edge-side deployment and verification of the system in real sea trial platforms. In addition, future work will investigate complete physical blockage of the optical path by developing a link-availability-aware acoustic–optical localization framework that will integrate optical blockage detection, invalid optical measurement exclusion, and acoustic-only fallback localization.

## Figures and Tables

**Table 1:** Hardware and software environment configurations for simulation experiments.

Name	Setting
CPU	Intel(R) Core(TM) Ultra9-185H
Frequency	2.30 GHz
RAM	32.0 GB
Hard drive	1 TB
GPU	NVIDIA GeForce RTX 4060 Laptop GPU
VRAM	8.0 GB
Operating system	Windows 11
Language	MATLAB R2019b

**Table 2:** Key simulation parameter settings.

Parameter Symbol	Parameter Meaning	Default Value/Range
*L* × *W* × *H*	Monitoring area range	100 m × 100 m × 100 m
*f_c_*	Acoustic carrier frequency	25 kHz
*T_s_*	LFM signal duration	20 ms
*B*	LFM signal bandwidth	5 kHz
*β_rms_*	Effective RMS bandwidth	1.443 kHz
*v_s_*	Average sound speed in water	1500 m/s
*v_wind_*	Sea-surface wind speed	0–20 m/s
*s*	Shipping activity factor	0–1, default 0.5
*g_SSP_*	Sound-speed-profile gradient	0.017 s^−1^
*χ_MF_*	Matched-filter calibration factor	1.20
*χ_SSP_*	SSP residual calibration factor	0.10
*σ* ^2^_ac,0_	Acoustic hardware variance floor	0.01 m^2^
*M_cal_*	Number of calibration samples	1000
*λ_opt_*	Optical wavelength	532 nm
*C_Chl_*	Chlorophyll concentration	0.1–4.0 mg/m^3^
γ	Markov discount factor	0.95
σ_f_^2^	GP kernel signal variance	1.0
ℓ_j_	Initial ARD kernel length scale	1.0
σ_TD_^2^	Bellman-observation noise variance	0.01
η_u_	GP uncertainty-exploration coefficient	0.10
N_B_	On-policy batch size	20
ε_F_	Fisher damping coefficient	1 × 10^−3^
η_a_	Actor learning rate	0.01
B_0_	Initial maximum action step	5.0 m
λ_a_	Action-envelope decay rate	0.05
λ_T_	Temporal switching decay rate	2.0
λ_g_	Geometric regularization coefficient	1.0 × 10^−3^
T_max_	Maximum number of exploration steps	100

**Table 3:** Sensor configuration, noise model, sampling rate, motion model, and parameter sources used in the simulator.

Simulated Sensor or Input Module	Noise Model	Sampling Rate	Motion Model	Parameter Source
Acoustic ranging sensor: one-way time-of-arrival ranging based on a linear frequency-modulated signal, with carrier frequency f_c_ = 25 kHz, bandwidth B = 5 kHz, and pulse duration T_s_ = 20 ms.	The Wenz model is used to represent turbulence, shipping, wind-driven, and thermal noise. Thorp absorption loss, matched-filter delay-estimation error, residual sound-speed-profile compensation error, and a hardware-noise floor are also included.	f_s,ac_ = 100 kHz. Each 20 ms LFM pulse contains 2000 discrete samples and is processed by matched filtering to obtain one TOA ranging observation.	A single-epoch static or quasi-static model is adopted. The coordinates of the anchors and the unknown node remain unchanged during one localization interval; velocity, acceleration, and heading states are not included.	The carrier frequency, bandwidth, and pulse duration are consistent with Table 2. The propagation and noise settings follow the LFM matched-filter model, passive sonar equation, Thorp absorption model, Wenz ambient-noise model, and ISSP-based sound-speed-profile correction. The sampling rate is selected to satisfy the Nyquist criterion for the adopted LFM signal.
Underwater optical RSS ranging sensor: Lambertian optical propagation and received-signal-strength-based distance inversion are adopted, with optical wavelength λ_opt_ = 532 nm.	The model accounts for photodetector and receiver-circuit noise, absorption and scattering in water, turbidity-dependent optical attenuation, and distance-domain uncertainty introduced by nonlinear RSS inversion.	f_s,op_ = 10 kHz. A 20 ms RSS observation window contains 200 received-power samples, and the window-averaged power is used for distance inversion.	Continuous attitude variation and optical alignment dynamics are not modeled. Optical ranging is performed only within a short detectable line-of-sight interval.	The optical wavelength and channel parameters are consistent with Table 2. The channel and error settings follow the Lambertian propagation model, Haltrin bio-optical model, receiver-noise model, and Lambert W-based distance inversion. The sampling rate is a simulator-side configuration for the electrical output of the optical receiver.
Environmental sensing inputs: sea-surface wind speed, shipping activity, sound-speed-profile gradient, and chlorophyll concentration.	These environmental variables affect the acoustic noise power spectral density, residual sound-speed-profile error, and optical absorption and scattering coefficients. Independent Monte Carlo scenarios are used to represent environmental variability.	f_s,env_ = 1 Hz. Environmental variables are updated once per second and are held constant during one localization computation.	Not applicable. Variations in environmental inputs do not represent the physical motion of the unknown node.	The parameter ranges are consistent with Refs. [2,3,4,5] and Table 2. Wind speed, shipping activity, sound-speed profile, and chlorophyll concentration correspond to quantities obtainable from surface buoys or meteorological observations, regional vessel-traffic information, sound-speed-depth measurements, and in situ optical or ocean-color observations, respectively. The 1 Hz update rate is a simulator configuration reflecting their slower variation relative to acoustic and optical signals.

**Table 4:** Quantitative analysis of the impact of multi-source environmental factors on heterogeneous ranging standard deviation and complementarity analysis (d = 50 m).

Environmental Scenario	Environmental Parameter Setting	Acoustic Standard Deviation	Optical Standard Deviation	Fused Standard Deviation	Channel State and Fusion Strategy
I. Ideal environment	Calm wind (2 m/s) Clear (0.5 mg/m^3^)	0.30 m	0.05 m	<0.10 m	Optical dominance: Both environmental conditions are optimal. The high-precision optical channel is preferentially locked by the algorithm, achieving centimeter-level ranging.
II. Acoustic interference	Strong gale (14 m/s) Clear (0.5 mg/m^3^)	0.90 m	0.05 m	<0.15 m	Optical compensation: Although acoustic errors are doubled by wind and waves, the water quality remains clear. Acoustic noise is automatically shielded by the fusion model, maintaining high precision.
III. Severe optical degradation	Calm wind (2 m/s) Extremely turbid (2.5 mg/m^3^)	0.30 m	>4.50 m	≈0.32 m	Acoustic-dominant adaptive fusion: An “avalanche” failure occurs in optics due to scattering. The optical weight is substantially reduced by the algorithm, falling back to the stable acoustic channel.
IV. Dual harshness	Strong gale (14 m/s) Extremely turbid (2.5 mg/m^3^)	0.90 m	>4.50 m	≈1 m	Soft degradation: Both channels are damaged. The “less severely degraded” acoustic channel is intelligently selected by the algorithm, limiting the error within a controllable range of 1.0 m.

**Table 5:** Statistical results of 200 independent runs of the proposed model.

Statistical Metric	Optimal Value	Worst Value	Average Value	Standard Deviation	Average Execution Time
RMSE value (m)	0.0632	0.2854	0.1420	0.0510	7.45 s

**Table 6:** Robustness of the proposed GP-AC method under different non-Gaussian noise contamination ratios.

Non-Gaussian Noise Ratio ρ	Mean RMSE (m)	Standard Deviation (m)	95th-Percentile Error (m)	Maximum Error (m)	RMSE Increase Relative to ρ = 0
0%	0.1420	0.0510	0.2280	0.2854	0%
5%	0.1578	0.0576	0.2520	0.3286	11.1%
10%	0.1796	0.0679	0.2890	0.3942	26.5%
20%	0.2287	0.0915	0.3810	0.5368	61.1%
40%	0.3469	0.1482	0.5960	0.8427	144.3%

**Table 7:** Component-wise ablation results of the proposed localization framework. “✓” indicates that the corresponding component is included in the method, whereas “—” indicates that the corresponding component is not included.

Variant	MVUE Weighting	RL Optimizer	GP Uncertainty Bonus	GDOP Reward	NPG	Mean RMSE (m)	Standard Deviation (m)	Average Time (s)
WLS only	—	—	—	—	—	0.962	0.238	1.64
MVUE + WLS	✓	—	—	—	—	0.728	0.176	1.82
MVUE + WLS + RBFGS	✓	—	—	—	—	0.685	0.163	8.10
MVUE + Std-AC	✓	✓	—	—	—	0.512	0.132	7.15
GP-AC without uncertainty bonus	✓	✓	—	✓	✓	0.228	0.078	7.23
GP-AC without GDOP reward	✓	✓	✓	—	✓	0.267	0.091	7.18
GP-AC without NPG	✓	✓	✓	✓	—	0.191	0.064	7.39
Full GP-AC	✓	✓	✓	✓	✓	0.142	0.051	7.45

**Table 8:** Comparison with classical nonlinear estimation and optimization methods under identical MVUE-fused acoustic–optical observations.

Method	Method Category	Mean RMSE (m)	Standard Deviation (m)	95th-Percentile Error (m)	Average Time (s)
MVUE-EKF	First-order nonlinear estimation	0.604	0.153	0.856	2.21
MVUE-UKF	Sigma-point nonlinear estimation	0.492	0.128	0.703	2.86
MVUE-PF	Sequential Monte Carlo estimation	0.447	0.119	0.643	6.73
MVUE + WLS + RBFGS	Gradient-based nonlinear optimization	0.685	0.163	0.953	8.10
Full GP-AC	RL-based coordinate optimization	0.142	0.051	0.226	7.45

## Data Availability

Data are available upon request from the corresponding author.

## Appendix A

**Table A1:** Definitions of Key Symbols Used in Section 4 and Section 5.

Symbol	Meaning
*d_ij_*	True physical distance between nodes i and j.
*z_ac_*	Bias-compensated acoustic ranging observation entering the fusion stage.
*σ_ac_* ^2^	Environment-dependent residual acoustic ranging error variance.
*v_wind_*	Sea-surface wind speed used in the acoustic uncertainty model.
*s*	Normalized shipping-activity factor.
*g*_SSP_	Sound-speed-profile gradient used in acoustic ray-bending compensation.
*z_op_* ^(0)^	Optical ranging observation obtained through nonlinear RSS-to-distance inversion before second-order bias correction.
*z_op_*	Bias-corrected optical ranging observation entering the fusion stage.
*σ_op_* ^2^	Environment-dependent equivalent optical ranging error variance after nonlinear uncertainty propagation.
*C_chl_*	Chlorophyll concentration used to characterize water turbidity.
*c*(*λ*, *J*)	Beam attenuation coefficient determined by the optical wavelength and Jerlov water type.
*λ*_1_, *λ*_2_	Conditional minimum-variance weights assigned to the acoustic and optical observations, respectively.
*d_fuse_*	MVUE-fused acoustic–optical ranging estimate.
*d_fuse,i_*	MVUE-fused ranging observation associated with anchor link i.
*σ_fuse_* ^2^	Conditional variance of the fused ranging error.
*σ_total,i_* ^2^	Total fused ranging error variance associated with anchor link i.
***X*** = [*x*, *y*, *z*]^T^	Three-dimensional coordinate vector of the unknown underwater node.
***A****_i_* = [*x_i_*, *y_i_*, *z_i_*]^T^	Known coordinate vector of the *i*-th anchor node.
*N*	Number of available anchor nodes.
*e_i_*(***X***)	Spatial ranging residual between candidate coordinate X and anchor i.
*ω_i_*	WLS weight of anchor link i, defined as the inverse of its total fused ranging error variance.
***W***	Diagonal WLS weight matrix constructed from the anchor-link weights.
*f*(***X***)	Weighted least-squares objective function for three-dimensional coordinate estimation.
*Ω*	Feasible underwater localization region used to constrain candidate coordinates.
***M*** = ⟨*S*, *A*, *P*, *R*, *γ*⟩	Continuous-space Markov decision process used for sequential coordinate refinement.
*s_t_*	Composite state at iteration t, containing the current candidate coordinate and weighted ranging residuals.
***p*** *_t_*	Current three-dimensional candidate coordinate of the GP-AC agent.
***Ψ***(***p****_t_*)	Vector of WLS-weighted acoustic–optical ranging residuals evaluated at the current candidate coordinate.
***a*** *_t_*	Continuous three-dimensional coordinate-increment action at iteration t.
*B_t_*	Dynamic upper bound imposed on the coordinate-increment action at iteration t.
*π_θ_*(***a****_t_*|*s_t_*)	Continuous Gaussian Actor policy.
***θ***	Parameter vector of the Actor policy.
*V*(*s*)	State-value function estimated by the GPTD Critic.
*k*(*s_i_*, *s_j_*)	Squared-exponential ARD kernel measuring the similarity between two composite states.
***D*** *_t_*	Online GPTD state dictionary maintained at iteration t.
***m****_t_*, ***P****_t_*	Posterior mean vector and posterior covariance matrix of the online GPTD Critic.
*σ_V,t_*^2^(*s*)	GPTD posterior value variance representing epistemic uncertainty at state s.
*A*_GP_(*s_t_*, ***a**_t_*)	Uncertainty-compensated scalar advantage used for the Actor update.
***F*** *_π,t_*	Damped empirical Fisher information matrix of the Actor policy.
*L*_base_(*s_t_*)	Generalized Huber algebraic penalty constructed from the fused-distance WLS residuals.
*H_δ_*(*e*)	Generalized Huber loss applied to an individual ranging residual.
***J***(***p**_t_*)	Direction-cosine Jacobian matrix evaluated at the current candidate coordinate.
***I***_geo_(***p****_t_*)	Weighted geometric information matrix used for the GDOP calculation.
*L*_geo_(*s_t_*)	GDOP-based geometric topology penalty at state $s_{t}$.
*β*(*s_t_*, *t*)	Dynamic switching coefficient between the algebraic and geometric topology penalties.
*r_t_*	Composite immediate reward combining the algebraic and geometric topology and boundary penalties.
***X****	Final estimated three-dimensional coordinate of the unknown node.
